# Proteases Underground: Analysis of the Maize Root Apoplast Identifies Organ Specific Papain-Like Cysteine Protease Activity

**DOI:** 10.3389/fpls.2019.00473

**Published:** 2019-04-30

**Authors:** Jan Schulze Hüynck, Farnusch Kaschani, Karina van der Linde, Sebastian Ziemann, André N. Müller, Thomas Colby, Markus Kaiser, Johana C. Misas Villamil, Gunther Doehlemann

**Affiliations:** ^1^Center of Excellence on Plant Sciences (CEPLAS), Botanical Institute, University of Cologne, Cologne, Germany; ^2^Max Planck Institute for Plant Breeding Research, Cologne, Germany; ^3^Max Planck Institute for Terrestrial Microbiology, Marburg, Germany; ^4^Institute of Chemical Biology, University of Duisburg-Essen, Essen, Germany

**Keywords:** root, apoplast, PLCP, organ specific, salicylic acid

## Abstract

Plant proteases are key regulators of plant cell processes such as seed development, immune responses, senescence and programmed cell death (PCD). Apoplastic papain-like cysteine proteases (PL) are hubs in plant-microbe interactions and play an important role during abiotic stresses. The apoplast is a crucial interface for the interaction between plant and microbes. So far, apoplastic maize PL and their function have been mostly described for aerial parts. In this study, we focused on apoplastic PLCPs in the roots of maize plants. We have analyzed the phylogeny of maize PLCPs and investigated their protein abundance after salicylic acid (SA) treatment. Using activity-based protein profiling (ABPP) we have identified a novel root-specific PLCP belonging to the RD21-like subfamily, as well as three SA activated PLCPs. The root specific PLCP CP1C shares sequence and structural similarities to known CP1-like proteases. Biochemical analysis of recombinant CP1C revealed different substrate specificities and inhibitor affinities compared to the related proteases. This study characterized a root-specific PLCP and identifies differences between the SA-dependent activation of PLCPs in roots and leaves.

## Introduction

Proteases determine a variety of biological processes ranging from organ maturation, senescence and programmed cell death (PCD) (van der Hoorn and Jones, 2004; van der Hoorn, 2008). They perform cleavage of substrates into small fragments by catalyzing peptide bond hydrolysis. Proteases are classified into four main classes according to their catalytic site: cysteine proteases, serine proteases, aspartic proteases, and metalloproteases (Rawlings et al., 2018). Cysteine proteases are further subdivided into 14 super families, each using the catalytic triad or dyad in a different structural fold, representing convergent evolution of the catalytic mechanism (Rawlings et al., 2018). In this study we are focusing on papain-like cysteine proteases (PLCP). PLCPs are classified into clan CA based on their structural similarity to papain and conserved catalytic residues (Rawlings et al., 2018). They are divided into family C1B (cytosolic) and C1A (apoplastic) and further subdivided into nine subfamilies based on phylogeny (Richau et al., 2012). PLCPs are known to be involved in growth related senescence (Noh and Amasino, 1999a; McLellan et al., 2009), PCD (Gilroy et al., 2007; Coll et al., 2011; Lampl et al., 2013), predicted to be important for resource acquisition (Adamczyk et al., 2010) and act as hubs in plant immunity, where they are involved in the perception of microbes, initiation of signaling cascades and activation of responses against pathogens (Misas-Villamil and van der Hoorn, 2008; Jashni et al., 2015; Misas-Villamil et al., 2016). Due to their crucial roles in the regulation of various cellular processes, PLCP activity is tightly controlled via autocatalytic posttranslational modifications, as well as by endogenous inhibitors such as cystatins and serpins (Martinez and Diaz, 2008; Ochieng and Chaudhuri, 2010; Martínez et al., 2012; van der Linde et al., 2012a; Lampl et al., 2013). PLCPs carry a signal peptide important for their transport to the apoplast as well as an auto inhibitory prodomain prior to the active C1-protease domain. Some members of the subfamily 1 (RD21) and 4 (XBCP3) contain a granulin domain sharing homology with granulins/epithelin which are growth hormones in animals, released after wounding (Bateman and Bennett, 1998, 2009; Richau et al., 2012). PLCPs contain the conserved catalytic triad Cys, His, Asn. Their general enzymatic activity involves a nucleophilic attack of the thiol-group at the substrate carboxyl-terminus where His acts as a proton acceptor (base) for the catalytic Cys and Asn plays an important role for the orientation of the His (Rawlings et al., 2018).

Maize is one of the most important crop plants. It does not only play an important role for human consumption but also for feeding of livestock and the production of biofuels as an alternative to petrol (FAO, 2012; Ranum et al., 2014). Different models predict that due to climate change the yield of maize might decrease by 2055 up to 10% in some areas like Africa (Jones and Thornton, 2003). This loss has to be compensated by improvements in plant breeding and pest control. To be able to cope with pests we need a better understanding of the interaction between plants and its associated microbes both, in the phyllosphere and the rhizosphere. Maize associates with a variety of microbes, which might lead to either beneficial effects on plant growth such as the interaction with arbuscular mycorrhizal fungi (Subramanian et al., 2013; Bárzana et al., 2014) or to tremendous damage on the plant such as the interaction with the biotrophic fungus *Ustilago maydis* (Brefort et al., 2009) or the necrotrophic pathogen *Fusarium verticillioides* (Duncan and Howard, 2009; Barreau et al., 2010).

Most plants and microbes interact via the apoplast, which contains different types of defense components such as cysteine proteases and toxic metabolites (Ökmen and Doehlemann, 2016). Previously, we have demonstrated the importance of maize apoplastic leaf PLCPs for plant immunity and during *U. maydis* infection. We found an endogenous cystatin to be able to suppress host immunity acting as a compatibility factor (van der Linde et al., 2012a,b), a fungal effector that inhibits apoplastic PLCPs in order to suppress defense responses (Mueller et al., 2013) and an endogenous peptide that requires PLCP activity for its release from the propetide molecule, leading to activation of salicylic acid (SA) defense signaling (Ziemann et al., 2018).

In this study, we investigate apoplastic PLCPs in maize roots. Using a proteomics approach, we have identified different PLCPs activated by SA treatment in roots. Moreover, we have identified and biochemically characterized the novel root specific PLCP, CP1C. In comparison to the well characterized CP1A and CP1B proteases, CP1C shows a distinct substrate specificity and inhibitory profile, albeit their structure and sequence similarities.

## Results

### Leaf and Root Apoplastic Proteomes Show Differential PLCP Activities

**FIGURE 1:**
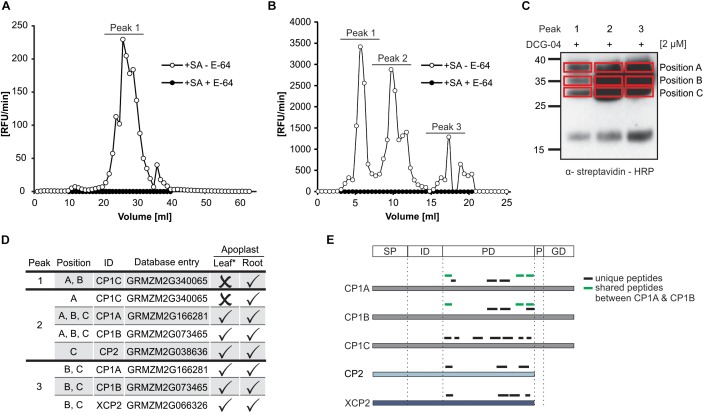
Identification of maize root PLCPs after ion-exchange chromatography. **(A)** Leaf apoplastic fluid (LAF) and **(B)** root apoplastic fluid (RAF) of maize leaves or roots, pre-treated with 2 mM salicylic acid (+SA) were isolated and fractionated using ion exchange chromatography (IEC). Fractions were tested for PLCP activity using 10 μM of the substrate Z-FR-AMC with (+) or without (-) 2 μM E-64. The release of AMC (relative fluorescent unit = RFU) per minute was measured and plotted against fraction volume. Peaks represent fractions of higher PLCP activity that were inhibited when using E-64. **(C)** DCG-04 labeling of fractions corresponding to different peaks **(B)** were pooled and labeled with 2 μM DCG-04 for 2 h. Samples were separated by SDS-PAGE and detection of biotinylated proteins was performed using an α-streptavidin-HRP antibody. Red squares mark samples subjected to in gel digest (IGD) and MS analysis. Peaks 1, 2, and 3 correspond to pooled fractions **(B)** and position A, B, and C mark size separated signals of each peak loaded. **(D)** Identification of apoplastic root PLCPs and comparison with leaf proteome. Apoplastic root-PLCPs identified by MS analysis were compared to PLCPs previously published in leaf apoplastic fluid by van der Linde et al., 2012a (^∗^). Peak and position **(B,C)** of PLCPs identified in this study as well as PLCP presence (✓) or absence (×) is indicated. **(E)** Analysis of peptides found by mass spectrometry. Displayed is a schematic representation of the identified PLCPs and the position of peptides found: SP, signal peptide; ID, autoinhibitory prodomain; PD, protease C1-domain; P, proline-rich domain; GD, granulin domain. Unique peptides are labeled in black and shared peptides are labeled in green.

The maize genome encodes 52 PLCP (Rawlings et al., 2018) localized in different compartments such as cytoplasm, vacuoles, vesicles and apoplast. In leaf proteomes, SA has been described to activate apoplastic PLCPs (van der Linde et al., 2012a). Root apoplastic fluids (RAF) of maize seedlings treated with SA were isolated to analyze the effect of SA on the activation of root PLCPs. Fractionation of RAF by ion exchange chromatography followed by an *in vitro* activity assay using the substrate Z-FR-AMC showed one distinct peak corresponding to leaf apoplastic PLCPs (Figure 1A) and three distinct peaks representing elevated PLCP activities in roots (Figure 1B). Pre-treatment of leaf and root apoplastic proteomes with the specific PLCP inhibitor E-64 abolished this activity (Figure 1A,B). Interestingly, the observed activity pattern of the root apoplast significantly differs from the leaf apoplast since the leaf proteome shows 10–20-fold lower activity compared to RAF (Figure 1A,B). In a following step, protein fractions corresponding to the three major peaks observed in Figure 1B were pooled and active PLCPs were labeled using DCG-04, a probe that binds covalently and irreversible to the active site of PLCPs allowing us to monitor the availability of active sites rather than their abundance (Greenbaum et al., 2000; van der Hoorn et al., 2004). Taking advantage of the biotin tag present in DCG-04, a pull down purification of labeled proteins was performed. Signals corresponding to labeled proteins of different molecular weights, were excised from the gel and subjected to an in – gel digest (IGD) mass spectrometry analysis (Figure 1C, position A–C). Five apoplastic PLCPs have been identified (Figure 1D). The two CP1-isoforms, CP1A and CP1B, as well as CP2 and XCP2 were detected in roots, correlated to previous identification in the leaf apoplast (van der Linde et al., 2012a). In addition, we found a third CP1-like PLCP, CP1C that has not been previously identified in leaves (Figure 1D). CP1C was the only PLCP found in position A and B of peak 1 and it was additionally found in position A of peak 2. In contrast, CP1A and CP1B were not found in peak 1 but in all positions of peak 2 as well as in position B and C of peak 3. The fact that CP1C was fractionated at different volumes than CP1A and CP1B might indicate distinct biochemical properties of CP1C compared to the other two isoforms. All identified unique peptides were located in the predicted protease C1 domain (CD) and confirms the success of the DCG-04 pull down targeting active proteases (Figure 1E). Taken together, the comparison of active PLCPs in leaf- and root proteome revealed the presence of the four PLCPs CP1A, CP1B, CP2, and XCP2, previously identified in the leaf apoplast (van der Linde et al., 2012a), as well as one additional root specific PLCP: CP1C.

**FIGURE 2:**
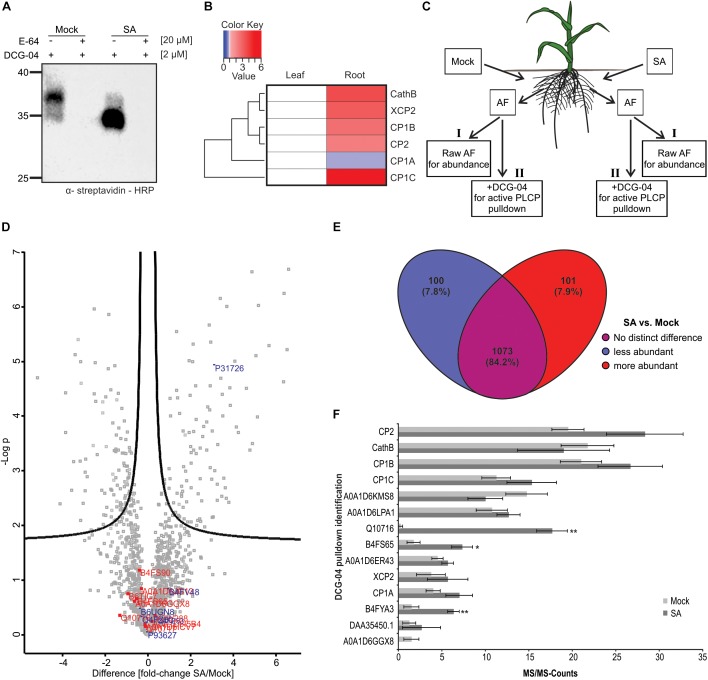
Abundance and activity of root apoplastic PLCPs after SA treatment. **(A)** DCG-04 labeling of root apoplastic fluid (RAF) pre-treated with 2 mM salicylic acid or mock. RAF was preincubated for 30 min either with 20 μM E-64 or DMSO. Then samples were labeled with 2 μM DCG-04 for 2 h and analyzed on a SDS-gel. Biotinylated proteins were detected using an α-streptavidin-HRP antibody. **(B)** Expression pattern of six apoplastic PLCPs. Relative expression of root apoplastic maize PLCPs in untreated B73 based on publicly available data (Winter et al., 2007; Sekhon et al., 2011; Andorf et al., 2016; Stelpflug et al., 2016). Mean expression of leaves and roots at different developmental stages was calculated and normalized to leaf expression for individual PLCPs. The heat map represents a one – to – one comparison for each PLCP. PLCPs were clustered based on their relative expression pattern to leaves. **(C)** Schematic overview of MS experimental setup. Roots of maize plants were treated with 2 mM SA or mock. Apoplastic fluid of four biological replicates was isolated 12 h after treatment. One part of the apoplastic fluid was used for shotgun analysis to investigate protein abundance (I) and the other part was used for a DCG-04 pull down of labeled PLCPs followed by OBD (II). Both, samples I and II, were subjected to mass spectrometry analysis for protein identification and quantification. Modified from Guillaume (2017). **(D)** Protein abundance in roots after SA treatment. A comparison of protein abundance between mock- and SA-treated RAFs is displayed in a volcano plot. Fold change differences between treatments against negative log *p*-value is plotted. Cysteine proteases are labeled in red and cysteine protease inhibitors are labeled in blue. **(E)** Comparison of protein abundance after SA treatment. Changes in protein abundance after SA treatment compared to mock-treated plants were displayed using a Venn diagram. Total numbers of identified proteins and percentages are indicated. Significantly less abundant proteins in SA-treated samples are labeled in blue. No significant differential proteins are labeled in purple and significantly more abundant proteins are shown in red. **(F)** Activation of PLCPs after SA treatment. DCG-04 labeled samples were analyzed and compared between treatments. MS/MS-counts were plotted against identified PLCPs in both treatments. MS/MS-counts of SA treatments are labeled in dark gray and MS/MS-counts of mock-treated samples are labeled in light gray. The experiments were performed using four independent biological replicates. Error bars represent the SEM. *P*-values were calculated with an unpaired *t*-test. ^∗^*P* < 0.05; ^∗∗^*P* < 0.01.

We further aimed to characterize the novel root PLCP CP1C and shed light on its role during SA signaling in the root apoplast. We labeled RAF from plants pre-treated with 2 mM SA or mock using DCG-04. Besides an increase in activity, we also noticed a shift into lower molecular weight signals after treatment with SA, suggesting that some PLCPs become less active, while others are activated through SA treatment (Figure 2A). To confirm the observed root specificity we also examined the expression pattern of CP1C in comparison to the other four MS-detected PLCPs and Cathepsin B (CathB) which has been previously identified in leaves (van der Linde et al., 2012a). Using publicly available B73 expression data of untreated maize leaves and roots (maizegdb.org), PLCP expression patterns were displayed using a heat map in which root gene expression was normalized to leaves (Figure 2B). Overall, apoplastic PLCPs seem to be higher expressed in roots compared to leaves, which correlates with the higher enzyme activity level observed in roots compared to leaves (Figure 1A,B). CP1B, CP2, XCP2, and CathB show a slightly higher expression level in roots, whereas CP1A expression is slightly stronger in leaves (Figure 2B). CP1C transcripts are detected in leaves, but its expression is about sixfold higher in roots (Figure 2B). Remarkably, of all six PLCPs, CP1C shows the strongest differential expression in roots compared to leaves. To understand if the expression levels found for the PLCPs correlate with their abundance and their activity we performed shot-gun analysis together with a DCG-04 pull down. We describe abundance as the total pool of proteins, active or inactive present in the proteome. Roots of maize seedlings were treated with mock or SA. After 2 days root apoplastic fluid was isolated and one part was used for shot-gun analysis and the other part was labeled with DCG-04 (Figure 2C). A comparison between protein abundance in mock vs. SA treated samples has been represented using a volcano plot (Figure 2D). Several proteins related to the SA pathway such as thioredoxins, shikimate biosynthesis and pathogenesis related PR10 protein increased their abundance in the SA treated samples, confirming a successful SA treatment (Supplementary Table 1; Tada et al., 2008; Chen et al., 2010; Dempsey et al., 2011). Interestingly, we found that the abundance of the cystatin P31726, an endogenous cysteine protease inhibitor, was increased almost fourfold after SA treatment whereas the abundance of other cysteine protease inhibitors did not change (Figure 2D, blue). Remarkably, the total abundance of PLCPs did not change after SA treatment (Figure 2D, red) suggesting a posttranslational activation (Figure 2A). The majority of proteins (84.2%) do not change significantly in abundance upon SA treatment but 7.9% show differential behavior being significantly more abundant in the apoplast after SA treatment (Figure 2E). To get more insight into the SA-effect on activation of apoplastic PLCPs we performed a DCG-04 pull down of SA treated and mock plant AFs followed by an on bead digest (OBD) and mass spectrometry analysis. MS/MS counts were plotted against identified proteins (Figure 2F). The majority of peptides found in this pull down correspond to PLCPs (Supplementary Table 2) confirming an enrichment of those proteases after DCG-04 labeling. We found again peptides for CP1A, CP1B, CP1C, CP2, and XCP2 in agreement with the previously described IGD made from samples separated by ion-exchange chromatography (IEC) (Figure 1E, 2F). Additionally, we found CathB, which has so far not been identified in the previous MS analysis made for maize root apoplast (Figure 1D,E, 2F) likely, due to its isoelectric point of 5.49 close to the conditions used for the IEC (pH 6). Remarkably, the activities of the previously characterized PLCPs CP1A, CP1B, CP1C, CP2, XCP2, and CathB and five additionally detected PLCPs do not change significantly after SA treatment compared to mock. In contrast, three other PLCPs were identified with significantly increased activity, up to 70-fold, after SA treatment: B4FS65 belonging to the cysteine protease superfamily, B4FYA3 a xylem bark cysteine peptidase and Q10716 a cysteine proteinase 1 (Figure 2F and Supplementary Figure 1). With these experiments, we demonstrate the presence of the previously leaf identified PLCPs, CP1A, CP1B, CP1C, CP2, and XCP2 in the root apoplast and additionally, we identify CathB together with other eight PLCPs, which have not been previously found in the IEC root apoplast analysis. Three of the newly identified PLCPs seem to be activated upon SA treatment.

**FIGURE 3:**
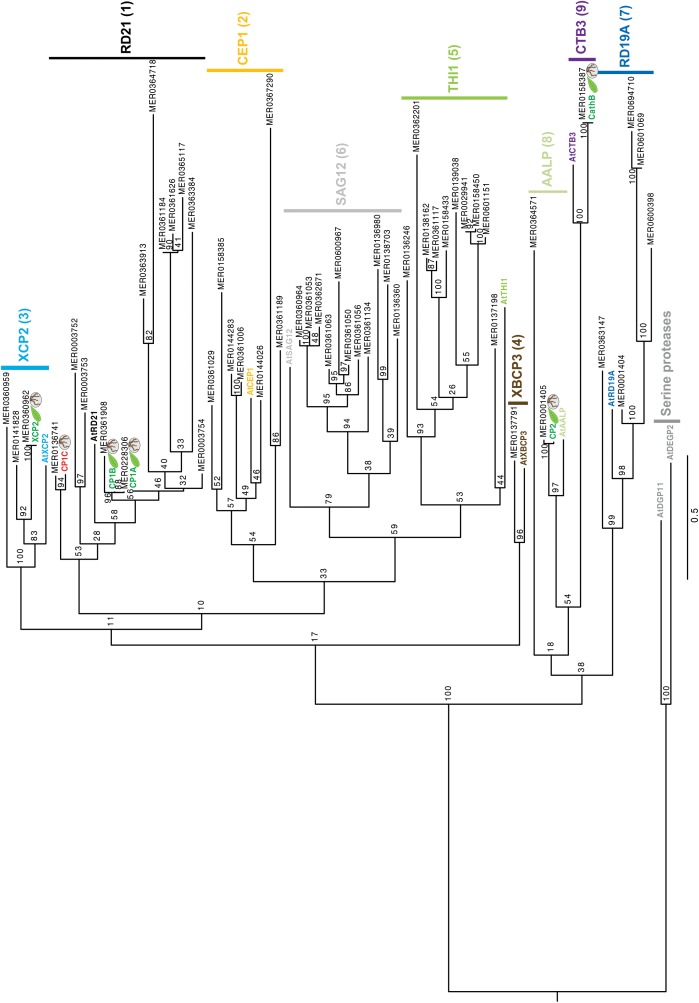
Phylogeny of apoplastic maize-PLCPs. 52 maize PLCP sequences of the line B73 retrieved from the MEROPS database (Rawlings et al., 2018) as well as our six identified PLCPs from Early golden Bantam (EGB) were used to generate a phylogenetic tree.Additionally, we included one type member of each PLCP subfamily of *A. thaliana* and two serine proteases DEGP2 and DGP11 from *A. thaliana* as outgroup (Beers et al., 2004; Richau et al., 2012). For the phylogenetical analysis we used full length sequences including signal peptide, prodomain, protease C1-domain and, if present, granulin domain as was described before in Richau et al. (2012). The tree is drawn to scale, with branch lengths measured in the number of substitutions per site. Sequences were aligned using MAFFT (v7.407) (Katoh and Standley, 2013; Supplementary Table 5). RAxML with the GTRGAMMA substitution model (v8.2.0) was used for the construction of the tree (Stamatakis, 2014). The robustness was assessed using 100 bootstrap replicates. Apoplastic EGB maize PLCPs were highlighted according to the organs they were found in: leaves (green) roots (red). Numbers indicate the PLCP subfamilies based on Richau et al. (2012).

To get insights into the subfamily classification of the newly identified root apoplastic proteases we evaluate the sequence similarity of maize apoplastic PLCPs using phylogeny. A total of 52 maize PLCP sequences from B73 retrieved from the MEROPS database (Rawlings et al., 2018) and our six identified PLCPs from Early Golden Bantam (EGB) were used to generate a phylogenetic tree with the maximum likelihood method. Additionally, for the subfamily classification we included one type member of each PLCP subfamily of *A. thaliana* (Richau et al., 2012). Two serine proteases from *A. thaliana* (AtDGP11 and AtDEGP2) were used as outgroup (Beers et al., 2004; Richau et al., 2012). PLCPs were classified into nine subfamilies: RD21 (1), CEP1 (2), XCP2 (3), XBCP3 (4), THI1 (5), SAG12 (6), RD19A (7), AALP (8), and CTB3 (9). The largest group of maize PLCPs belongs to the RD21 subfamily (12 members), followed by members of the SAG12 subfamily (11 members) and the THI1 subfamily (10 members). Other PLCP subfamilies are represented by few members (Figure 3). All identified apoplastic CP1-like PLCPs, CP1A, CP1B, and CP1C carrying a granulin domain, cluster together into the subfamily 1 of RD21, whereas CathB, CP2, and XCP2 grouped into the subfamilies CTB3, AALP, and XCP2, respectively (Figure 3). The SA activated PLCP B4FS65 (MER036246) belongs to the THI1 subfamily, whereas Q10716 (MER0001404) was found to be present in the RD19A subfamily (Figure 3). Sequence alignment of the SA activated PLCP B4FYA3 (MER0137791) showed high sequence similarity to CP14 belonging to subfamily XBCP3. B4FYA3 is homolog to *Nicotiana benthamiana* and *N. tabaccum* CP14, proteins described to be involved in PCD (Zhao et al., 2013; Paireder et al., 2016).

Altogether, we showed that PLCP expression and activity in root is higher than in leaf apoplast. A comparison of root PLCP abundance vs. activity after SA treatment indicates that PLCP activation likely occurs posttranslationally. Furthermore, we have identified three SA-activated root PLCPs, not previously detected in leaves, suggesting a different mechanisms of SA signaling through PLCPs in different organs.

### CP1C Is a Root Specific Apoplastic PLCP

**FIGURE 4:**
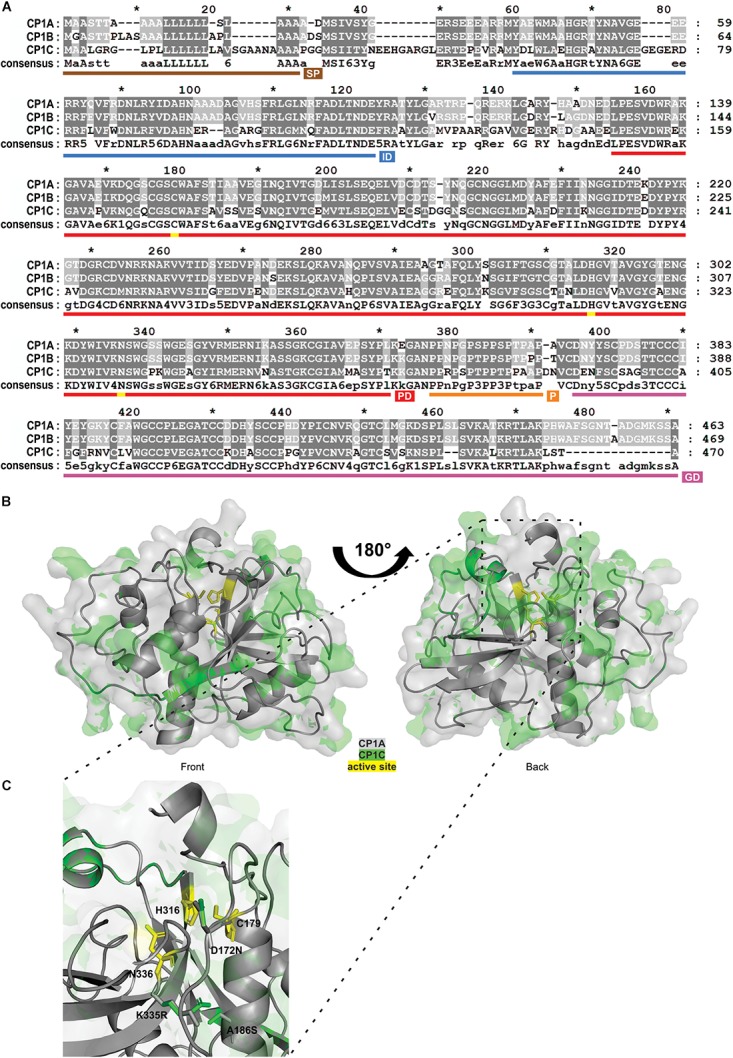
Sequence and structural comparison of maize apoplastic CP1-like PLCPs. **(A)** Sequence homology between CP1-like PLCPs. Amino acid sequences of apoplastic CP1-like PLCPs: CP1A, CP1B, and CP1C of the maize line EGB were aligned to evaluate their sequence conservation. Dark gray background indicates conserved amino acids among all three PLCPs, light gray background indicates similar amino acids among two PLCPs and white background indicates different amino acids. Signal peptide (SP, brown), autoinhibitory prodomain (IP, blue), protease C1-domain (PD, red), proline-rich domain (P, orange), and granulin-domain (GD, purple) were predicted. Amino acids forming the catalytic triad C179, H316, N336 are labeled in yellow. **(B)** Structure similarities between CP1A and CP1C. A 3D-model of superimposed mature CP1A and CP1C was generated. CP1A (gray) and CP1C (green) from EGB were modeled without signal peptide, autoinhibitory prodomain and granulin-doman using Phyre2 (Kelley et al., 2015) based on the crystal structure of caricain PDB: 1pciA (Groves et al., 1996). The catalytic triad C179, H316, N336 is indicated in yellow. **(C)** Close-up of the catalytic triad of superimposed CP1A and CP1C. Differences in the catalytic grooves of CP1A and CP1C (B) were examined. The catalytic triad C179, H316, N336 is indicated in yellow. Amino acid differences D172N, A186S, and K335R were modeled from CP1A (gray) to CP1C (green).

Granulin containing PLCPs of the subfamily 1, such as Mir1 from maize or RD21 from Arabidopsis are known to play crucial roles related to plant defense and senescence (Lopez et al., 2007; Shindo et al., 2012). Here, we have identified CP1C, a root specific granulin containing PLCP closely related to CP1A and CP1B. All three CP1-like proteases are apoplastic localized, consistent with their higher activity at low pH (Supplementary Figure 4). Sequence analysis of mature CP1C compared to CP1A and CP1B revealed high similarities, 74% identity, at the amino acid level (Figure 4A). All three proteases contain a predicted N-terminal secretion signal, an autoinhibitory prodomain and a C-terminal granulin domain (Figure 4A). CP1C catalytic triad consists of three main residues: C179, H316, and N336, as well as Q173, which is believed to stabilize the oxyanion during the catalytic reaction (Figure 4A,C). We observed sequence variation between predicted domains, e.g., signal peptide and autoinhibitory prodomain and between autoinhibitory prodomain and protease C1-domain and at the C-terminal granulin domain (Figure 4A). To further analyze CP1C at the structural level a three dimensional model was predicted based on caricain (PDB: 1pciA) (Groves et al., 1996; Kelley et al., 2015). An overlay of the models predicted for the mature CP1A and CP1C was performed. The majority of residue changes appeared to be located on the surface of the proteins (Figure 4B). Out of 53 different surface residues between CP1A and CP1C 25 were predicted to cause a minor impact for the structure due to similar biochemical properties. Of all changes, only three amino acids were located inside CP1C: D172N, A186S, and K335R. All three amino acids are predicted to be located close to the active site (Figure 4C). Interestingly, the catalytic groove seems to be narrower in CP1C compared to CP1A. A different orientation of the basic amino acids K335R close to N336 between CP1A and CP1C might explain the distinct catalytic properties and substrate preferences (Figure 4C). Altogether, CP1A and CP1C seem to share similar sequence homology and structure although differences on the surface of CP1C might result into different interaction partners.

**FIGURE 5:**
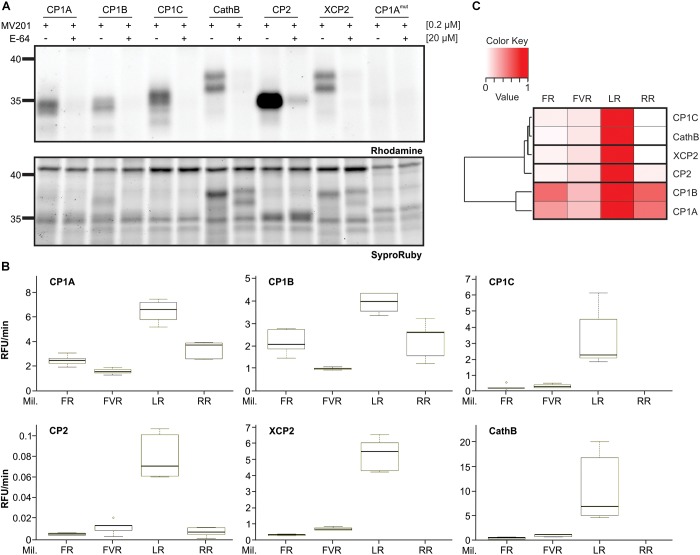
Differential substrate specificities of apoplastic PLCPs. **(A)** Activity of heterologous expressed maize apoplastic PLCPs. Leaf apoplastic fluids of *N. benthamiana* overexpressed PLCPs: CP1A, CP1B, CP1C, Cathepsin B (CathB), CP2, XCP2 and the catalytic inactive CP1A mutant CP1A^mut^ were isolated and labeled with the probe MV201 (Richau et al., 2012). Samples were pre-incubated for 30 min either with 20 μM E-64 (+), a covalent and irreversible PLCP inhibitor (Barrett et al., 1982), or DMSO (-) followed by 2 h labeling with 0.2 μM MV201 (Richau et al., 2012). Labeled PLCPs were visualized by fluorescence scanning (Ex. 532 nm, Em. 580 nm). SyproRuby-stain (Ex. 450 nm, Em. 610 nm) was performed for visualization of sample loading. **(B)** Substrate cleavage assays of PLCPs using different synthetic substrates. Signal quantification from **(A)** was used to normalize for equal amounts of active PLCPs in this assay. Overexpressed PLCPs were tested in substrate cleavage assays using 10 μM of the following substrates: Z-FR-AMC (FR), BZ-FVR-AMC (FVR), Z-LR-AMC (LR), and Z-RR-AMC (RR). The release of AMC (relative fluorescent unit = RFU) per minute was calculated and plotted against each substrate. The box signifies the upper (Q3) and lower (Q1) quartiles, and the median is represented by a short black line within the box for each substrate. Lower and upper whiskers represent Q1-1.5 × IQR and Q3+1.5 × IQR, respectively. This experiment was performed using three independent biological replicates each with technical duplicates. **(C)** Heat-map of relative substrate affinity. PLCP affinity for each substrate used in **(B)** was calculated relative to the strongest activity. The P2-position preference was evaluated relatively to the strongest activity (set to 1, red) to no activity (set to 0, white). PLCPs were clustered based on their relative substrate affinity pattern.

To study if CP1C also shares biochemical properties with other apoplastic PLCPs, found in roots and leaves, we analyzed their substrate specificity. CP1A, CP1B, CP1C, XCP2, CP2, and CathB were transiently overexpressed in *N. benthamiana* using Agrobacterium and after 3 days apoplastic fluids were isolated and tested for their activity using the activity-based probe MV201 (Richau et al., 2012; Figure 5A). Additionally, the catalytic inactive mutant of CP1A^mut^ was used as a negative control, as well as overexpressed cytosolic GFP. Both, CP1A^mut^ and GFP allowed us to differentiate the endogenous activity of *N. benthamiana* PLCPs from the overexpressed maize PLCPs (Figure 5A and Supplementary Figure 2). Apoplastic fluids were tested in a substrate – cleavage assay using 10 μM of four synthetic substrates coupled to a 7-amino-methyl-coumarin (AMC): Phe-Arg-AMC (FR), Phe-Val-Arg-AMC (FVR), Leu-Arg-AMC (LR) and Arg-Arg-AMC (RR). Activity was then normalized to the CP1A^mut^ and the GFP-control. All these substrates differ in their residue at the P2-position which has been previously identified to be crucial for PLCP activity (Turk et al., 1995; Paireder et al., 2016, 2017). PLCP activities were normalized to the highest activity tested (set to 1) and were represented in a heat-map (Figure 5C). We observed that all overexpressed PLCPs show a preferred cleavage activity for the substrate LR (Figure 5B,C). The basal PLCP activity of *N. benthamiana* also shows LR cleavage preference although with reduced levels in comparison to the overexpressed samples (Supplementary Figure 2). CP1A and CP1B also cleave RR, FR, and FVR despite CP1B slightly preference for FR. Strikingly, the root specific CP1C differs in the substrate cleavage preference from CP1A and CP1B. It mostly processes the LR-substrate displaying only trace amounts of activity toward other substrates. This LR unique cleavage preference resembles the cleavage specificities of XCP2 and CathB (Figure 5B,C). CP2 shows generally very low cleavage activity toward the tested substrates (Figure 5B), although it is active and highly overexpressed in *N. benthamiana* (Figure 5A). The low cleavage activity of CP2 indicates distinct substrate specificities for this protease in comparison to the other tested apoplastic PLCPs.

**FIGURE 6:**
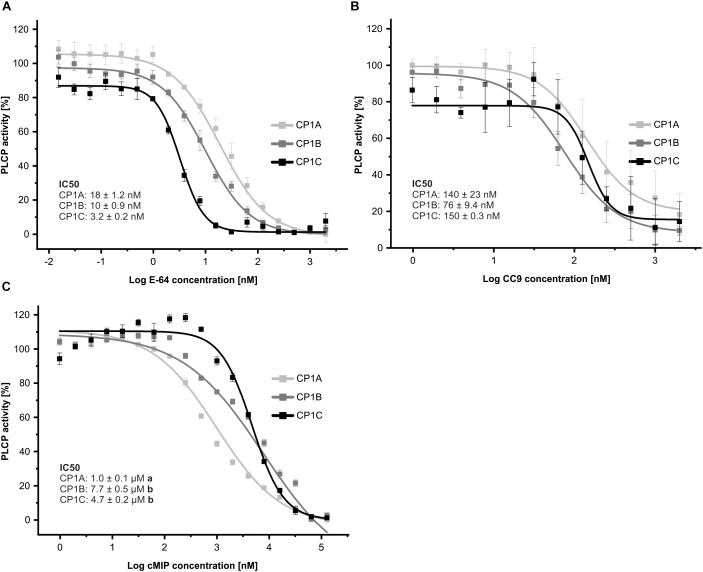
CP1-like proteases show distinct inhibitory profiles. Apoplastic fluids of *N. benthamiana* overexpressed CP1A, CP1B, and CP1C were evaluated for their activity using 10 μM of the substrate Z-LR-AMC (LR). The inhibitory profile for E-64 **(A)**, CC9, an endogenous cystatin **(B)** and cMIP, a conserved microbial inhibitor of proteases **(C)** was tested. We used equal amounts of active PLCPs based on signal quantification from MV201 labeling of apoplastic fluids (Supplementary Figure 3). Inhibitor concentrations ranged from 15 pM to 128 μM. Activity was set to 100% in the absence of inhibitor. Normalized values were plotted against Log of inhibitor concentration in nM. The experiment was performed in three independent biological replicates each with technical duplicates. A nonlinear fit based on a dose response function was performed and IC50-values were calculated. Error bars represent the SEM. Significance was calculated using an unpaired *t*-test and differing letters behind the IC50-values indicate significant differences (α < 0.05).

As a second approach to the biochemical characterization of CP1C, we tested the inhibitory profile of apoplastic CP1-like PLCPs toward characterized inhibitors: E-64, a covalent and irreversible PLCP inhibitor (Hanada et al., 1978; Barrett et al., 1982), CC9, an endogenous cystatin (van der Linde et al., 2012a) and cMIP, a conserved microbial inhibitor of proteases shown to inhibit maize PLCPs (Misas-Villamil et al., 2019). To test their inhibitory efficiency toward the CP1-like PLCPs, we performed an inhibitor concentration range using a substrate cleavage assay with Z-LR-AMC. Equal amounts of active PLCPs were used based on signal quantification from MV201 labeled apoplastic fluids (Supplementary Figure 3). The cleavage activity of each PLCP in the absence of inhibitors was set to 1 and plotted against Log of inhibitor concentration. E-64 and CC9 show strong inhibition of PLCPs already in the nanomolar-range, with E-64 being a stronger inhibitor for all tested PLCPs than CC9 (Figure 6A,B). On the contrary, micromolar concentrations of cMIP were needed to reach inhibition (Figure 6C). CP1C is most susceptible toward E-64 compared to CP1A and CP1B (Figure 6A) and shows a tendency to be less susceptible toward CC9 (Figure 6B). Strikingly, cMIP is least effective for CP1B and most effective for CP1A inhibition. CP1C shows an intermediate susceptibility toward cMIP and at lower inhibitor concentrations, between 30 and 250 nM, CP1C activity seems to be enhanced. On the contrary, CP1A and CP1B show a gradually, dose-dependent reduction in activity with increasing cMIP concentration (Figure 6C).

In this work we found that apoplastic PLCP expression and activity differ in leaf and root proteomes and that PLCP activity after SA treatment is likely a posttranslational process. We identified three different SA-activated PLCPs in roots, not detected in leaves, suggesting a divergent mechanism of SA signaling through distinct PLCPs in different organs. Additionally, we identified CP1C, a root specific CP1-like PLCP of the RD21 subfamily. CP1C shows structure and sequence similarities to CP1A but displays different substrate specificity and inhibitor susceptibility. Differences at the surface and in close proximity to the catalytic triad might suggest distinct interaction partners.

## Discussion

In this study we characterized changes in protein abundance and activity of RAF of maize seedlings after SA treatment. We specifically focused on the activity of PLCPs which have been described as hubs during plant immunity (Misas-Villamil et al., 2016). We have identified and biochemically characterized CP1C, a novel root specific PLCP sharing sequence homology to the Arabidopsis RD21 subfamily (subfamily 1).

Mass spectrometry analysis of IEC samples identified CP1C mostly present in peak 1 whereas CP1A and CP1B appeared in the second and third peak. The differences in fractionation can be attributed to differential charges. CP1C has a higher isoelectric point (pI: 5.55, without signal peptide and prodomain) compared to CP1A and CP1B (pI: 5.09 and 5.10, without signal peptide and prodomain, respectively). A lower pI corresponds to a stronger negative charge at a specific pH. We have performed IEC at pH 6 which results in a stronger binding to the resin of proteins with lower pI and elution at higher salt concentrations. The differences in elution of CP1A, CP1B, and CP1C are therefore in line with their respective pI of the mature protein. Comparing PLCP activity of root apoplastic fluid of SA-treated and mock-treated plants, we detected an overall increased activity after SA treatment and also a shift to lower molecular weight (MW) signals (Figure 2A). The size shift might be caused by an inactivation of higher MW PLCPs, like CP1C (Figure 1C,D) and an activation of other PLCPs with lower MW. Because CP1C was not differentially activated after SA treatment one could assume that this protease is not involved in root SA signaling.

Analysis of the protein abundance after SA treatment showed an increase of proteins associated with SA-signaling like thioredoxins (Tada et al., 2008), shikimate biosynthesis protein (Dempsey et al., 2011) and pathogenesis related PR10 (Chen et al., 2010), confirming the success of the treatment. We also noticed that the abundance of cysteine protease inhibitors did not change after SA treatment except for the cystatin P31726 (Psei1) suggesting a role in closer PLCP regulation after SA treatment (Turk and Bode, 1991). In contrast to CC9, which is highly expressed in leaves, the cystatin P31726 shows sequence similarity to AtCys1, an Arabidopsis cystatin found to be involved in cell death inhibition (Belenghi et al., 2003).

All six apoplastic PLCPs previously identified did not change in abundance upon SA treatment, indicating that the higher activity observed could be due to a posttranslational activation rather than transcriptional regulation. PLCPs can be posttranslational activated through pH shifts, e.g., after translocation into the apoplast (Felle, 1998; Kosegarten et al., 1999; Feliciangeli et al., 2006; Schröder et al., 2010). Posttranslational activation of PLCPs occurs through cleavage of the prodomain from the protease domain (Bryan, 2002). Using DCG-04 labeling we could selectively pull down active PLCPs and compare their activity to their abundance after SA treatment. Surprisingly, we did not observe any of the six previously identified PLCPs, also present in leaves besides CP1C, being more active after SA treatment. Due to the high microbial interaction pressure on roots (Young and Crawford, 2004) compared to aerial parts (Andrews and Harris, 2000; Lindow and Brandl, 2003) the root specific CP1C might be involved in a in a different biological process not directly related to SA signaling. CP1C could be involved in processes such as senescence and apoptosis during the root-development described for other PLCPs such as SAG12 (Lohman et al., 1994; Noh and Amasino, 1999b; Otegui et al., 2005). It could also play a role in nitrogen uptake as shown for other root PLCPs (Godlewski and Adamczyk, 2007; Rentsch et al., 2007; Paungfoo-Lonhienne et al., 2009).

Interestingly, we have identified three PLCPs (B4FS65, B4FYA3, and Q10716) activated after SA treatment. We did not see changes in their abundance indicating that these PLCPs are likely activated via posttranslational modifications. B4FS65 belongs to the THI1 subfamily in which a representative member is the cysteine protease 51 (CP51), an anther-specific cysteine protease, essential for pollen exine formation in *A. thaliana* and potentially involved in PCD (Yang et al., 2014). Q10716 belongs to the Arabidopsis RD19A subfamily of which members are known to be involved in *A. thaliana* defense mechanisms such as RD19 that is targeted by the *Ralstonia* effector PopP2 (Bernoux et al., 2008). B4FYA3 shares high sequence similarity with CP14, containing a granulin domain and belonging to the XBCP3 family. CP14 was described to be involved in programed cell death during plant development (Paireder et al., 2016) where its homolog in *N. benthamiana* NbCP14 was shown to contribute to defense against *Phytophtora infestans* (Kaschani et al., 2010; Bozkurt et al., 2011). Taking together these results show that the leaf characterized PLCPs, CP1A, CP1B, CathB, CP2, and XCP2 as well as the new root PLCP CP1C, might not contribute to the increased PLCP activity after SA treatment in maize roots suggesting that different PLCPs contribute to SA signaling in roots and leaves.

Comparison of maize leaf and root apoplastic PLCPs identified CP1C, a root specific PLCP. Strikingly, CP1C transcripts can be found in leaves. The fact that CP1C has not been previously identified in leaf apoplastic fluids could be explained by its low expression and/or poor activity. Phylogenetic analysis of all maize PLCPs revealed that CP1C groups into the Arabidopsis RD21 subfamily together with previously identified apoplastic PLCPs like CP1A, CP1B and Mir1-3. Due to its high sequence and structural similarities toward CP1A and CP1B it was named CP1C, although CP1C is closer to other maize PLCPs such as Mir1, Mir2 and a pseudotzain whereas CP1A and CP1B are closer to Mir3. The active site of CP1C appears to be narrower than that of CP1A. These structural and biochemical changes might explain the differences in substrate specificity and inhibitor susceptibility in comparison to the other CP1-like PLCPs. The observation that the synthetic substrate AMC-FR, carrying a bulky Phe at the P2-position is preferred by CP1A than by CP1C might also correlate to the narrow grove around the active site in CP1C. Based on the literature, hydrophobic amino acids as well as Arg are predicted to be favored by PLCPs (Niemer et al., 2016; Paireder et al., 2017). We therefore tested three synthetic substrates with different P2-positions. Differential affinities toward the different substrates were observed for CP1-like proteins, which may be explained by unequal substrate accessibility to their active site. Interestingly, the affinities to the tested substrates of CP1C are similar to those of XCP2 and CathB which belong to different subfamilies of PLCPs (subfamily 3 and 9, respectively), likely reflecting similar target preferences *in vivo*. Moreover, we did not observe striking inhibitory differences between the CP1-like PLCPs toward CC9, an endogenous cystatin mostly induced in leaves (van der Linde et al., 2012a). It would be interesting to compare the susceptibility of CP1-like proteases against the cystatin P31726 found to be induced after SA treatment. Interestingly, CP1C seems to be more susceptible toward E-64, an inhibitor produced and first isolated from soil *Aspergillus japonicus* (Hanada et al., 1978).

In this study we discovered a novel root specific PLCP that shows sequence and structure similarity to CP1A but differs in substrate specificity. Surprisingly, neither CP1C nor any other of our six previously found apoplastic PLCPs shows higher activity after SA treatment of maize roots. On the contrary, we have identified three additional root apoplastic PLCPs activated after SA treatment indicating a role in SA-signaling and plant immunity. Both, the further characterization of CP1C to elucidate its specific role in the root apoplast and the functional characterization of the three new SA-induced apoplastic PLCPs will provide us with a deeper understanding of the diverse roles of PLCPs in the root apoplast.

## Materials and Methods

### Plant Material

*Zea mays* variety EGB was grown in phyto-chambers at 28°C on a long day period (16 h light) with 80% humidity. Temperature was decreased to 22°C for 8 h during the night.

*Nicotiana benthamiana* plants were grown in a greenhouse at 23°C on a long day (16 h light) and at 20°C for 8 h dark period with 30– 40% humidity.

### Salicilic Acid (SA) Treatment of Maize Roots and Leaves

Maize plants were sowed in Seramis clay granulat (Sermanis GmbH, Mogendorf, Germany) for root treatment and in soil for leaf treatment and grown for 7–10 days until the three leaf stage. Afterward, 2 mM SA was dissolved in 0.1% ethanol and poured to the maize roots every 12 h for 2 days. As a control mock treated plants were poured with a solution containing 0.1% ethanol. Plants were harvested 60 h after treatment. For leaf treatment same solution were infiltrated into the third leaf using a 1 ml tuberculin-syringe without a hypodermic needle and harvested after 48 h.

### Apoplastic Fluid Isolation

For RAF maize plants grown in Seramis were carefully removed from the pots and Seramis clay granulat was removed from the roots using forceps and washes with ddH2O. Roots were separated from the aerial plant parts and put into a beaker filled with ddH2O. A metal-sieve was added on top to prevent roots from swimming out of the ddH2O. Roots were then vacuum infiltrated 3 times for 15 min at 60 mbar with an interval of 2 min atmospheric pressure. Roots were transferred to syringes hanging in 50 ml falcon tubes and centrifuged at 4°C for 20 min at 3000 g to isolate the apoplastic fluid. Prior to storage at -20°C or direct use in experiments the fluid was passed through a 45 μm syringe filter. Fractionation of apoplastic fluid was performed according to van der Linde et al. (2012a). Apoplastic fluid from maize leaves was prepared as described above except that the leaves were centrifuged at 4°C for 20 min at 2000 g.

### Isolation of Apoplastic Fluids From *N. benthamiana* Leaves

Isolation of apoplastic fluids from *N. benthamiana* leaves was performed as described before in Mueller et al. (2013).

### Protease Activity Assay Using Fluorogenic Substrates

Root apoplastic fluids as well as apoplstic fluids containing overexpressed PLCPs were tested for its activity using the following substrates: Z-FR-AMC, BZ-FVR-AMC, Z-LR-AMC, Z-RR-AMC (Sigma-Aldrich, St. Louis, MS, United States). For sample measurement 10 μl of apoplastic fluids were mixed with reaction buffer (10 mM sodium phosphate pH 6, 150 mM sodium chloride, 1 mM EDTA and 0.5 mM DTT) and 10 μM substrate. AMC-release was measured over time for 20 min (Excitation: 350 nm, Emission: 460 nm) using a Tecan Infinite 200 Pro plate reader (Tecan Group Ltd., Männendorf, Switzerland). As a control for PLCP activity 2 μM E-64 (Sigma-Aldrich, St. Louis, MS, United States) was added to normalize values. cMIP was obtained as synthetic peptide from GenScript (NJ, United States) and diluted in ddH2O to the needed concentration. CC9 was produced and purified according to van der Linde et al. (2012b). Inhibitors were used as described in the results section and added to the indicated concentrations in the experiments ranging from 15 pM to 128 μM. Relative PLCP activity was calculated to the measured activity without addition of inhibitors.

### Activity Based Protein Profiling (ABPP)

Root apoplastic fluid was incubated for 2 h in 50 mM sodium acetate pH 6, 10 mM DTT and 0.2–2 μM of the probe MV201 or DCG-04, respectively (Greenbaum et al., 2000; Richau et al., 2012). As a negative control, one set of samples was pre-incubated for 30 min with 20 μM E-64 (Sigma-Aldrich, St. Louis, MS, United States) prior to labeling. MV201 labeling was performed in darkness. Labeling was stopped by addition of 1xSDS-loading dye (Laemmli, 1970). Samples were heated to 95°C for 5 min and proteins were separated on 12% SDS-gels. For MV201 labeled samples SDS-PAGE was performed in darkness and visualized on gel fluorescent scanning using a ChemiDoc (Biorad, CA, United States) with Rhodamine settings (excitation: 532 nm, emission: 580 nm). The loading control gel was stained with SyproRuby (Invitrogen, Carlsbad, CA, United States) according to the protocol by the manufacturer. Detection of DCG-04 labeled samples was performed using a streptavidin-HRP antibody (Sigma-Aldrich, St. Louis, MS, United States).

### PLCP Pulldown Using Streptavidin-Beads

Root apoplastic fluid was incubated for 4 h at room temperature in 50 mM sodium acetate pH 6, 10 mM DTT and 2 μM DCG-04 (Greenbaum et al., 2000) in a total volume of 2.5 ml. As a negative control, one set of samples was pre-incubated for 30 min with 20 μM E-64 (Sigma-Aldrich, St. Louis, MS, United States) prior to labeling. After labeling, samples were transferred and eluted using NaP25 columns (GE healthcare, Chicago, IL, United States) equilibrated with 50 mM Tris-HCl pH 8. Hundred microliter streptavidin sepharose high performance (Sigma-Aldrich, St. Louis, MS, United States), equilibrated with 50 mM Tris-HCl pH 8 and 1 tablet inhibitor cocktail mix (complete^TM^, EDTA-free Protease Inhibitor Cocktail, Roche, Basel, Switzerland) was mixed with the sample and incubated for 1 h at room temperature rotating. Samples were centrifuged for 3 min at 1,400 g and the supernatant was discarded. Sepharose beads were gently re-suspended in 1 ml 50 mM Tris-HCl pH 8 in a new tube. The sepharose beads were washed two times with 1% SDS and two times with 6M Urea. Beads were once washed with 1 ml 50 mM Tris-HCl pH 8 containing 0.1% Tween20 and once with ddH2O. Beads were stored at -20°C until further analysis. Control samples were taken after each step. To confirm the pulldown assay an immunoblot with control samples was performed using streptavidin-HRP antibody (1 μg/ml) (Sigma-Aldrich, St. Louis, MS, United States). The immunoblot was developed using SuperSignal^TM^ West Pico Chemiluminescent Substrate (Thermo Fischer Scientific, Waltham, MA, United States).

### Sample Preparation for LC/MS/MS

Samples for LC-MS from proteins labeled with DCG-04 and enriched on streptavidin beads were either prepared by gel electrophoresis and subsequent in-gel digestion (IGD) or the captured proteins were directly digested on the beads (OBD). To identify and cut out gel regions containing DCG-04 targets we employed the “blind-cut”-method (van der Linde et al., 2012a). IGD with trypsin was performed by following a published protocol (Kaschani et al., 2009). Affinity enriched protein samples that were not eluted from the capture resin were on-bead digested (OBD). Briefly, streptavidin beads were washed twice with water to remove SDS. Then bound proteins were reduced with DTT (5 mM) in 50 mM ammonium bicarbonate (ABC) for 30 min at room temperature. Protein reduction was followed by alkylation with iodoacetamide (IAM, 10 mM also in 50 mM ABC, 30 min, room temperature) and quenching of excess IAM with DTT (final concentration DTT 10 mM). Reduction and alkylation was followed by a sequential digestion of proteins with first LysC for 3 h at 37°C followed by a 16 h digestion with trypsin (37°C). The digestion was stopped by adding formic acid (FA) to a final concentration of 0.5%. The supernatant containing the digestion products was passed through home-made glass microfiber StageTips (GE Healthcare; poresize: 1.2 μM; thickness: 0.26 mm). Cleared tryptic digests were desalted on home-made C18 StageTips as described (Rappsilber et al., 2007). Peptides were passed over a 2 disc StageTip. After elution from the StageTips, samples were dried using a vacuum concentrator (Eppendorf) and the peptides were taken up in 0.1% FA solution (10 μl).

### LC/MS/MS

Experiments were performed on an Orbitrap Elite instrument (Thermo Fischer Scientific, Waltham, MA, United States; Michalski et al., 2012) that was coupled to an EASY-nLC 1000 liquid chromatography (LC) system (Thermo Fischer Scientific, Waltham, MA, United States). The LC was operated in the one-column mode. The analytical column was a fused silica capillary (inner diameter 75 μm × 35 cm) with an integrated PicoFrit emitter (New Objective, Woburn, United States) packed in-house with Reprosil-Pur 120 C18-AQ 1.9 μm. The analytical column was encased by a column oven (Sonation, Biberach an der Riß, Germany) and attached to a nanospray flex ion source (Thermo Fischer Scientific, Waltham, MA, United States). The column oven temperature was adjusted to 45°C during data acquisition. The LC was equipped with two mobile phases: solvent A (0.1% formic acid, FA, in water) and solvent B (0.1% FA in acetonitrile, ACN). All solvents were of UHPLC (ultra-high performance LC) grade (Sigma-Aldrich, St. Louis, MS, United States). Peptides were directly loaded onto the analytical column with a maximum flow rate that would not exceed the set pressure limit of 980 bar (usually around 0.5–0.8 μl/min). Peptides were subsequently separated on the analytical column by running a 40 min (ISD) or 140 min (OBD) gradient of solvent A and solvent B [start with 7% B; gradient 7–35% B for 30 min (ISD) or 120 min (OBD); gradient 35–100% B for 5 min (ISD) or 10 min (OBD) and 100% B for 5 min (ISD) or 10 min (OBD)] at a flow rate of 300 nl/min. The mass spectrometer was operated using Xcalibur software (version 2.2 SP1.48). The mass spectrometer was set in the positive ion mode. Precursor ion scanning was performed in the Orbitrap analyzer (FTMS; Fourier Transform Mass Spectrometry) in the scan range of m/z 300–1800 and at a resolution of 60,000 with the internal lock mass option turned on (lock mass was 445.120025 m/z, polysiloxane) (Olsen et al., 2005). Product ion spectra were recorded in a data dependent fashion in the ion trap (ITMS; Ion Trap Mass Spectrometry) in a variable scan range and at a rapid scan rate. The ionization potential (spray voltage) was set to 1.8 kV. Peptides were analyzed using a repeating cycle consisting of a full precursor ion scan [1.0 × 10^6^ ions or 200 ms (IGD) and 3.0 × 10^6^ ions or 50 ms) followed by 10 product ion scans (3.0 × 10^4^ ions or 150 ms (IGD) and 1.0 × 10^4^ ions or 50 ms (OBD)] where peptides are isolated based on their intensity in the full survey scan (threshold of 500 counts) for tandem mass spectrum (MS2) generation that permits peptide sequencing and identification. CID (collision-induced dissociation) collision energy was set to 35% for the generation of MS2 spectra. During MS2 data acquisition dynamic ion exclusion was set to 120 s with a maximum list of excluded ions consisting of 500 members and a repeat count of one. Ion injection time prediction, preview mode for the FTMS, monoisotopic precursor selection and charge state screening were enabled. Only charge states higher than 1 were considered for fragmentation.

### Peptide and Protein Identification Using MaxQuant

RAW spectra were submitted to an Andromeda (Cox et al., 2011) search in MaxQuant (version 1.5.3.30) using the default settings (Cox and Mann, 2008). Label-free quantification and match-between-runs was activated (Cox et al., 2014). MS/MS spectra data were searched against the Uniprot *Zea mays* cv B73 database UP000007305_4577.fasta (99369 entries, downloaded 6/4/2018) and the in-house ACE_0229_EGB apoplastic PLCPs AS.fasta database containing Sequences of interest from *Zea mays* cv EGB (7 entries). All searches included a contaminants database (as implemented in MaxQuant, 245 sequences). The contaminants database contains known MS contaminants and was included to estimate the level of contamination. Enzyme specificity was set to “Trypsin/P.” The instrument type in Andromeda searches was set to Orbitrap and the precursor mass tolerance was set to ±20 ppm (first search) and ±4.5 ppm (main search). The MS/MS match tolerance was set to ±0.5 Da. The peptide spectrum matches FDR and the protein FDR were set to 0.01 (based on target-decoy approach and decoy mode “revert”). Minimum peptide length was 7 amino acids. Label-free protein quantification was switched on, and unique and razor peptides were considered for quantification with a minimum ratio count of 2. Retention times were recalibrated based on the built-in nonlinear time-rescaling algorithm. MS/MS identifications were transferred between LC-MS/MS runs with the “Match between runs” option in which the maximal match time window was set to 0.7 min and the alignment time window set to 20 min. The quantification is based on the “value at maximum” of the extracted ion current. Modified peptides were allowed for quantification. The minimum score for modified peptides was 40. Further analysis and filtering of the results was done in Perseus v1.5.5.3 (Tyanova et al., 2016). The mass spectrometry proteomics data have been deposited to the ProteomeXchange Consortium via the PRIDE (Vizcaino et al., 2016) partner repository^1^ with the dataset identifier PXD013124.

### Strain and Plasmid Construction

Golden gate modular cloning system was applied to generate plasmids (Engler et al., 2014). Oligonucleotides that were used for PCR are listed in Supplementary Table 3. To obtain pL1M-F1-XCP2-Streptwin::2x35S, pL1M-F1-CathB-Streptwin::2x35S, pL1M-F1-CCP2-Streptwin::2x35S, XCP2 (Maizegdb: GRMZM2G066326), Cathepsin B (Maizegdb: GRMZM2G108849), and CP2 (Maizegdb: GRMZM2G038636) respectively were amplified by PCR from maize cDNA. To obtain pL1M-F1- CP1A_nogran-Streptwin::2x35S, pL1M-F1-CP1B_nogran-Streptwin::2x35S, pL1M-F1-CP1C_nongran-HA::2x35S, CP1A (Maizegdb: GRMZM2G166281), CP1B (Maizegdb: GRMZM2G073465), and CP1C (Maizegdb: GRMZM2G340065) respectively were amplified by PCR from maize cDNA leaving out the DNA sequence coding for the granulin-domains. The amplified sequences were then ligated according to Weber et al. (2011) and Engler et al. (2014), sub-transformed to *E. coli* DH5α competent cells (Thermo Fischer Scientific, Rockfort, United States) and then transformed to *A. tumefaciens* GV3101 competent cells for overexpression in *N. benthamiana*. To obtain pL1M-F1-CP1A_nogran_mut2-Streptwin::2x35S site directed mutagenesis was performed on pL1M-F1- CP1A_nogran-Streptwin::2x35S according to the instructions of the QuikChange Multi Site-Directed Mutagenesis Kit (Agilent Technologies, Santa Clara, United States) with primers targeting nucleotides of the active site of CP1A. Strains used in this study are listed in Supplementary Table 4.

### Heterologous Expression of PLCPs in *N. benthamiana* Leaves

*Agrobacterium tumefaciens* containing the desired constructs were grown in liquid media overnight and diluted in 10 mM magnesium chloride to an OD = 1 with 200 μM acetosyringone (Sigma-Aldrich, Taufkirchen, Germany). After 1 h incubation in the dark cultures were infiltrated into 5–6 weeks old N. benthamiana leaves using a tuberculin-syringe without needle. Three days postinfiltration leaves were harvested and the apoplastic fluid was isolated.

### Computational Methods and Statistical Analysis

Heat-maps were performed using the heatmap.2 function of the package gplots (version 3.0.1) in r-studio (R version 3.5.1). Venn diagram was created using the draw.pairwise.venn function of the package Venn diagram (version 1.6.0) in r-studio (R version 3.5.1.). For generation of a phylogenetic tree 52 maize PLCP sequences of the line B73 retrieved from the MEROPS database (Rawlings et al., 2018) and our six identified PLCPs from EGB were used. Additionally, we included one type member of each PLCP subfamily of *A. thaliana* and two serine proteases DEGP2 and DGP11 from *A. thaliana* as outgroup (Beers et al., 2004; Richau et al., 2012). MAFFT (v7.407) (Katoh and Standley, 2013). RAxML with the GTRGAMMA substitution model (v8.2.0) was used for the construction of the tree (Stamatakis, 2014). The tree is drawn to scale, with branch lengths measured in the number of substitutions per site. The robustness was assessed using 100 bootstrap replicates. Quantification of PLCP-signals after ABPP using rhodamine fluorescence signal strength was performed using ImageLab^TM^ software (Bio-Rad, Hercules, CA, United States). Phyre2 (Kelley et al., 2015) was used for modeling of PLCPs based on caricain PDB: 1pciA (Groves et al., 1996). For the inhibitor concentration range plots a nonlinear fit based on the dose response function and calculation of IC50 was performed in Origin 2018 (OriginLab, Northampton, MA, United States).

## Author Contributions

JS wrote the manuscript with input from all authors. JS, KvdL, GD, and JM designed the experiments. AM, SZ, and KvdL performed IEC in leaves and roots. KvdL and FK did the DCG-04 pull-down of IEC samples. FK, MK, and TC performed MS/MS analysis and protein identification.

## Conflict of Interest Statement

The authors declare that the research was conducted in the absence of any commercial or financial relationships that could be construed as a potential conflict of interest.

## Footnotes

1 https://www.ebi.ac.uk/pride/archive/
