# Supplementary material for: Proteases Underground: Analysis of the Maize Root Apoplast Identifies Organ Specific Papain-Like Cysteine Protease Activity
Source: Front Plant Sci. 2019 Apr 30;10:473. doi: 10.3389/fpls.2019.00473 (PMC6503450; doi:10.3389/fpls.2019.00473)
Supplement: Supplementary file 5 [file Table_5.docx]

**Table 5**: Maize PLCP alignment including *A. thaliana* type members and outgroup

>MER0001404

------------------------------------------------------------

------------------------------------------------------------

----------------------------------MAH-----------------------

------------------------------------------------------------

-----------------------------RVLLLLSLASA--------------------

-------------------------------AAVAAAVDA--------------------

------------------------------------------------------------

--EDPLIRQVVP------------GGDD--------NDL---------------------

-----------------------------ELNAESHFLSFVQRF----------------

-------GK--------------------------------------------SY-----

-------------------------KDA-------------------------------D

EHAY--------------------------------------------------------

-----------RLSVFKDN-----------------------------------------

------------------------------------------------------------

------------------------------LRRARRHQ---------L-LD----P----

------------------------------------------------SA----------

------------------------------------------------------------

---------------------------EHG--------------------------VTKF

SDLTPAEFRRTYL----GLRK---SRRAL---------LRELGESAHEAPV---------

-----------------------------------LPTD-GLPDD---------------

---------------FD----WRDHGAVG-------------------PVKNQG------

----SCGSCWSFSASGALEGAHYL-AT----------------GKLEVLSEQQFVDC---

------DHECDSSEPD----------------SCDSGCNGG---------LMTTAFS---

-----------YLQKA-----G-GLESEKD----------------Y-PYTGSDGK----

---------CKFDK---------------SKIVAS----------------VQNFSVVSV

DEAQI--------------SANLIK-HGPLAIGINAA--YMQTYIG--------------

-----------------------GVSCP-----------------------------YIC

G-R-----H-LDH-----------------------------------------------

-----------------------------GVLLVG-----------YGASGFAP------

-----------IRLKDKPY---------------W------IIKNSWGEN----------

WG-----------------------ENGYYK--ICRGSNV--------------------

-------------RNKCG-------------------------------VDSM-------

-------------VSTV---------SAVH--------------------ASKE------

------------------------------------------------------------

------------------------------------------------------------

------------------------------------------------------------

------------------------------------------------------------

------------------------------------------------------------

------------------------------------------------------------

------------------------------------------------------------

------------------------------------------------------------

>MER0144283

------------------------------------------------------------

--------MEFEETPADESS-----------LNLRRRRPRRTSRGRQPKSP-----AQAA

NLAQRT--------------------------CRMAQ-----------------------

-----------------------VSKTL--------------------------------

-----------------------------LLVALVFVSSA--------------------

-------------------------------AVEL-------------------------

--------------------------------------------CR-AIDFDER------

------------------------DLAS--------DE----------------------

-------------------------------ALWDLYERWQTHH----------------

--------R--------------------------------------------VH-----

-------------------------RHH-------------------------------G

EKGR--------------------------------------------------------

-----------RFGTFKEN-----------------------------------------

------------------------------------------------------------

------------------------------VRFIHAHN---------K-RG---DR----

------------------------------------------------PY----------

------------------------------------------------------------

---------------------------RLR--------------------------LNRF

GDMGREEFRSTFA----DSRI---NDLR-------------RQDSPAAR-----------

------------AGAVPG----FMY----------DSAA-DPPRS---------------

---------------VD----WRQEGAVT-------------------GVKDQG------

----HCGSCWAFSTVVAVEGINAI-RT----------------GSLASLSEQELIDC---

------DTD-------------------------ENGCQGG---------LMENAFE---

-----------FIKSF-----G-GITTEAA----------------Y-PYRASNGT----

---------CDGDRARR-----------GGGVVVV----------------IDGHQMVPA

GSEDA--------------LAKAVA-HQPVSVAVDAGGQAFQFYSE--------------

-----------------------GVFTG------------------------------DC

G-T-----D-LDH-----------------------------------------------

-----------------------------GVAAVG-----------YGVGD---------

--------------DGTPY---------------W------IVKNSWGTS----------

WG-----------------------EGGYIR--MQRGAGN--------------------

-------------GGLCG-------------------------------IAME-------

-------------ASFP---------IKT---------------------SPNP------

--------ADP-P-------------------------------RKPRRA----------

------------------------------------------------------------

------------------------------------------------------------

------------------------------------------------------------

-------------------------------LIARDTSSSQ-------------------

------------------------------------------------------------

------------------------------------------------------------

------------------------------------------------------------

>MER0361006

------------------------------------------------------------

------------------------------------------------------------

----------------------------------MAQ-----------------------

-----------------------VAKTL--------------------------------

-----------------------------LLVALVVVS----------------------

-------------------------------AVEL-------------------------

--------------------------------------------CR-AIEFDER------

------------------------DLAS--------DE----------------------

-------------------------------ALWDLYERWQTHH----------------

--------R--------------------------------------------VH-----

-------------------------RHH-------------------------------G

EKGR--------------------------------------------------------

-----------RFGTFKEN-----------------------------------------

------------------------------------------------------------

------------------------------ARFIHAHN---------K-RG---DR----

------------------------------------------------PY----------

------------------------------------------------------------

---------------------------RLR--------------------------LNRF

GDMGREEFRSGFA----DSRI---NDLR-------------REPTAAPA-----------

---------------VPG----FMY----------DDAT-DLPRS---------------

---------------VD----WRQKGAVT-------------------AVKNQG------

----RCGSCWAFSTVVAVEGINAI-RT----------------GSLVSLSEQELIDC---

------DTD-------------------------ENGCQGG---------LMENAFE---

-----------FIKSH-----G-GITTESA----------------Y-PYHASNGT----

---------CDGARAR-------------RGRVVA----------------IDGHQAVPA

GSEDA--------------LAKAVA-HQPVSVAIDAGGQALQFYSE--------------

-----------------------GVFTG------------------------------DC

G-T-----D-LDH-----------------------------------------------

-----------------------------GVAAVG-----------YGVSD---------

--------------DGTPY---------------W------IVKNSWGPS----------

WG-----------------------EGGYIR--MQRGTGN--------------------

-------------GGLCG-------------------------------IAME-------

-------------ASFP---------IKT---------------------SPNP------

--------S-----------------------------------RKPRRA----------

------------------------------------------------------------

------------------------------------------------------------

------------------------------------------------------------

-------------------------------LITRDASSQ--------------------

------------------------------------------------------------

------------------------------------------------------------

------------------------------------------------------------

>MER0367290

------------------------------------------------------------

------------------------------------------------------------

----------------------------------MAR-----------------------

-----------------------SRRSL----------------------------PT--

-----------------------------KVTMGSTTMAT--------------------

----------------------------RSRSSSQPKF------GASPHRG---------

--------------TTPSPWGTDNV-DSAW------PHRSQTNPAKVALLLMDK------

------------------------DLES--------KE----------------------

----------------------------------SMYERWSSVH----------------

-------TV--------------------------------------------S------

-------------------------RDL-------------------------------T

EKQS--------------------------------------------------------

-----------RFEAFKAN-----------------------------------------

------------------------------------------------------------

------------------------------SRHIGEFN---------K-MK---DV----

------------------------------------------------PY----------

------------------------------------------------------------

---------------------------KLG--------------------------LNKF

ADLTREEFVSKYT----GAKVVDPDSEA-------------AATAARLASG---------

----------------------VRV----------LSSD-ELPPQLVASTVNAIITAVKD

HRAVTAVNGVNLFCIAD----WRDHGAIT-------------------AVKDQG------

----QCGSCWAFSAVGA----------N---------------KRKVEKIENDYPKC---

--------------------------------KFIAQC-----FIY----DIDVAYDVLI

TIQSI----VKYVVVL--LDKS-GEPNGKI----------------Y-TWDATFLR----

---------KRLGYS--------------AHKLLS---V-----------------DFPS

EDAGKG-------------WQSKIV-KQLVHCSLLVK-IAFLFNRNADRRNTEGSNQYPY

VSPS------GS-----------GSAAG------------AVACI-------------AC

P-T---LYAYLKK---------------------------------------TDP-GV--

-----------------------------PAQLLEY----DERFGQYGCDFTFYDYNRPE

ELPAAMKHAYRVIVADPPY----------------------LSKECLEKFAKAVSFLARP

EGSFLLL------------------LTGEVQ--MDRALEL--------------------

----------LN-VRPCGFR----------------------------------------

-------------PRHS-------NKLGNEFRLFT--------------N----------

------------------------------------------------------------

------------------------------------------------------------

------------------------------------------------------------

------------------------------------------------------------

------------------------------------------------------------

------------------------------------------------------------

------------------------------------------------------------

------------------------------------------------------------

>MER0228306

------------------------------------------------------------

------------------------------------------------------------

----------------------------------MGA-----------------------

----------------------STTPLASAAA----------------------------

-----------------------------LLLLLLAPLAA--------------------

-------------------------------AADS-------------------------

--------------------------------------------MS-IVSY---------

------------------------GERS--------EE----------------------

-------------------------------EARRMYAEWMAAH----------------

-------GR--------------------------------------------TY-----

-------------------------NAV-------------------------------G

EEER--------------------------------------------------------

-----------RFEVFRDN-----------------------------------------

------------------------------------------------------------

------------------------------LRYVDAHN---------A-AADAGVH----

------------------------------------------------SF----------

------------------------------------------------------------

---------------------------RLG--------------------------LNRF

ADLTNDEYRATYL----GVRS---RPQR-------------ERR----------------

----------------LG----DRY-L----A---GDNE-DLPES---------------

---------------VD----WRAKGAVA-------------------EVKDQG------

----SCGSCWAFSTIAAVEGINQI-VT----------------GDMISLSEQELVDC---

------DT------------------------SYNQGCNGG---------LMDYAFE---

-----------FIINN-----G-GIDTEED----------------Y-PYKGTDGR----

---------CDVNRK--------------NAKVVT----------------IDSYEDVPA

NSEKS--------------LQKAVA-NQPISVAIEAGGRAFQLYNS--------------

-----------------------GIFTG------------------------------TC

G-T-----A-LDH-----------------------------------------------

-----------------------------GVTAVG-----------YG-TE---------

--------------NGKDY---------------W------IVKNSWGSS----------

WG-----------------------ESGYVR--MERNIKA--------------------

-----------S-SGKCG-------------------------------IAVE-------

-------------PSYP---------LKK---------------------GANP------

--------PNPGP-------------------------------TPPSPTP---------

------------------------------------------------------------

---------------------------------PPT------------------------

------------------------------------VCDNYY------------------

--------------------------------SCPDSTTCC-------------------

---------------------------------------------CIYEYGKYCFAWG-C

CPLEGATCCD----DHYSCCPHDYPVCNVKQGTCLMGKDSPLSLS-----VKATKRTLAK

PHWAFPGNTA---------------ADGMKSSA---------------------------

>MER0136741

------------------------------------------------------------

------------------------------------------------------------

----------------------------------MGPINLTACRAPESTHRPNRKNRESK

RKEQGGGEKRRAKKGKGRTSRHVLHRSTPAVM------------AALG------------

-----------------------------LLLLLLLAVVG--------------------

-------------------------------AANAAA-----------------------

---------------------------------------PGGR-MS-IISYNEEHA----

---------ARG------------LERT--------EP----------------------

-------------------------------EARTLYELWLAEH----------------

-------GR--------------------------------------------AY-----

-------------------------NAL-------------------------------G

ERDR--------------------------------------------------------

-----------RFRVFWDN-----------------------------------------

------------------------------------------------------------

------------------------------LRFVDAHN---------E-RA--AEH----

------------------------------------------------GF----------

------------------------------------------------------------

---------------------------RLG--------------------------MNQF

ADLTNDEFRAAYL----GARI---PASR-------------RRGT---------------

---------------AVG----ERYRH----G---GGAE-ELPES---------------

---------------VD----WREKGAVA-------------------PVKNQG------

----QCGSCWAFSAVSSVESVNQI-VT----------------GEMVTLSEQELVEC---

------STD-----------------------GGNSGCNGG---------LMDAAFD---

-----------FIIKN-----G-GIDTEGD----------------Y-PYKAVDGK----

---------CDINRE--------------NAKVVS----------------IDGFEDVPE

NDEKS--------------LQKAVA-HQPVSVAIEAGGREFQLYKA--------------

-----------------------GVFTG------------------------------TC

T-T-----N-LDH-----------------------------------------------

-----------------------------GVVAVG-----------YG-TE---------

--------------NGKDY---------------W------IVRNSWGAK----------

WG-----------------------EDGYIR--MERNVNA--------------------

-----------T-TGKCG-------------------------------IAMM-------

-------------ASYP---------TKK---------------------GANP------

--------PKPSP-------------------------------TPPTPPP---------

------------------------------------------------------------

---------------------------------PPVAPDN--------------------

------------------------------------VCDENF------------------

--------------------------------SCAAGSTCC-------------------

---------------------------------------------CAFGFRNVCLVWG-C

CPMEGATCCK----DHASCCPPGYPVCNVRAGTCSVSKNSPLS-------VKALKRTLAK

LNSA--------------------------------------------------------

>MER0363913

------------------------------------------------------------

------------------------------------------------------------

----------------------------------MAM-----------------------

------------------------------------------------------------

------------------------------------------------------------

------------------------------------------------------------

------------------------------------------------------------

------------------------------------------------------------

------------------------------------------------------------

-------GK---------------------------------------------Y-----

------------------------------------------------------------

------------------------------------------------------------

------------------------------------------------------------

------------------------------------------------------------

------------------------------------------------------------

------------------------------------------------------------

------------------------------------------------------------

------------------------------------------------------------

------------------------------------------------------------

------------------------------------------------------------

------------------------------------------------------------

----PSGSCWAFSTIAAVEGINQI-VT----------------GDLISLSEQELVDC---

------DT------------------------SYNQGCNGG---------LMDYAFE---

-----------FIINN-----G-GIDTEKD----------------Y-PYKGTDGR----

---------CDVNRK--------------NAKVVT----------------IDIYEDVPA

NDEKS--------------LQKAVA-NQPVSVAIEAAGTTFQLYSSVHETRSRVHAA---

------AQEQ-------------GKLAL-----------------KKQKPNNEIQERDQN

GPSKNKK-S-ADNEGQDREPEVPTKKLKAG--------------------R-SEPNGREA

-----------------------------ATRKIAE---------FYKAIG---------

-----------EHLSSREYE--PNVCEGQLEFKEWK---YKCTGNCTGNYTE--------

WAR-CTFRTDDPSRRSGPIKVPDDIKDDFIRKWLKQQEGKGKEFP----KHDVDEEAHIL

SSMM---------IDLSG-Q-----------------------------MTRL-------

-------------HVIP---------LNNL------------------------------

------------------------------------------------------------

------------------------------------------------------------

------------------------------------------------------------

------------------------------------------------------------

------------------------------------------------------------

----------------------------------------------------ICYS----

------------------------------------------------------------

------------------------------------------------------------

>MER0003754

------------------------------------------------------------

------------------------------------------------------------

----------------------------------MAA-----------------------

----------------------STT--AAAAA----------------------------

-----------------------------SVLLLLLSLAR--------------------

-------------------------------GGGL-------------------------

--------------------------------------------MS-IVSY---------

------------------------GERT--------DE----------------------

-------------------------------EARRMYAEWMAAH----------------

-------GR--------------------------------------------TY-----

-------------------------NAV-------------------------------G

AEER--------------------------------------------------------

-----------RYQVFRDN-----------------------------------------

------------------------------------------------------------

------------------------------LRYIDAHN---------A-AADAGVH----

------------------------------------------------SF----------

------------------------------------------------------------

---------------------------RLG--------------------------LNRF

ADLTNDEYPATYL----GART---RPQR-------------DRK----------------

----------------LG----ARY-H----A---ADNE-DLPES---------------

---------------VD----WRAKGAVA-------------------EVKDQG------

----SCGTCWAFSTIAAVEGINQI-VT----------------GDLISLSEQELVDC---

------DT------------------------SYNQGCNGG---------LMDYAFE---

-----------FIINN-----G-GIDTEKD----------------Y-PYKGTDGR----

---------CDVNRK--------------NAKVVT----------------IDSYEDVPA

NDEKS--------------LQKAVA-NQPVSVAIEAAGTAFQLYSS--------------

-----------------------GIFTG------------------------------SC

G-T-----R-LDH-----------------------------------------------

-----------------------------GVTAVG-----------YG-TE---------

--------------NGKDY---------------W------IVKNSWGSS----------

WG-----------------------ESGYVR--MERNIKA--------------------

-----------S-SGKCG-------------------------------IAVE-------

-------------PSYP---------LKE---------------------GANP------

--------PNPGP-------------------------------SPPSPTP---------

------------------------------------------------------------

---------------------------------APA------------------------

------------------------------------VCDNYY------------------

--------------------------------SCPDSTTCC-------------------

---------------------------------------------CIYEYGKYCFAWG-C

CPLEGATCCD----DHYSCCPHDYPICNVRQGTSLMGKDSPLSLS-----VKATKRTLAK

RTGLSPATQ----------------LTALKSSALRKRSVALGRQQPG-------------

>MER0361184

---------------------------------------------MDIVVEKGNVDVGNT

AEPS-YEVVARSGCSAQLSTNDTDEHELESE-DSKVSVGNAAKQSHDVVGVGIDCIFNHV

KVSTEADLGPQPDVIHLTSLDVPNESSQISSDADMVKQ----------------------

----------------------NQQDALDTLIRISQHKKPKKLNVARVVSEDYKCTPEDV

QLIEYIKTLPGKQVVVNIDSAWLNRNDMECLFHGDMQLSD--------------------

-------------------------------KALNAYIHCIRGEEHLLHRE--GGKVFLE

NTFISSL---LKRDGDPKVFLPMNIEDFHWYLAVLNAKKSELKGLERQIKLAAKHKE---

----LYQGKWSN-----------LDVAS----WPVIEKITTQMQT-------------DG

VSC--------------------------GLWMINYMEYWTGSSLSDNVTQDDITMFMFK

LPAILWDSRLNTKKGHQNLDHNVDEDGESSSDVQIIDTPLTSTNTQELMFVLCTYIMGID

NDKYLKKHWIQSTKPYPISLSLQKLKDI--LDVNKPMDTDCFNMAVRMIACNDSL-FLLE

DKYHYMDLQFCSITKFGRDPRLRAKPDINMLAKLLECWPDMEYDVSDCKQGF--------

-IWLSCYQLYARLEWYKVT------------------------LH---------------

------------------------------------------------------------

------------------------------LHFVESKDRRISVA--LAFAGHQEEKCSVP

MHVPLRPWQQQGYGNSDVVSLVDLIQILNHKDCFCIFG---------RSYPERVVLNL--

----------------------LTVDATPRVTVSGAPADADPPSPWQRRS----------

---------------------------LLGP-----------------PTLGRGEKLSEL

QDKTSELYIQAQQFKKQGVKI---RRKT-------------WLQNMKIKLV---------

-----ILGILLLLVIIAS----FKR----------LMLK-QQGMR---------------

---------------TT----WEYPFAVA-------------------------------

------GSCWAFSTIAAVEGINQI-VT----------------GDLISLSEQELVDC---

------DT------------------------SYNQGCNGG---------LMDYAFE---

-----------FIINN-----G-GIDTEKD----------------Y-PYKGTDGR----

---------CDVNRK--------------NAKVVT----------------IDSYEDVPA

NDEKS--------------LQKAVA-NQPVSVAIEAAGTTFQLYSS--------------

-----------------------GIFTG------------------------------SC

G-T-----A-LDH-----------------------------------------------

-----------------------------GVTVVG-----------YG-TE---------

--------------NGKDY---------------W------IMKNSWGSS----------

WG-----------------------ESGYVR--MERNIKA--------------------

-----------S-SGKCG-------------------------------IAVE-------

-------------PSYP---------LKE---------------------GANP------

--------PNPGP-------GARRACI-----------------VRPSIN----------

------------------------------------------------------------

-------------IAAPGL--------------PPSEP--------------RE------

--GNT-------------------------------------------------------

------------------------------GNPAPTPPDCA-------------------

DRAGG------------------------------------------------------S

CPERAAQTAAPE--EPHRSCTHRSSLSN---GLCTMRMLLLLRWD---------RSKDFQ

TTTGSDAGPTCSVTKINLPVYHVYSVQGQR------------------------------

>MER0361063

------------------------------------------------------------

------------------------------------------------------------

----------------------------------MAT-----------------------

------------------------LQASIL------------------------------

-----------------------------AVLSFAF-FCG--------------------

-------------------------------AA---------------------------

--------------------------------------------------LAAR------

------------------------DLNE--------DS----------------------

-------------------------------AMVARHEQWMAQY----------------

-------SR--------------------------------------------VY-----

-------------------------KDA-------------------------------A

EKAR--------------------------------------------------------

-----------RFEVFKAN-----------------------------------------

------------------------------------------------------------

------------------------------VKFIESFN---------T-GG---NR----

------------------------------------------------KF----------

------------------------------------------------------------

---------------------------WLG--------------------------INQF

ADLTNDEFRTTKTNK--GFKP---SLDK-------------VS-----------------

----------------TG----FRY-E----N---VSVD-AIPAT---------------

---------------ID----WRTNGAVT-------------------PIKDQG------

----QCGCCWAFSAVAATEGIVKI-ST----------------GKLISLSEQELVDC---

------DVH-----------------------GEDQGCEGG---------LMDDAFK---

-----------FIIKN-----G-GLTTESN----------------Y-PYTAADGK----

---------CKSG----------------SNSAAN----------------IKGYEDVPT

NDEAA--------------LMKAVA-NQPVSVAVDGGDMTFQFYSG--------------

-----------------------GVMTG------------------------------SC

G-T-----D-LDH-----------------------------------------------

-----------------------------GIAAIG-----------YGKTS---------

--------------DGTKY---------------W------LMKNSWGTT----------

WG-----------------------ENGYLR--MEKDISD--------------------

-----------K-KGMCG-------------------------------LAME-------

-------------PSYP---------TE--------------------------------

------------------------------------------------------------

------------------------------------------------------------

------------------------------------------------------------

------------------------------------------------------------

------------------------------------------------------------

------------------------------------------------------------

------------------------------------------------------------

------------------------------------------------------------

>MER0361050

MNTVFYTTSGKHIYTCFTVAYQSLSPQYSVQAVYRMASYAYNLITKDNTRLKLHINT--R

TTPG--------------SNQEIIRHSAQQQ-------QASISARPIPRGLMQ-------

-------------------------------RSTMAT-----------------------

------------------------LKASIS------------------------------

-----------------------------AIIGFAF-FCG--------------------

-------------------------------AA---------------------------

--------------------------------------------------MAAR------

------------------------DLSD--------DS----------------------

-------------------------------VMVARHEQWMAQY----------------

-------SR--------------------------------------------VY-----

-------------------------KDA-------------------------------S

EKAR--------------------------------------------------------

-----------RFEVFKAN-----------------------------------------

------------------------------------------------------------

------------------------------VQFIESFN---------A-GG---NN----

------------------------------------------------KF----------

------------------------------------------------------------

---------------------------WLG--------------------------VNQF

ADLTNDEFRSTKTNK--GLKS---SNMK-------------IP-----------------

----------------TG----FRY-E----N---VSAD-ALPTT---------------

---------------ID----WRTKGAVT-------------------PIKDQG------

----QCGCCWAFSAVAATEGIVKI-ST----------------GKLVSLAEQELVDC---

------DVH-----------------------GEDQGCEGG---------LMDDAFK---

-----------FIIKN-----G-GLTTESS----------------Y-PYTAADGK----

---------CKSG----------------SNSAAT----------------IKGYEDVPA

NDEAA--------------LMKAVA-NQPVSVAVDGGDMTFQFYSG--------------

-----------------------GVMTG------------------------------SC

G-T-----D-LDH-----------------------------------------------

-----------------------------GIAAIG-----------YGKTS---------

--------------DGTKY---------------W------LMKNSWGTT----------

WG-----------------------ENGYLR--MEKDISD--------------------

-----------K-RGMCG-------------------------------LAME-------

-------------PSYP---------TE--------------------------------

------------------------------------------------------------

------------------------------------------------------------

------------------------------------------------------------

------------------------------------------------------------

------------------------------------------------------------

------------------------------------------------------------

------------------------------------------------------------

------------------------------------------------------------

>MER0360964

------------------------------------------------------------

------------------------------------------------------------

----------------------------------MVS-----------------------

------------------------SRAFLLLL----------------------------

-----------------------------AILTGCACSFP--------------------

------------------------------------------------------------

-----------------------------------------------SPVLAAR------

------------------------ELSD--------DA----------------------

-------------------------------AMAERHERWMAVY----------------

-------GR--------------------------------------------VY-----

-------------------------KDA-------------------------------A

EKAR--------------------------------------------------------

-----------RFEVFKDN-----------------------------------------

------------------------------------------------------------

------------------------------LAFVESFN---------A-DK---KN----

------------------------------------------------KF----------

------------------------------------------------------------

---------------------------WLG--------------------------VNQF

ADLTTEEFKANK-----GFKPI--SAEE-------------VP-----------------

---------------TTG----FKY-E----N---LSVS-ALPTA---------------

---------------VD----WRTKGAVT-------------------PIKNQG------

----QCGCCWAFSAVAAMEGIVKL-ST----------------DNLVSLSEQELVDC---

------DTH-----------------------SMDEGCEGG---------WMDSAFE---

-----------FVIKN-----G-GLATESS----------------Y-PYKAVDGK----

---------CKGG----------------SKSAAT----------------IKGHEDVPP

NNEAA--------------LMKAVA-SQPVSVAVDASDRTFMLYSG--------------

-----------------------GVMTG------------------------------SC

G-T-----Q-LDH-----------------------------------------------

-----------------------------GIAAIG-----------YGVES---------

--------------DGTKY---------------W------ILKNSWGTT----------

WG-----------------------EKRFLR--MEKDISD--------------------

-----------K-QGMCG-------------------------------LAMK-------

-------------PSYP---------TE--------------------------------

------------------------------------------------------------

------------------------------------------------------------

------------------------------------------------------------

------------------------------------------------------------

------------------------------------------------------------

------------------------------------------------------------

------------------------------------------------------------

------------------------------------------------------------

>MER0361053

------------------------------------------------------------

------------------------------------------------------------

------------------------------------------------------------

------------------------------------------------------------

------------------------------------------------------------

------------------------------------------------------------

------------------------------------------------------------

------------------------------------------------------------

--------------------------------MAERHERWMAEY----------------

-------DR--------------------------------------------VY-----

-------------------------KDA-------------------------------A

EKAR--------------------------------------------------------

-----------RFEVFKDN-----------------------------------------

------------------------------------------------------------

------------------------------FAFVESFN---------A-DK---KN----

------------------------------------------------KF----------

------------------------------------------------------------

---------------------------WLG--------------------------VNQF

ADLTTEEFKANK-----GFKPI--SAEE-------------VP-----------------

---------------TTG----FKY-E----N---LSVS-ALPTA---------------

---------------VD----WRTKGAVT-------------------PIKNQG------

----QCGCCWAFSAIAAMEGIVKL-ST----------------GNLVSLSEQEPVDC---

------DTH-----------------------NMDEGCEGG---------WMDNAFE---

-----------FVIKN-----G-GLATESS----------------Y-PYKVVDGK----

---------CKGG----------------SKSAAT----------------IKGHEDVPP

NNEAA--------------LMKVVA-SQPVSVAVDASDRTFMLYSG--------------

-----------------------GVMTG------------------------------SC

G-T-----Q-LDH-----------------------------------------------

-----------------------------GIAAIG-----------YGVES---------

--------------DDTKY---------------W------ILKNSWGTT----------

WG-----------------------EKGFLR--MEKDISD--------------------

-----------K-RGMCD-------------------------------LAMK-------

-------------PSYP---------TE--------------------------------

------------------------------------------------------------

------------------------------------------------------------

------------------------------------------------------------

------------------------------------------------------------

------------------------------------------------------------

------------------------------------------------------------

------------------------------------------------------------

------------------------------------------------------------

>MER0361056

------------------------------------------------------------

------------------------------------------------------------

----------------------------------MGI-----------------------

------------------------PKALLL------------------------------

-----------------------------AILGCGVCLCS--------------------

-------------------------------AA---------------------------

-------------------------------------------------VLAAR------

------------------------ELGGDD------EL----------------------

-------------------------------AMVARHEQWMVQH----------------

-------GR--------------------------------------------VY-----

-------------------------KDE-------------------------------T

DKAH--------------------------------------------------------

-----------RFLVFKAN-----------------------------------------

------------------------------------------------------------

------------------------------VKFIESFN---------A-AAAAGNR----

------------------------------------------------KF----------

------------------------------------------------------------

---------------------------WLG--------------------------VNQF

ADLTNDEFRATKTNK--GFNP---NVVK-------------VP-----------------

----------------TG----FRY-Q----N---LSID-ALPQT---------------

---------------VD----WRTKGAVT-------------------PIKDQG------

----QCGCCWAFSAVAATEGIVKI-ST----------------GKLTSLSEQELVDC---

------DVH-----------------------GEDQGCNGG---------EMDDAFK---

-----------FIIKN-----G-GLTTESN----------------Y-PYTAQDGQ----

---------CKSG----------------SNGAAT----------------IKGYEDVPA

NDEAA--------------LMKAVA-SQPVSVAVDGGDMTFQFYSG--------------

-----------------------GVMTG------------------------------SC

G-T-----D-LDH-----------------------------------------------

-----------------------------GIAAIG-----------YGKTS---------

--------------DGTKY---------------W------LMKNSWGTT----------

WG-----------------------ENGFLR--MEKDIAD--------------------

-----------K-KGMCG-------------------------------LAMQ-------

-------------PSYP---------TA--------------------------------

------------------------------------------------------------

------------------------------------------------------------

------------------------------------------------------------

------------------------------------------------------------

------------------------------------------------------------

------------------------------------------------------------

------------------------------------------------------------

------------------------------------------------------------

>MER0003753

------------------------------------------------------------

------------------------------------------------------------

----------------------------------MRP-----------------------

------------------------SSRYPPQP----------------------------

-----------------------------ALLALALALAA--------------------

-------------------------------AAPELR-----------------------

---------------------------------------PVAA-AV-AVTVTPP------

------------------------PERT--------DE----------------------

-------------------------------EVRRLYEEWRSEH--DAGPRR--------

-------GA--------------------------------------------TG-----

-------------------------GSLGPGDADAGAGAGE------------------D

DDAR--------------------------------------------------------

-----------RLEVFRDN-----------------------------------------

------------------------------------------------------------

------------------------------LRYIDAHN---------A-EADAGLH----

------------------------------------------------GF----------

------------------------------------------------------------

---------------------------RLG--------------------------LTRF

ADLTLEEYRARLLL---GSRG---RNGT-------------AVG----------------

---------------VVG---RRRY-L----P---LAGE-QLPDA---------------

---------------VD----WRERGAVA-------------------EVKDQG------

----QCGGCWAFSAVAAVEGINKI-VT----------------GSLISLSEQELIDC---

------DK------------------------FQDQGCDGG---------LMDNAFV---

-----------FMIKN-----G-GIDTEAD----------------Y-PFTGHDGT----

---------CDLKLK--------------NTRVVS----------------IDSFERVPI

NYERA--------------LQKAVA-HQPVSASIEASRRAFQLYSS--------------

-----------------------GIFDG------------------------------RC

G-T-----Y-LDH-----------------------------------------------

-----------------------------GVTVVG-----------YG-SE---------

--------------GGKDY---------------W------IVKNSWGTQ----------

WG-----------------------EAGYVR--MARNVRV--------------------

-----------R-PPSAG-------------------------------IAME-------

-------------PLYP---------VKE---------------------GPNP------

--------P-PGP-------------------------------TPPSPVK---------

------------------------------------------------------------

---------------------------------PPN------------------------

------------------------------------VCNAEY------------------

--------------------------------SCPEATTCC-------------------

---------------------------------------------CVSEYRGKCLAYG-C

CELENATCCE----DHSSCCPHDYPVCSVRDGTCRKSANSPMM-------VKALQRKPAM

YTGGGGGGE----------------QSGRSSW----------------------------

>MER0003752

------------------------------------------------------------

------------------------------------------------------------

----------------------------------MRP-----------------------

------------------------TRSAVSAT----------------------------

-----------------------------ALLLLAVALAL--------------------

-------------------------------AATAAA-----------------------

--------------------------------------RHSYT-TT-TTRVPAP------

------------------------AERA--------DE----------------------

-------------------------------EVRRMYEAWKSKH----------------

-------GR--------------------------------------------GG-----

-------------------------SSNDDCDMAPGDDEQEE-----------------E

DRRL--------------------------------------------------------

-----------RLEVFRDN-----------------------------------------

------------------------------------------------------------

------------------------------LRYIDAHN---------A-EADAGLH----

------------------------------------------------TF----------

------------------------------------------------------------

---------------------------RLG--------------------------LTPF

ADLTLEEYRGRVL----GFRA---RGRR-------------SGA----------------

---------------RYG----SGY------S---VRGG-DLPDA---------------

---------------ID----WRQLGAVT-------------------EVKDQQ------

----QCGGCWAFSAVAAIEGVNAI-AT----------------GNLVSLSEQEIIDC---

------D-------------------------AQDSGCDGG---------QMENAFR---

-----------FVIGN-----G-GIDTEAD----------------Y-PFIGTDGT----

---------CDASKEK-------------NEKVAT----------------IDGLVEVAS

NNETA--------------LQEAVA-IQPVSVAIDASGRAFQHYSS--------------

-----------------------GIFNG------------------------------PC

G-T-----S-LDH-----------------------------------------------

-----------------------------GVTAVG-----------YG-SE---------

--------------SGKDY---------------W------IVKNSWSAS----------

WG-----------------------EAGYIR--MRRNVPR--------------------

-----------P-TGKCG-------------------------------IAMD-------

-------------ASYP---------VKD---------------------TYHP------

--------GTGTA-------------------------------TARAAA----------

------------------------------------------------------------

------------------------------------------------------------

------------------------------------------------------------

------------------------------------------------------------

------------------------------------------------------------

--------------------------------------------------MDVIKMVLA-

------------------------------------------------------------

>MER0362201

------------------------------------------------------------

------------------------------------------------------------

----------------------------------MNG--PP-------------------

-------------------SPSSSSRLSVALL-------------LMI------------

-----------------------------TVLACGFVLAS--------------------

-------------------------------SGRSYA-----------------------

-----------------------------------------------HADYA--------

------------------------DGSD--------QELLMS------------------

-------------------------------TEWFRFHAWMAAH----------------

-------GR--------------------------------------------SY-----

-------------------------PTA-------------------------------E

EKLR--------------------------------------------------------

-----------RFHIYRAN-----------------------------------------

------------------------------------------------------------

------------------------------VELIEATN---------R-DT---SK----

------------------------------------------------TF----------

------------------------------------------------------------

---------------------------TCG--------------------------ENQF

TDLSHHEFLAMYT----MAGH---SAPP-------------LLNLSSVITT---------

-----RAGDITESDRGTTQVEE---------D---EEVE-ALPEN---------------

---------------ID----WREQNAVT-------------------PVQDQRR-----

----GCNACWVFASVATMESAHKI-KTN------------HGHGELLKLSEQEIVDC---

------TS---------------------------QHCGGG---------YPDDAFS---

-----------WVKRN-------GIATESE----------------YGGYEATVDS----

---------CRADMV--------------RPPAVR----------------VKDYSFVPK

NSEKK--------------LAMRVA-QQPVAVLFDATDPCFQCYTN--------------

-----------------------GIYSG------------------------------RP

A-AAADRYNILNH-----------------------------------------------

-----------------------------AMAIVG-----------YGEDKT--------

--------------TGRKY---------------W------IAKNSWGTR----------

WG-----------------------QNGYVY--IRKDMAD--------------------

-----------RPEGVGG-------------------------------LATH-------

-------------PRYP---------IV--------------------------------

------------------------------------------------------------

------------------------------------------------------------

------------------------------------------------------------

------------------------------------------------------------

------------------------------------------------------------

------------------------------------------------------------

------------------------------------------------------------

------------------------------------------------------------

>MER0361029

------------------------------------------------------------

------------------------------------------------------------

----------------------------------MSRC----------------------

------------------------------------------------------------

-----------------------------LVLAAVLLAAL--------------------

-------------------------------ALAPA------------------------

--------------------------------------------AR-GIPFSER------

------------------------DLAS--------EE----------------------

-------------------------------SLRALYERWRSHY----------------

-------HR--------------------------------------------VS-----

--------------P-------RDGDDK-------------------------------Q

QQAR--------------------------------------------------------

-----------RFNVFKEN-----------------------------------------

------------------------------------------------------------

------------------------------ARYVHEAN---------R-KD---GR----

------------------------------------------------PF----------

------------------------------------------------------------

---------------------------RLA--------------------------LNKF

ADMTTDEFRRTYA----GSRT---RHHR-------------AQLGEARS-----------

-----------FAHAQHG-------RG----G---SGTT-NLPPA---------------

---------------VD----WRLRGAVT-------------------GVKDQG------

----QCGSCWAFSAIAAVEGVNKI-MT----------------GKLVSLSEQELVDC---

------DD------------------------VDNQGCDGG---------LMDYAFQ---

-----------YIQRN-----G-GVTTESN----------------Y-PYLAEQRS----

---------CNKAKE--------------RSHDVT----------------IDGYEDVPA

NNEDA--------------LQKAVA-SQPVAVAIEASGQDFQFYSE--------------

-----------------------GVFTG------------------------------SC

G-T-----D-LDH-----------------------------------------------

-----------------------------GVAAVG-----------YGTTG---------

--------------DGTKY---------------W------TVKNSWGED----------

WG-----------------------ERGYIR--MQRGVPD--------------------

-----------S-RGLCG-------------------------------IAME-------

-------------PSYP---------TKK---------------------PAGHG-----

--------------------------------------------------G---------

------------------------------------------------------------

-----------------GI-----------------------------------------

------------------------------------VQDLGQ------------------

------------------------------ELHQADGGRASYY-----------------

------------------------------------------------------------

------------------------------------------------------------

------------------------------------------------------------

>MER0363147

------------------------------------------------------------

------------------------------------------------------------

----------------------------------MPAS----------------------

-----------------DRPPKNPATMALQL-----------------------------

-----------------------------AVLLLLLPLLA--------------------

-------------------------------GASA-------------------------

------------------------------------------------------------

--DDKFIRQVTT-----------QGTRA--------GA----------------------

---------------------------GPGLLPEAQFAAFVRRH----------------

-------GR--------------------------------------------RY-----

-------------------------SGP-------------------------------K

EYAR--------------------------------------------------------

-----------RLRVFAAN-----------------------------------------

------------------------------------------------------------

------------------------------LARAAAHQ---------A-LD----P----

------------------------------------------------TA----------

------------------------------------------------------------

---------------------------RHG--------------------------VTPF

SDLTREEFEARLT----GLRAG-GDVQR-------------LMSGVPAAPPA--------

--------------------------S----K---EEVA-RLPAS---------------

---------------FD----WRDKGAVT-------------------GVKTQG------

----ACGSCWAFSTTGAVEGANFL-AT----------------GELVDLSEQQLVDC---

------DHTCSAVAQN----------------ECNNGCAGG---------LMTNAYS---

-----------YLMES-----G-GLMEQSA----------------Y-PYTGAAGP----

---------CRFDP---------------TQVAVR----------------VANFTAVPA

GDEAQ--------------IRAALVRRGPLAVGLNAA--FMQTYVG--------------

-----------------------GVSCP-----------------------------LIC

P-R-----AWVNH-----------------------------------------------

-----------------------------GVLLVG-----------YGARGFAA------

-----------LRLGYRPY---------------W------IIKNSWGKQ----------

WG-----------------------EQGYYR--LCRG-----------------------

-------------SNVCG-------------------------------VDSM-------

-------------VSAV---------AVA-------------------------------

--------P------------------------------------------AL-------

------------------------------------------------------------

------------------------------------------------------------

------------------------------------------------------------

------------------------------------------------------------

------------------------------------------------------------

------------------------------------------------------------

------------------------------------------------------------

>MER0137791

------------------------------------------------------------

------------------------------------------------------------

----------------------------------MPS-----------------------

-----------------------VHHHLL-------------------------------

-----------------------------LLTLAALAVAA--------------------

-------------------------------ATAS-------------------------

------------------------------------------------------------

------------------------AGGD--------PP----------------------

-------------------------------AIEAQFDAWCAEH----------------

-------GK--------------------------------------------AY-----

-------------------------ATP-------------------------------E

ERAA--------------------------------------------------------

-----------RLAVFADN-----------------------------------------

------------------------------------------------------------

------------------------------AAFVAAHN---------A-RAGANAA----

-----------GGGGGGAAPP---------------------------SY----------

------------------------------------------------------------

---------------------------TLA--------------------------LNAF

ADLTHEEFRAARL----GRIA-----PG-------------AALRSRAA-----------

----------------------PVYWG----L---GGGA-AVPDA---------------

---------------LD----WRKSGAVT-------------------KVKDQG------

----SCGACWSFSATGAMEGINKI-KT----------------GSLVSLSEQELIDC---

------DR------------------------SYNSGCGGG---------LMDYAYK---

-----------FVIKN-----G-GIDTEED----------------Y-PYREADGT----

---------CNKNKL--------------KKRVVT----------------IDGYTDVPS

NKEDL--------------LLQAVA-QQPVSVGICGSARAFQLYYQ--------------

-----------------------GIFDG------------------------------PC

P-T-----S-LDH-----------------------------------------------

-----------------------------AVLIVG-----------YG-SE---------

--------------GGKDY---------------W------IVKNSWGES----------

WG-----------------------MKGYMH--MHRNTGD--------------------

-----------S-KGVCG-------------------------------INMM-------

-------------ASFP---------TKT---------------------SPNP------

--------PPSPG---------------------------------PGPT----------

------------------------------------------------------------

------------------------------------------------------------

------------------------------------KCSLLT------------------

--------------------------------YCPEGSTCC-------------------

---------------------------------------------CSWRVLGFCLSWS-C

CELDNAVCCK----DNRYCCPHDYPVCDTGRGQCLKASGNFSA-------IEGIRRKQS-

-------------------------FSKAPSWTGWLEL---------------MDQ----

>MER0029941

------------------------------------------------------------

------------------------------------------------------------

------------------------------------------------------------

------------------------------------------------------------

------------------------------------------------------------

------------------------------------------------------------

------------------------------------------------------------

------------------------------------------------------------

------------------------------------------------------------

------------------------------------------------------------

------------------------------------------------------------

------------------------------------------------------------

------------------------------------------------------------

------------------------------------------------------------

------------------------------------------------------------

------------------------------------------------------------

------------------------------------------------------------

------------------------------------------------------------

------------------------------------------------------------

------------------------------------------------------------

------------------------------------------------------------

------------------------------------------------------------

------------------------------------------------------------

------------------------------------------------------------

------------------------------------------------------------

------------------------------------------------------------

------------------------------------------------------------

----------LNH-----------------------------------------------

-----------------------------AVTMVG-----------YGAES---------

--------------GGRKY---------------W------IVKNSWGEK----------

WG-----------------------EKGYFRGFASRGPSR--------------------

-----------T-SGA--------------------------------------------

------------------------------------------------------------

------------------------------------------------------------

------------------------------------------------------------

------------------------------------------------------------

------------------------------------------------------------

------------------------------------------------------------

------------------------------------------------------------

------------------------------------------------------------

------------------------------------------------------------

>MER0364718

------------------------------------------------------------

------------------------------------------------------------

----------------------------------MRRAATPRWK----------------

------------------------------------------------------------

-----------------------------GQVALAVAVKS---------------L----

-------------------------------SREAITV----------------------

--------------------------------------------VGPSVLVRERVPGSEG

VLDRLFRRKVAGGDAHIHGAGLRQEPKAFA------EE----------------------

------------------------------------------------------------

-------GVVAG-----------------------------------------SY-----

-------------------------KSC----------NDC---------CTQKF----G

SHTR------------------------SQLFPKMECFSDLDVNVHLVEVYAGLLRFLPK

GMWIC------YFTCYKKE----------------W------------------------

------------------------------------------------------------

------------------------LQGSPILLVLDGSDFPTALFLLFQFPS--GAK----

------------------------------------------------SF----CMDL--

----------------------------------------------------LEL----F

TPHGT----------------------VLS-----VEVVSVASVSDSCCTLESAD-GSIY

FSSSDHHFESNPNERAFGDKV---RSSGGTATHKGGGALCLASRGMLCLSGDGELCPPSR

GKQRPDGGSERHTQMASG----SRK----------MAADGDVPVA---ASCTFL------

---------------LER---WRRFLASSIGKGRRNTLLGQASFKRL-MLKQQGMRTTWE

YPFAVAGSCWAFSTIAAVEGINQI-VT----------------GDLISLSKQELVDC---

------DT------------------------SYNQGCNGG---------LMDYVFE---

-----------FIINN-----G-GIDTEKD----------------Y-PYKGTDGR----

---------CDVNRV--------------VSAALK-------------------SESLPV

VFIHA--------------RNNRDA-EMIMSALITAK------YFS--------------

-----------------------GAWRR--------------------------------

--Q-----NDLPH-----------------------------------------------

------------------------------------------------------------

------------------------------------------------------------

WN----------------------------------------------------------

-----------S------------------------------------------------

------------------------------------------------------------

------------------------------------------------------------

------------------------------------------------------------

------------------------------------------------------------

------------------------------------------------------------

------------------------------------------------------------

------------------------------------------------------------

------------------------------------------------------------

------------------------------------------------------------

>MER0694710

------------------------------------------------------------

------------------------------------------------------------

----------------------------------MVARRTR-------------------

-------------------RAEAAARVIEMYSKS-------------VTT-----PPTG-

-------------------SC--------MADLFAGLIAE-DMRKLATGKVREFSLSAKW

CPSLDSSY-DHSTLIWL----RARA----RRAASSPRAP--R-------RLFPKGSAFKA

YTRLWRLQQERRRD----------------LVAV-GLRRWE------IGEVASRIGQL--

----YYARYLRA-----------TEPRSLVGAYVFYEAIYSRDYFGVAAATVGTNSGGGG

VSRHQALLIRCKELQFIARFLVVSMLMWPTVACLALSQDWLLDQF---------------

--GVLHDGKK-------------------------------------------SYP----

--TILACMLVVSLEP-PLC-----VQDLGMLDILEWAPEISYIWTTMIDAVISKLESKTG

ENLM-----VTEIV----GPK-------QI-AEVLDNRKLMNGQGRTVD-----------

--------FRNTLIIMTSN-----------------------------------------

------------------------------------------------------------

------------------------------L--GTEHL---------L-AG----M----

------------------------------------------------VG----------

------------------------------------------------------------

---------------------------KNS--------------------------MKVA

RDLVMQEVESAH----------------------------------PAA-----------

------------MAAESA-----------------IDTN-TEHPE---------------

---------------PE----HKDLLRVY-------------------EVGDGS------

----TMGCLHSI-------GSDLT-QT----------------RELPELQQNVSMRS---

------GQEASTVGKDSSRTPTSVRELVVPVCSLESGVLGK---------RIKRSLTGGN

EMQSTQDKRSRKASMV-----NEAILQHVK----------------Y-QNPQAQMQ----

---------WCADD---------------DALQSSPRLVALKERRITVTLGVMNFGVVSV

DESQN--------------VAILIK-HGPLAIGINAA--YMQTYIE--------------

-----------------------GMPCP-----------------------------YIC

G-R-----H-LDH-----------------------------------------------

-----------------------------VVLLVG-----------YGAAGFAP------

-----------IHLKDKPY---------------W------IIKNSWGMN----------

WR-----------------------ENEYYK--ICR------------------------

-------------------------------------------------VPTFATS----

-------------AVTP-------------------------------------------

------------------------------------------------------------

------------------------------------------------------------

------------------------------------------------------------

------------------------------------------------------------

------------------------------------------------------------

-----------------------------------------------FMY----------

------------------------------------------------------------

------------------------------------------------------------

>MER0601069

------------------------------------------------------------

------------------------------------------------------------

------------------------------------------------------------

------------------------------------------------------------

------------------------------------------------------------

------------------------------------------------------------

------------------------------------------------------------

------------------------------------------------------------

------------------------------------------------------------

------------------------------------------------------------

------------------------------------------------------------

------------------------------------------------------------

---------------MTSN-----------------------------------------

------------------------------------------------------------

------------------------------L--GTEHL---------L-AG----M----

------------------------------------------------VG----------

------------------------------------------------------------

---------------------------KNS--------------------------MKVA

RDLVMQEVESAH----------------------------------PAA-----------

------------MAAESA-----------------IDTN-TEHPE---------------

---------------PE----HKDLLRVY-------------------EVGDGS------

----TMGCLHSI-------GSDLT-QT----------------RELPELQQNVSMRS---

------GQEASTVGKDSSRTPTSVRELVVPVCSLESGVLGK---------RIKRSLTGGN

EMQSTQDKRSRKASMA-----G-GLEGEKD----------------Y-RYTRSDGK----

---------CKFDK---------------SKIIAS----------------IQNFGVVSV

DESQN--------------VAILIK-HGPLAIGINAA--YMQTYIE--------------

-----------------------GMPCP-----------------------------YIC

G-R-----H-LDH-----------------------------------------------

-----------------------------VVLLVG-----------YGAAGFAP------

-----------IHLKDKPY---------------W------IIKNSWGMN----------

WR-----------------------ENEYYK--ICXGSNI--------------------

-------------CNKCG------------------------------------------

------------------------------------------------------------

------------------------------------------------------------

------------------------------------------------------------

------------------------------------------------------------

------------------------------------------------------------

------------------------------------------------------------

------------------------------------------------------------

------------------------------------------------------------

------------------------------------------------------------

>MER0601151

------------------------------------------------------------

--------MA-----------------------A-ISLRLSTSSSQAHSKTST-------

----------------------------------MAAY----------------------

----------------------QQAPALL-------------------------------

-----------------------------CACLMLVLMAG--------------------

-------------------------------AASGGR-----------------------

------------------------------------------------------------

------------------------VDVE--------DM----------------------

-------------------------------LMMDRFRGWQATY----------------

-------NR--------------------------------------------SY-----

-------------------------LTA-------------------------------A

ERLR--------------------------------------------------------

-----------RFEVYRQN-----------------------------------------

------------------------------------------------------------

------------------------------MELIEATN---------R-RA---GL----

------------------------------------------------SY----------

------------------------------------------------------------

---------------------------QLG--------------------------ETPF

TDLTSEEFLATHT----MSTR---LHAS-------------EAARRHRELIT--------

----THAGPVSDGGRQWN----RNY----------TTDL-DVPES---------------

---------------VD----WRTKGAVT-------------------PVKDQG------

----ACGSCWSFVTVAAIEGLHKI-RT----------------GQLVSLSEQAVLDC---

------SS------------------------PPNHGCNRG---------DPAAAID---

-----------WVSAN-----G-GLTTESD----------------Y-PYVGRQGK----

---------CKLDKA--------------RNHVAK----------------IKGRKLVDQ

NNEAA--------------LEVAVA-QQPVAVDMNVD-PILQHYKS--------------

-----------------------GVFHG------------------------------PC

DPE-----D---------------------------------------------------

------------------------------------------------------------

------------------------------------------------------------

-----------------------------------RQPRR--------------------

-----------H-HGGVR------------------------------------------

------------------------------------------------------------

------------------------------------------------------------

------------------------------------------------------------

------------------------------------------------------------

------------------------------------------------------------

------------------------------------------------------------

---------------------------------------------C--------------

------------------------------------------------------------

------------------------------------------------------------

>MER0600967

------------------------------------------------------------

------------------------------------------------------------

----------------------------------M-------------------------

------------------------------------------------------------

------------------------------------------------------------

------------------------------------------------------------

------------------------------------------------------------

------------------------------------DD----------------------

-------------------------------SSLTSLSSWKMPL----------------

------------------------------------------------------------

-----------------------------------------------------------D

QSAR--------------------------------------------------------

------------------------------------------------------------

------------------------------------------------------------

------------------------------------------------------------

------------------------------------------------------------

------------------------------------------------------------

------------------------------------------------------------

------------------------------------------------------------

---------------VSS----FTY-HINVTS---VTVN-ALPMT---------------

---------------ID----WRTKGVIT-------------------PIKDQG------

----QCSCYWALSVVVATEGIVKI-TM----------------GKLVSLAEQELVDC---

------DVH-----------------------GEDQGCEGG---------LMDNAFK---

-----------FIIKN-----D-GLTIESS----------------Y-PYTAADGM----

---------CKSA----------------SNSAAT----------------IKGYEDVPA

NDEAA--------------LMKAVA-N---------------------------------

------------------------------------------------------------

------------------------------------------------------------

------------------------------------------------------------

------------------------------------------------------------

------------------------------------------------------------

------------------------------------------------------------

------------------------------------------------------------

------------------------------------------------------------

------------------------------------------------------------

------------------------------------------------------------

------------------------------------------------------------

------------------------------------------------------------

------------------------------------------------------------

------------------------------------------------------------

------------------------------------------------------------

>MER0600398

------------------------------------------------------------

------------------------------------------------------------

----------------------------------MIL-----------------------

------------------------------------------------------------

------------------------------------------------------------

------------------------------------------------------------

------------------------------------------------------------

------------------------------------------------------------

------------------------------------------------------------

------------------------------------------------------------

------------------------------------------------------------

------------------------------------------------------------

------------------------------------------------------------

------------------------------------------------------------

------------------------------------------------------------

------------------------------------------------SF----------

------------------------------------------------------------

------------------------------------------------------------

------------------------------------------------------------

------------------------------------------------------------

-------------------------------------------------IKNFS------

----------SSSLSHVIEGHQILFVS----------------GVLLTLGSKPTVQF---

-FTKPKDHKCDSSEPD----------------LCDSGCNGV---------QMTRP-S---

-----------VISKA-----G-GLEREKD----------------Y-RYTGSDGK----

---------CKFDK---------------SKIIAS----------------IQNFSVV--

------------------------------------------------------------

------------------------------------------------------------

------------------------------------------------------------

------------------------------------------------------------

------------------------------------------------------------

------------------------------------------------------------

----------------CG------------------------------------------

------------------------------------------------------------

------------------------------------------------------------

------------------------------------------------------------

------------------------------------------------------------

------------------------------------------------------------

------------------------------------------------------------

------------------------------------------------------------

------------------------------------------------------------

------------------------------------------------------------

>MER0158385

------------------------------------------------------------

------------------------------------------------------------

----------------------------------MVR-----------------------

-----------------------AAEVATTMA----------------------------

-----------------------------AALVVVIALST--------------------

------------------------------TPAAS-------------------------

-----------------------------------------------AIDYTEH------

------------------------DLAS--------EE----------------------

-------------------------------SLWALYERWCAHY----------------

-------NMA--------------------------------------------------

-------------------------RDL-------------------------------G

EKTR--------------------------------------------------------

-----------RFNLFKEN-----------------------------------------

------------------------------------------------------------

------------------------------AHRIYEHN---------Q-----GNA----

------------------------------------------------TY----------

------------------------------------------------------------

---------------------------TLG--------------------------LNRF

SDMTDEEFSRSPY----GRCLF-APVQR-------------ISDGENEELQQHEDV----

-----------SFNLTHG--------G----A---TAAL-GLPPS---------------

---------------VD----WRGR-SVT-------------------RVKDQGL-----

----TCGSCWAFAAIAAVEGINAI-RT----------------WSLVTLSEQQLVDC---

------D-------------------------NVDHGCAGG---------WIPSALD---

-----------FIVRN-----R-GIVPEGT----------------Y-PYIGTQGR----

---------CRHV----------------MAPPVT----------------IDGYRRVLP

FDVNA--------------LMSAVA-AQPVAVAMESSAWAFRHYQG--------------

-----------------------GVFNG------------------------------NC

G-G-----R-LGH-----------------------------------------------

-----------------------------AAAVVG-----------YG-DG---------

--------------AGGPF---------------W------IVKNSWGPK----------

WG-----------------------EGGYVR--ISRNAPN--------------------

-----------R-LGICG-------------------------------ILTQ-------

-------------PLYP---------VKR-------------------------------

------------------------------------------------------------

------------------------------------------------------------

------------------------------------------------------------

------------------------------------------------------------

------------------------------------------------------------

------------------------------------------------------------

------------------------------------------------------------

------------------------------------------------------------

>MER0136980

------------------------------------------------------------

------------------------------------------------------------

----------------------------------MDQSNIS-------------------

------------------NKHMTMTTLMLLL-----------------------------

-----------------------------CVIAIADCICH--------------------

-------------------------------AAVAAR-----------------------

--------------------------------------------VEPSTTVGRT------

------------------------TGGD--------EA----------------------

-------------------------------MMMARYKKWMAQY----------------

-------RR--------------------------------------------KY-----

-------------------------KDD-------------------------------A

EKAH--------------------------------------------------------

-----------RFQVFKAN-----------------------------------------

------------------------------------------------------------

------------------------------AEFIDRSN---------A-GG---KK----

------------------------------------------------KY----------

------------------------------------------------------------

---------------------------VLG--------------------------TNQF

ADLTSKEFAAMYT----GLRK---PAAV-------------PSGAKQIP-----------

---------------AAG----SKY-Q----N---FTRL-DDDVQ---------------

---------------VD----WRQQGAVT-------------------PVKNQG------

----QCGCCWAFSAVGAMEGLIMI-TT----------------GNLVSLSEQQILDC---

------DES-----------------------DGNQGCNGG---------YMDNAFQ---

-----------YVINN-----G-GVTTEDA----------------Y-PYSAVQGT----

---------CQN-----------------VQPAAT----------------ISGFQDLPS

GDENA--------------LANAVA-NQPVSVGVDGGSSPFQFYQG--------------

-----------------------GIYDG-----------------------------DGC

G-T-----D-MNH-----------------------------------------------

-----------------------------AVTAIG-----------YGADD---------

--------------QGTQY---------------W------ILKNSWGTG----------

WG-----------------------ENGFMQ--LQMGV----------------------

--------------GACG-------------------------------ISTM-------

-------------ASYP---------TP--------------------------------

------------------------------------------------------------

------------------------------------------------------------

------------------------------------------------------------

------------------------------------------------------------

------------------------------------------------------------

------------------------------------------------------------

------------------------------------------------------------

------------------------------------------------------------

>MER0141828

------------------------------------------------------------

------------------------------------------------------------

----------------------------------MHAK----------------------

--------------------MTKPAAIVVAAI----------------------------

-----------------------------AVLSVSLLAGS--------------------

-------------------------------SCLALA-----------------------

--------------------------------------RPSGD-FS-IVGYSEE------

------------------------DLSS--------HE----------------------

-------------------------------SLAELFERWLSRH----------------

-------RR--------------------------------------------AY-----

-------------------------ASL-------------------------------E

EKLR--------------------------------------------------------

-----------RFQVFKDN-----------------------------------------

------------------------------------------------------------

------------------------------LHHIDETN---------R-KV----S----

------------------------------------------------SY----------

------------------------------------------------------------

---------------------------WLG--------------------------LNEF

ADLTHDEFKATYL----GLRS---SVGD-------------GGSGIDDDDE---------

--------------PEEE----EGY-E----G---VDGA-SLPKS---------------

---------------VD----WRSKGAVT-------------------GVKNQG------

----QCGSCWAFSTVAAVEGINQI-VT----------------GNLTALSEQELIDC---

------DT------------------------DGNNGCNGG---------LMDYAFS---

-----------YIAHN-----G-GLHTEEA----------------Y-PYLMEEGT----

---------CQRSSSSEKKWPGSSEDANDDAAVVT----------------ISGYEDVPR

NNEQA--------------LLKALA-QQPVSVAIEASGRNFQFYSG--------------

-----------------------GVFDG------------------------------PC

G-T-----Q-LDH-----------------------------------------------

-----------------------------GVAAVG-----------YGTAA---------

--------------KGHDY---------------I------IVKNSWGPS----------

WG-----------------------EKGYIR--MRRGTGK--------------------

-----------R-QGLCG-------------------------------INKM-------

-------------ASYP---------TKN-------------------------------

------------------------------------------------------------

------------------------------------------------------------

------------------------------------------------------------

------------------------------------------------------------

------------------------------------------------------------

------------------------------------------------------------

------------------------------------------------------------

------------------------------------------------------------

>MER0139038

------------------------------------------------------------

------------------------------------------------------------

----------------------------------MAL-----------------------

------------------------SRAAASGG------------FALI------------

-----------------------------LLACCSLIMLA--------------------

-------------------------------AASGGG-----------------------

-----------------------------------------------GGGVDDD------

------------------------GVGG--------DR----------------------

-------------------------------LMMDRFLSWQATY----------------

-------NR--------------------------------------------SY-----

-------------------------PTA-------------------------------E

ERQR--------------------------------------------------------

-----------RFQVYRRN-----------------------------------------

------------------------------------------------------------

------------------------------IEHIEATN---------R-AG---NL----

------------------------------------------------TY----------

------------------------------------------------------------

---------------------------TLG--------------------------ENQF

ADLTEEEFLDLYT----MKGM---PVRR-------------DAGKKRANV----------

--------------------------S----S---SAAV-DAPTS---------------

---------------VD----WRSKGAVT-------------------PIKNQGP-----

----SCSSCWAFVTAATIESITKI-TT----------------GRLVSLSEQELIDC---

------D-------------------------PYDGGCNLG---------YFVNGYR---

-----------WVIQN-----G-GLTTEAN----------------Y-PYQARRYA----

---------CSRSRA--------------AQHAAT----------------ISDYVQLPA

G-EGQ--------------LQQAVA-QQPVAAAIEMGG-SLQFYSG--------------

-----------------------GVFSG------------------------------QC

G-T-----R-MNH-----------------------------------------------

-----------------------------AITVVG-----------YGADSS--------

--------------SGLKY---------------W------LVKNSWGQS----------

WG-----------------------ERGYLR--MPPRRRA--------------------

-------------RGTVR-------------------------------HRAR-------

-------------PRVPGRVIH----ILSI------------------------------

------------------------------------------------------------

------------------------------------------------------------

------------------------------------------------------------

------------------------------------VCMHDA------------------

------------------------------YVRARLLVGCCI------------------

------------------------------------------------------------

--------------SAARTCIHIY--------------------TYYYYGLICMRRRIPR

IYTHEN---------------------------------------------------YYV

>MER0361134

------------------------------------------------------------

------------------------------------------------------------

----------------------------------MATH----------------------

-----------------------YSSAFVLL-----------------------------

-----------------------------SVVAWACALSG--------------------

------------------------------------------------------------

-------------------------------------------------SLAAR------

------------------------DLADQ-------DQ----------------------

-------------------------------AMVARHEEWMAKY----------------

-------DR--------------------------------------------VY-----

-------------------------SDA-------------------------------A

EKAR--------------------------------------------------------

-----------RFEVFKAN-----------------------------------------

------------------------------------------------------------

------------------------------MALIESVN---------A-----GNH----

------------------------------------------------KF----------

------------------------------------------------------------

---------------------------WLE--------------------------ANRF

ADLTDDEFRATWT----GYRP---KTAA-------------ASSKGRSRT----------

--------------ATTG----FKY-A----N---VSLD-DVPAS---------------

---------------VD----WRTKGAVT-------------------PIKNQG------

----ECGCCWAFSAVASMEGVVKL-ST----------------GKLVSLSEQELVDC---

------DVN-----------------------GMDQGCEGG---------EMDDAFD---

-----------FIVGN-----G-GLTTESR----------------Y-PYTASDGT----

---------CNSNEA--------------SGDAAS----------------IKGYEDVPA

NDEAS--------------LRKAVA-NQPVSVAVDGGDSHFRFYKG--------------

-----------------------GVLSG------------------------------AC

G-T-----E-LDH-----------------------------------------------

-----------------------------GIAAVG-----------YGVAS---------

--------------DGTKY---------------W------VMKNSWGTS----------

WG-----------------------EAGYIR--MERDIAD--------------------

-----------E-EVLRDRRIIGGGRYKMSSVKANRGLVFVLLLIVLPALSIGERKLRDE

KDHKLYILSGNFLTTYTNLRIHILISLKAIFQTQGGVLFFEKRILVSILLNSGPFH----

------------------------------------------------------------

------------------------------------------------------------

------------------------------------------------------------

------------------------------------------------------------

---------------------------------------CCF------------------

------------------------------------------------------------

------------------------------------------------------------

------------------------------------------------------------

>MER0361908

------------------------------------------------------------

------------------------------------------------------------

----------------------------------MGA-----------------------

----------------------STTPLASAAA----------------------------

-----------------------------LLLLLLAPLAA--------------------

-------------------------------AADS-------------------------

--------------------------------------------MS-IVSY---------

------------------------GERS--------EE----------------------

-------------------------------EARRMYAEWMAAH----------------

-------GR--------------------------------------------TY-----

-------------------------NAV-------------------------------G

EEER--------------------------------------------------------

-----------RFEVFRDN-----------------------------------------

------------------------------------------------------------

------------------------------LRYVDAHN---------A-AADAGVH----

------------------------------------------------SF----------

------------------------------------------------------------

---------------------------RLG--------------------------LNRF

ADLTNDEYRATYL----GVRS---RPQR-------------ERR----------------

----------------LG----DRY-L----A---GDNE-DLPES---------------

---------------VD----WRAKGAVA-------------------EVKDQG------

----SCGSCWAFSTIAAVEGINQI-VT----------------GDMISLSEQELVDC---

------DT------------------------SYNQGCNGG---------LMDYAFE---

-----------FIINN-----G-GIDTEED----------------Y-PYKGTDGR----

---------CDVNRK--------------NAKVVT----------------IDSYEDVPA

NSEKS--------------LQKAVA-NQPISVAIEAGGRAFQLYNS--------------

-----------------------GIFTG------------------------------TC

G-N-----S-V-------------------------------------------------

------------------------------------------------------------

------------------------------------------------GP----------

WC-----------------------HGRRLR--HRE------------------------

------------------------------------------------------------

------------------------------------------------------------

------------------------------------------------------------

------------------------------------------------------------

------------------------------------------------------------

------------------------------------------------------------

------------------------------------------------------------

------------------------------------------------------------

-----------------------------RQG--LLDREELLG------------QQLGR

VRLRQDGAQ----------------HQGVQRQVWYRR-----------------------

>MER0363384

------------------------------------------------------------

------------------------------------------------------------

----------------------------------MFDACPSFCR----------------

--------EWLDYSFTGQGRQKQVNSVLSSLLKFHY--------PGLVKTSD--------

-----------------------------DADAAPVLVSCWDDYKLK-------------

---VDVTYGDAQGLVRADFWARFRLAD-GDDANHAEKVFQVNA-----YR----------

--------------------------------------------LVKEALYEARIQA---

--TCMYYQQILGQKMN-----KKTGASSI----YLREE----------------------

-------------------------------EYRKVVVPWMSNR----------------

-------PE--------------------------------------------AY-----

-------------------------AAW------------CGVWA--------------S

EAFQ----Q---------------------------------------------------

-----------KSKSHRDN-----------------------------------------

------------------------------------------------------------

------------------------------RGTMPNHTF----------GGD--------

------------------------------------------------NF----------

--------------------------------T--------------RKAKRLEA-----

---------------------------QLGREPTPIEVWEAGHRGSDP--------SNPL

CSQTQRERLAAYA-----------------------------------------------

-------------------------------E---EMKD-RHGED---------------

---------------CD----WRTM-----------------------PIDAQAV-----

---HKSGGGKAHGRFSLFDGM--INTTDFRASRRSASRSGSSGRSSRRLTEQDLEIVRLR

EENRERDEQLRAQTEQ----------------LRAQTQQQGIPYVA---PPPPPQWT---

-----------FVPMGPPPPPG-LVPQQPQ----------------F-STPPAQLP----

---------APGAQK--------------NAKVVT----------------IDSYEDVPA

NDEKS--------------LQKAVA-NQPVSVAIEAAGTAFQLYSS--------------

-----------------------GIFSG------------------------------SC

G-T-----A-LDH-----------------------------------------------

-----------------------------GVMAVG-----------YGTE----------

--------------NDKDY---------------W------IVKNSWGSS----------

WG-----------------------ESGYVR--MERNIKA--------------------

-----------S-SGKCG-------------------------------IVVE-------

-------------PSYP---------LKE---------------------GANP------

--------PNPGP-------------------------------SPPSPTP---------

------------------------------------------------------------

---------------------------------APA------------------------

------------------------------------VCDNYY------------------

--------------------------------SCPDSTTCC-------------------

---------------------------------------------CIYEYG---------

-----------------NCCPHDYPICNVRQGTCLMLKRKHNDRTE----NEAAESNDWM

SPGYANAGSS--------PVPTPPSGKGLKASTKPKATKG---QKSGPQTPLGFDE----

>MER0136360

------------------------------------------------------------

------------------------------------------------------------

----------------------------------MSA-----------------------

------------------------SRFLLA------------------------------

-----------------------------VLVVGSAVLCT--------------------

-------------------------------AAAPRA-----------------------

------------------------------------------------------------

------------------------LAAA--------AA----------------------

-------------------------------AMASRHEKWMAEH----------------

-------GR--------------------------------------------AY-----

-------------------------KDE-------------------------------A

EKAR--------------------------------------------------------

-----------RLEVFRAN-----------------------------------------

------------------------------------------------------------

------------------------------AELIDSFN---------A-AG---TH----

------------------------------------------------SH----------

------------------------------------------------------------

---------------------------RLA--------------------------TNRF

ADLTVEEFRAART----GLRP---RPAP-------------SAGAGR-------------

----------------------FRY-E----N---FSLA-DAAQS---------------

---------------VD----WRAMGAVT-------------------GVKDQG------

----ACGCCWAFSAVAAVEGLNKI-RT----------------GRLVSLSEQELVDC---

------DVS-----------------------GVDQGCDGG---------LMDNAFQ---

-----------FVARR-----G-GLASESG----------------Y-PYQGRDGP----

---------CRSSAA--------------AARAAS----------------IRGHEDVPR

NNEAA--------------LAAAVA-NQPVSVAINGEDMAFRFYDS--------------

-----------------------GVLGG------------------------------AC

G-T-----D-LNH-----------------------------------------------

-----------------------------AITAVG-----------YGTAN---------

--------------DGTRY---------------W------LMKNSWGAS----------

WG-----------------------EGGYVR--IRRGVRG--------------------

-------------EGVCG-------------------------------LAKL-------

-------------PSYP---------V---------------------------------

------------------------------------------------------------

------------------------------------------------------------

------------------------------------------------------------

------------------------------------------------------------

------------------------------------------------------------

------------------------------------------------------------

------------------------------------------------------------

------------------------------------------------------------

>MER0360959

------------------------------------------------------------

------------------------------------------------------------

----------------------------------MEPK----------------------

-------------------L----------------------------------------

-----------------------------AVAVFVLFLAF--------------------

-------------------------------AACSANHH---------------------

------------RD------------------------------PS-VVGYSQE------

------------------------DLA---------------------------------

-------------------------------LPSSLFRSWSVKH----------------

-------GK--------------------------------------------LY-----

-------------------------ASP-------------------------------T

EKLE--------------------------------------------------------

-----------RYEIFKQN-----------------------------------------

------------------------------------------------------------

------------------------------LMHIAETN---------R-----KNG----

------------------------------------------------SY----------

------------------------------------------------------------

---------------------------WLG--------------------------LNQF

ADVAHEEFKASYL----GLKR---ALPR-------------AGAPQTRT-----------

---------------PTA----FRY-A----A---AAAG-SLPWS---------------

---------------VD----WRYKGAVT-------------------PVKNQG------

----KCGSCWAFSSVAAVEGINQI-VT----------------GKLVSLSEQELVDC---

------DT------------------------TLDHGCEGG---------TMDLAFA---

-----------YMMGS-----Q-GIHAEDD----------------Y-PYLMEEGY----

---------CKEKQP--------------CVLGITEQ-------------DLTGFEDVPE

NSEIS--------------LLKALA-HQPVSVGIAAGSRDFQFYRG--------------

-----------------------GVFDG------------------------------AC

S-V-----E-LDH-----------------------------------------------

-----------------------------ALTAVG-----------YG-SS---------

--------------YGQNY---------------I------TMKNSWGKN----------

WG-----------------------EQGYVR--IKMGTGK--------------------

-----------P-EGVCG-------------------------------IYTM-------

-------------ASYP---------VKN---------------------ATRWGA----

------------------------------------------------------------

------------------------------------------------------------

------------------------------------------------------------

------------------------------------------------------------

------------------------------------------------------------

------------------------------------------------------------

------------------------------------------------------------

------------------------------------------------------------

>MER0360962

MHCV--------------------------------------------------------

------------------------------------------------------CFIHPE

QASN------------------------------MAWS----------------------

--------------------CARPMSIALAAV----------------------------

-----------------------------LLLCGGAWLQQ--------------------

-------------------------------AAEARPHH---------------------

------------MDDDSSI----------------DMDRGSDDFFS-IVGYSPE------

------------------------DLTQ--------HD----------------------

-------------------------------RLVRLFEEWVAKY----------------

-------RK--------------------------------------------AY-----

-------------------------GSF-------------------------------E

EKLR--------------------------------------------------------

-----------RFEVFKDN-----------------------------------------

------------------------------------------------------------

------------------------------LHHIDEAN---------R-KE---VT----

------------------------------------------------SY----------

------------------------------------------------------------

---------------------------WLG--------------------------LNAF

ADLTHDEFKATYL----GLLP---KRTS-------------GGR----------------

----------------------FRY-G--GVG---DGGD-EVPAS---------------

---------------VD----WRKKGAVT-------------------EVKNQG------

----QCGSCWAFSTVAAVEGINQI-VT----------------GNLTSLSEQQLVDC---

------ST------------------------DGNNGCSGG---------VMDNAFS---

-----------FIATG-----A-GLRSEEA----------------Y-PYLMEEGD----

---------CDDRAR-------------DGEVLVT----------------ISGYEDVPA

NDEQA--------------LVKALA-HQPVSVAIEASGRHFQFYSG--------------

-----------------------GVFDG------------------------------PC

G-S-----E-LDH-----------------------------------------------

-----------------------------GVAAVG-----------YG-SS---------

--------------KGQDY---------------I------IVKNSWGTH----------

WG-----------------------EKGYIR--MKRGTGK--------------------

-----------P-EGLCG-------------------------------INKM-------

-------------ASYP---------TKDH------------------------------

------------------------------------------------------------

------------------------------------------------------------

------------------------------------------------------------

------------------------------------------------------------

------------------------------------------------------------

------------------------------------------------------------

------------------------------------------------------------

------------------------------------------------------------

>MER0362671

------------------------------------------------------------

------------------------------------------------------------

----------------------------------MVS-----------------------

------------------------SKAFLLLL----------------------------

-----------------------------AVLIGCVCSFP--------------------

------------------------------------------------------------

-----------------------------------------------SPVLAAR------

------------------------ELSD--------DA----------------------

-------------------------------AMAERHERWMAEY----------------

-------GR--------------------------------------------VY-----

-------------------------KDA-------------------------------A

DKAR--------------------------------------------------------

-----------RFEVFKDN-----------------------------------------

------------------------------------------------------------

------------------------------FAFVESFN---------A-DK---KN----

------------------------------------------------KF----------

------------------------------------------------------------

---------------------------WLG--------------------------VNQF

ADLTTEAFKANK-----GFKPI--SAEK-------------AP-----------------

---------------TTG----FKY-E----N---LSIS-ALPTA---------------

---------------VD----WRTKGAVT-------------------PIKNQG------

----QCGCCWAFSAVAAVEGIVKL-ST----------------GNLVSLSEQELVDC---

------DTH-----------------------SMDEGCEGG---------WMDSAFE---

-----------FVIKN-----G-GLATESS----------------Y-PYKAVDGK----

---------CKGG----------------SKSAAT----------------IKGHEDVPP

NNEAA--------------LMKARG-QS--------------------------------

------------------------------------------------------------

------------------------------------------------------------

-------------------------------ACVG-------------------------

------------------------------------------------------------

------------------------------------------------------------

-------------RRRCK------------------------------------------

------------------------------------------------------------

------------------------------------------------------------

------------------------------------------------------------

------------------------------------------------------------

------------------------------------------------------------

------------------------------------------------------------

------------------------------------------------------------

------------------------------------------------------------

------------------------------------------------------------

>MER0138162

MHY---------------------------------------------------------

-----------------------------------INPATLLASSQDSRGTSY-------

---------------------------HIHHNMTMAT-----------------------

------------------------ASASLA------------------------------

-----------------------------LMFACSLLLAG--------------------

-------------------------------TAFS-------------------------

------------------------------------------------------------

------------------------DDTI--------AI----------------------

-------------------------------PLLERFKAWQAEY----------------

-------NR--------------------------------------------TY-----

-------------------------ATP-------------------------------E

EFQQ--------------------------------------------------------

-----------RFMIYSEN-----------------------------------------

------------------------------------------------------------

------------------------------VRFIKTMN---------QLST---GS----

------------------------------------------------SY----------

------------------------------------------------------------

---------------------------ELG--------------------------ENQF

TDLTEEEFKDTYL----MKLD---EQPP-------------AAEAMPPTVGT--------

---------MSTAGMSNG-----------------NNTG-EAPNS---------------

---------------VD----WRTKGAVT-------------------RVKDQQ------

----QCGSCWAFATVASIEGVHQI-KT----------------GRLVSLSEQEIVDC---

------DRG-----------------------GNDNGCRGG---------SPRSAME---

-----------WVTRN-----G-GLTTESD----------------Y-PYVGSQRQ----

---------CMSGKL--------------GHHAAR----------------IRGYQAVQR

NNEAE--------------LERAVA-GQPVAVFVDAS-RAFQFYKS--------------

-----------------------GVFSG------------------------------PC

DTT-----T-VNH-----------------------------------------------

-----------------------------VVTVVG-----------YGSTGSDS------

--------------GGRKY---------------W------IVKNSWGQG----------

WG-----------------------ENGYVR--MARRVRA--------------------

-----------R-EGMCA-------------------------------IAIE-------

-------------PYYP---------VM--------------------------------

------------------------------------------------------------

------------------------------------------------------------

------------------------------------------------------------

------------------------------------------------------------

------------------------------------------------------------

------------------------------------------------------------

------------------------------------------------------------

------------------------------------------------------------

>MER0361117

------------------------------------------------------------

------------------------------------------------------------

--------------------------------MTMAT-----------------------

------------------------ASASLALV----------------------------

-----------------------------MLFACSLLLAG--------------------

-------------------------------TAFS-------------------------

------------------------------------------------------------

------------------------DDTI--------AI----------------------

-------------------------------PLLERFKAWQAEY----------------

-------NR--------------------------------------------TY-----

-------------------------ATP-------------------------------E

EFQQ--------------------------------------------------------

-----------RFMVYSEN-----------------------------------------

------------------------------------------------------------

------------------------------LRFIKTMN---------QLST---GS----

------------------------------------------------SY----------

------------------------------------------------------------

---------------------------ELG--------------------------ENQF

TDLTEEEFKDTYL----MKLD---EQPP-------------AAEAMPPIVGT--------

---------MSTAGMSNG-----------------DNTG-EAPNS---------------

---------------VD----WRTKGAVT-------------------PVKNQQ------

----QCGSCWAFATVASIEGVHQI-KT----------------GRLVSLSEQEIVDC---

------DRG-----------------------GNDHGCRGG---------YPRSAME---

-----------WVTRN-----G-GLTTESD----------------Y-PYVGSQRQ----

---------CMSGKL--------------GHHAAR----------------IRGYQAVQR

KNEAE--------------LERAVA-GRPVAVVIDAS-RAFQFYKR--------------

-----------------------GVFSG------------------------------PC

NTT-----T-VNH-----------------------------------------------

-----------------------------AVTVVG-----------YGSAGSDSG-----

--------------GGRKY---------------W------IVKNSWGQR----------

WG-----------------------ENGYVR--MARRVRA--------------------

-----------R-EGMCA-------------------------------IAIE-------

-------------PLLPSD-------VI--------------------------------

------------------------------------------------------------

------------------------------------------------------------

------------------------------------------------------------

------------------------------------------------------------

------------------------------------------------------------

------------------------------------------------------------

------------------------------------------------------------

------------------------------------------------------------

>MER0361626

---------------------------------------------MDIVVEKGNVDVGNT

AEPS-YEVVSRSGCSAQLSTNDTDEHELESE-DSKVSVGNAAKQSHDVVGVGIDCIFNHV

KVSTEADLGPQPDVIHLTSLDVPNESSQICSDADMVKQ----------------------

----------------------NQQDALDTLIRISQHKKPKKLNVARVVSEDYKCTPEDV

QLIEYIKTLPGKQVVVNIDSAWLNRNDMECLFHGDMQLSD--------------------

-------------------------------KALNAYIHCIRGEEHLLHRE--GGKVFLE

NTFISSL---LKRDGDPKVFLPMNIEDFHWYLAVLNAKKSELKGLERQIKLAAKHKE---

----LYQGKWSN-----------LDVAS----WPVIEKITTQMQT-------------DG

VSC--------------------------GLWMINYMEYWTGSSLSDNVTQDDITMFRFK

LPAILWDSRLNTKKGHQNLDHNVDEDGESSSDVQIIDTPLTSTNTQELMFVLCTYIMGID

NDKYLKKHWIQSTKPYPISLSLQKLKDI--LDVNKPMDTDCFNMAVRMIACNDSL-FLLE

DKYHYMDLQFCSITKFGRDPRLRAKPDINMLAKLLECWPDMEYDVSDCKQGF--------

-IWLSCYQLYARLEWYKVT------------------------LH---------------

------------------------------------------------------------

------------------------------LHFVESKDRRISVA--LAFAGHQEEKCSVP

MHVPLRPWQQQGYGNSDVVSLVDLIQILNHKDCFCIFGYDGDSTIGHRLYTEDVTVNFKH

NWMGKGGRLTKPVTNIYWETVATNLDEFLEISTLG---RGEKLSELQDKTSELYIQAQQF

KKQGVKIRRKTWLQNMKIKLVILGILLLLVI-----------------ITLGRGEKLSEL

QDKTSELYIQAQQFKKQGVKI---RRKT-------------WLQNMKIKLV---------

-----ILGILLLLVIIAS----FKR----------LMLK-QQGMR---------------

---------------TT----WEYPFAVA-------------------------------

------GSCWAFSTIAAVEGINQI-VT----------------GDLISLSEQELVDC---

------DT------------------------SYNQGCNGG---------LMDYAFE---

-----------FIINN-----G-GIDTEKD----------------Y-PYKGTDGR----

---------CDVNRK--------------NAKVVT----------------IDSYEDVPA

NDEKS--------------LQKAVA-NQPVSVAIEAAGTTFQLYSS--------------

-----------------------GIFTG------------------------------SC

G-T-----A-LDH-----------------------------------------------

-----------------------------GVTAVG-----------YG-TE---------

--------------NGKDY---------------W------IMKNSWGSS----------

WG-----------------------ESGRAP--TRRTLAP---------APAVCDNYYSC

PD---------S-TTCCC-------------------------------IYEY-------

-------------GKYC---------FAW---------------------GCCPLEGATC

CDDHYSCCPHDYPICNVRQGTCLMACI-----------------VRPSIN----------

------------------------------------------------------------

-------------IAAPGL--------------PPSEP--------------RE------

--GNT-------------------------------------------------------

------------------------------GNPAPTPPDCA-------------------

DRAGG------------------------------------------------------S

CPERAAQTAAPE--EPHRSCTHRSSLSN---GMCTMRMLLLLRWD---------RSKDFQ

TTTGSDAGPTCSVTKINLPVYHVYSVQGQR------------------------------

>MER0137198

MHR----------------------------------C----------------------

---------------------------------------RSSSSSPLPRGQAK-------

--------GPA-----------------------MAR-----------------------

------------------------SPRLL-------------------------------

-----------------------------ALLLAVVWICG--------------------

-------------------------------AALVAR-----------------------

------------------------------------------------------------

------------------------------------AD----------------------

-------------------------------PMLERFEQWMGRH----------------

-------GR--------------------------------------------LY-----

-------------------------ADA-------------------------------G

EKQR--------------------------------------------------------

-----------RLEVYRRN-----------------------------------------

------------------------------------------------------------

------------------------------VELVETFN---------S-MG----N----

------------------------------------------------GY----------

------------------------------------------------------------

---------------------------RLA--------------------------DNKF

ADLTNEEFRAKML----GFGR---PRSG-------------GGAGHSTAP----------

-----------STVACIG----SGLMG----R---QGYS-DLPKS---------------

---------------VD----WREKGAVA-------------------PVKSQG------

----DCGSCWAFSAVAAIEGINQI-KN----------------GKLVSLSEQELVDC---

------DTK-------------------------AIGCAGG---------YMSWAFE---

-----------FVMKN-----R-GLTTERN----------------Y-PYQGLNGA----

---------CQTPKL--------------KESAVS----------------ISGYMNVTP

SSEPD--------------LLRAAA-AQPVSVAVDAGSFVWQLYGG--------------

-----------------------GVFTG------------------------------PC

T-A-----E-LNH-----------------------------------------------

-----------------------------GVTVVG-----------YGETQGDTDGDG--

-----------SGVPGKKY---------------W------IVKNSWGPE----------

WG-----------------------DAGYIL--MQREASV--------------------

-----------A-SGLCG-------------------------------IAML-------

-------------PSYP---------VM--------------------------------

------------------------------------------------------------

------------------------------------------------------------

------------------------------------------------------------

------------------------------------------------------------

------------------------------------------------------------

------------------------------------------------------------

------------------------------------------------------------

------------------------------------------------------------

>MER0361189

------------------------------------------------------------

------------------------------------------------------------

----------------------------------MVKK----------------------

---------------------MGASRAL--------------------------------

-----------------------------VLAAAMLAMAV--------------------

-------------------------------AAKG-------------------------

-----------------------------------------------ALLLTDK------

------------------------DLES--------EE----------------------

-------------------------------SMWSLYERWRSVH----------------

-------TVS--------------------------------------------------

-------------------------RDL-------------------------------R

EKQS--------------------------------------------------------

-----------RFEAFKAN-----------------------------------------

------------------------------------------------------------

------------------------------ARHIGEFN---------K-RK---DV----

------------------------------------------------PY----------

------------------------------------------------------------

---------------------------KLG--------------------------LNKF

ADLTQEEFVSKYT----GAKV---VDSE-------------AAARLASGV----------

--------RVSSSDESPPQL-----------A---ASVG-DAPDA---------------

---------------WD----WRDHGAVT-------------------AVKDQG------

----QCGSCWAFSAVGAVESVNAI-VT----------------GNLLTLSEQQMLDC---

------S-------------------------GAGDCTYGG---------YTYYAML---

-----------YAISN-----GLTLDQCGK----------------T-PYYQRYDAQQHL

--------PCRFDAK--------------KPPVVK----------------IDSMYVMNN

ADEAA--------------LKRAVY-KQPVSVLIDAGG--IGYYSE--------------

-----------------------GVFTG------------------------------PC

G-T-----S-LNH-----------------------------------------------

-----------------------------AVLLVG-----------YGATA---------

--------------DGTKY---------------W------IVKNSWGAD----------

WG-----------------------EKGYFR--LKRDVGT--------------------

-----------Q-GGLCG-------------------------------ITMY-------

-------------PIYP---------IKN---------------------CPCP------

--------AAAAA-----------------------------------------------

------------------------------------------------------------

-------------VAA---------------Y----------------------------

------------------------------------------------------------

------------------------------------------------------------

------------------------------------------------------------

------------------------------------------------------------

------------------------------------------------------------

>MER0144026

M-CR-------SIHQSPQRPARSCCPQATSSSSVRRPCVVAASMQQRVSISRYYI----R

TSPVV--PMATTPHHHKHSQPAITDHSISSL--VYRTAPHPRARRRSPASTYI-CLAARA

K---------------------------------LAE-----------------------

-----------------------MARALPLVL------------AASV------------

-----------------------------LLAAAALLLAA--------------------

-------------------------------APAP-------------------------

--------------------------------------------AA-AVDFGAE------

------------------------DLAS--------EE----------------------

-------------------------------ALWALYERWRGRH----------------

-------ALA--------------------------------------------------

-------------------------RDL-------------------------------G

DKAR--------------------------------------------------------

-----------RFNVFKAN-----------------------------------------

------------------------------------------------------------

------------------------------VRLIHEFN---------R-RD----E----

------------------------------------------------PY----------

------------------------------------------------------------

---------------------------KLR--------------------------LNRF

GDMTADEFRRHYA----GSRV---AHHR-------------MFRGDRQG-----------

-----------SSASASS----FMY----------ADAR-DVPAS---------------

---------------VD----WRQKGAVT-------------------DVKDQG------

----QCGSCWAFSTIAAVEGINAI-KT----------------KNLTSLSEQQLVDC---

------DT------------------------KANAGCNGG---------LMDYAFQ---

-----------YIAKH-----G-GVAAEDA----------------Y-PYRARQAS----

---------CKK--S--------------PAPVVT----------------IDGYEDVPA

NDESA--------------LKKAVA-HQPVSVAIEASGSHFQFYSE--------------

-----------------------GVFSG------------------------------RC

G-T-----E-LDH-----------------------------------------------

-----------------------------GVAAVG-----------YGVTA---------

--------------DGTKY---------------W------LVKNSWGPE----------

WG-----------------------EKGYIR--MARDVAA--------------------

-----------K-EGHCG-------------------------------IAME-------

-------------ASYP---------VKT---------------------SPNP------

--------K--------------------------------------VHA----------

------------------------------------------------------------

------------------------------------------------------------

------------------------------------VVDEDG------------------

--------------------------------SSHDEL----------------------

------------------------------------------------------------

------------------------------------------------------------

------------------------------------------------------------

>MER0365117

---------------------------------------------MDIVVEKGNVDVGNT

AEPS-YEVVARSGCSAQLSTNDTDEHELESE-DSKVSVGNAAKQSHDVVGVGIDCIFNHV

KVSTEADLGPQPDVIHLTSLDVPNESSQISSDADMVKQ----------------------

----------------------NQQDALDTLIRISQHKKPKKLNVARVVSEDYKCTPEDV

QLIEYIKTLPGKQVVLNIDSAWLNRNDMECLFHGDMQLSD--------------------

-------------------------------NALNAYIHCIKGEEHLLHRE--GGKVFLE

NTFISSL---LKRDGDPKVFLPMNIEDFHWYLAVLNAKKSELKGLERQIKLAAKHKE---

----LYQGKWSN-----------LDVAS----WPVIEKITTQMQT-------------DG

VSC--------------------------GLWMINYMEYWTGSSLSDNVTQDDITMFMFK

LPAILWDSRLNTKKGHQNLDHNVDEDGESSSDVQIIDTPLTSTNAQELMFVLCTYIMGID

NDKYLKKHWIQSTKPYPISLSLQKLKDI--LDVNKPMDTDCFNMAVRMIACNDSL-FLLE

DKYHYMDLQFCSITKFGRDPRLRAKPDINMLAKLLECWPDMEYDVSDCKQI---------

--LLP-FSFLGHFTLYVLNMDTRSIYIMDSMPIPSWFKGDHPSMHYIHNIHYIANNMNAA

MELANPTWKDDIYMWRRIVPTWVPRTLNWDLSGFLVINFMHDWNGIRLPCICTVAGNWRL

VKPQTGATKVEEIHSKNHPVSLLVIEHSPFLAVVESKDRRISVA--LAFAGHQEEQCSVP

MHVPLRPWQQQGYGNSDV-------------------------DVDRRSYPERVVLNL--

----------------------LTVDATPRVTVSGAPADADPPSPWQRRS----------

---------------------------LLGP-----------------PTLGRGEKLSEL

QDKTSELYIQAQQFKKQGVKI---RRKT-------------WLQNMKIKLV---------

-----ILGILLLLVII--------------------------------------------

------------------------------------------------------------

------------------------------------------------------------

------------------------------------GCNGG---------LMDYAFE---

-----------FIINN-----G-GIDTEKD----------------Y-PYKGTDGR----

---------CDVNRK--------------NAKVVT----------------IDSYEDVPA

NDEKS--------------LQKAVA-NQPVSVTIEAAGTTFQLYSSVANSQNKATTAYTN

LIPFFPDDEQGATSFLYTEYKYVGLFMGIFAILIFLFLGSVKGFSTKSQPCH-YSKDKTC

K-P-----A-LANA------IFSTIAFVLGAVTSLFSGFLGIKIATYANAR-TTLEARKG

VGKAFITAFRSGAVMGFLLAASGLLVLYIAITLIGI---------YYG------------

-----------------DV---------------WEGLFEAITGYGLGGSSMA------L

FG-----------------------HVGGGI--YTKAVDVGADLVGKVIADNVGDNVGNI

AGMGSDLFGSYA-ESSCA-------------------------------ALVV-------

-------------ASIS----------SF---------------------GINH------

-----QFTPMVYPL--LVSSVGIIACLITTLFATDFFEIKVVNEIEPALKKQLIISTVVM

TISIALISWLGLPDTFTIYNFGARKTVQSWQLFLCVAVGLWAGLVIGFITEYYTSNAYSI

FLSFSLAAMYGADVAALGMLSTIATCLAIDAYGPISDNAGGIAEMAGMSHRIRERTDALD

AAGNTTAAIGKGFAIGSAALVSLALFGAFVSRVAISTVDVLTPKVFIGLIVGAMLPYWFS

AMTMKSVGSAALKMVEEVRRQFNTIPGLMEGTTKPDYATCVKISTDASIKEMISQIAISA

SNTGGAWDNAKKYIEVNLHADFTGPSFPCMNLHYTFSKKLFLKEKCLFIYNFLVFGFAQA

GASEHARTFGPKGSDPHKAAVIGYTIGDPLKDTSGPSLNILIK-------LMAVESLVFT

PFFAAHGGITCVLCN-GICIERQYT-EG--------------------------------

>MER0158450

------------------------------------------------------------

------------------------------------------------------------

----------------------------------MAAY----------------------

----------------------QQAPALL-------------------------------

-----------------------------CACLMLVLMAG--------------------

-------------------------------AASGGR-----------------------

------------------------------------------------------------

------------------------VDVE--------DM----------------------

-------------------------------LMMDRFRAWQATY----------------

-------NR--------------------------------------------SY-----

-------------------------LTA-------------------------------A

ERLR--------------------------------------------------------

-----------RFEVYRQN-----------------------------------------

------------------------------------------------------------

------------------------------MELIEATN---------R-RA---EL----

------------------------------------------------SY----------

------------------------------------------------------------

---------------------------QLS--------------------------ETPF

TDLTSEEFLATHT----MSTR---LHAS-------------EAARRHRELIT--------

----THAGPVSDGGRQWN---RRNY----------TTDL-DVPES---------------

---------------VD----WRTKGAVT-------------------TVKDQG------

----ACGGCWSFATVAAIEGLHKI-RT----------------GQLVSLSEQEVLDC---

------SS------------------------PPNNGCHGG---------NPAAAID---

-----------WVSAN-----G-GLTTESD----------------Y-PYEGRQGK----

---------CKLDKA--------------RNHVAK----------------IRGRKLVDQ

NNEAA--------------LEVAVA-QQPVAVGMNVH-PIQQHYKS--------------

-----------------------GVFHG------------------------------PC

DPE-----D-LNH-----------------------------------------------

-----------------------------AVTMVG-----------YGAES---------

--------------GGRKY---------------W------IVKNSWGEK----------

WG-----------------------EKGYFRGFASRGASR--------------------

-----------T-SGAPA-------------------------------V----------

------------------------------------------------------------

------------------------------------------------------------

------------------------------------------------------------

------------------------------------------------------------

------------------------------------------------------------

------------------------------------------------------------

------------------------------------------------------------

------------------------------------------------------------

------------------------------------------------------------

>MER0138703

------------------------------------------------------------

------------------------------------------------------------

----------------------------------MAPH----------------------

---------------------IVVNKTVITF-----------------------------

-----------------------------TAVALTILAVT--------------------

-------------------------------TMMAE------------------------

--------------------------------------------AR-DLSSTST------

------------------------GGYG--------EE----------------------

-------------------------------AMKVRHQQWMAEH----------------

-------GR--------------------------------------------TY-----

-------------------------RDE-------------------------------A

EKAH--------------------------------------------------------

-----------RFQVFKAN-----------------------------------------

------------------------------------------------------------

------------------------------ADFVDASN---------A-AGD-DKK----

------------------------------------------------SY----------

------------------------------------------------------------

---------------------------RLE--------------------------LNEF

ADMTNDEFMAMYT----GLRP---VPAG-------------AKK----------------

---------------MAG----FKY-G----NVTLSDAD-DDQQT---------------

---------------VD----WRQKGAVT-------------------GIKNQG------

----QCGCCWAFAAVAAVEGIHQI-TT----------------GNLVSLSEQQVLDC---

------DT------------------------DGNNGCNGG---------YIDNAFQ---

-----------YIVGN-----G-GLGTEDA----------------Y-PYTAAQAM----

---------CQS-----------------VQPVAA----------------ISGYQDVPS

GDEAA--------------LAAAVA-NQPVSVAIDAH--NFQLYGG--------------

-----------------------GVMTA-----------------------------ASC

S-T--PP-N-LNH-----------------------------------------------

-----------------------------AVTAVG-----------YGTAE---------

--------------DGTPY---------------W------LLKNQWGQN----------

WG-----------------------EGGYLR--LERGANA--------------------

----------------CG-------------------------------VAQQ-------

-------------ASYP---------VA--------------------------------

------------------------------------------------------------

------------------------------------------------------------

------------------------------------------------------------

------------------------------------------------------------

------------------------------------------------------------

------------------------------------------------------------

------------------------------------------------------------

------------------------------------------------------------

>MER0136246

------------------------------------------------------------

------------------------------------------------------------

----------------------------------MAS-----------------------

----------------------SSKRSLPCVL----------------------------

-----------------------------LLLALFHHGCS--------------------

-------------------------------SARAHR-----------------------

------------R----------------------------------AGDTMGS------

------------------------MSND--------DS----------------------

-------------------------------SMIERFQRWKAAY----------------

-------NK--------------------------------------------SY-----

-------------------------ATV-------------------------------A

EERR--------------------------------------------------------

-----------RFRVYARN-----------------------------------------

------------------------------------------------------------

------------------------------MAYIEATN---------A-EAEAAGL----

------------------------------------------------TY----------

------------------------------------------------------------

---------------------------ELG--------------------------ETAY

TDLTNQEFMAMYT----APAL---AQLP-------------ADESVITTR----------

------AGPVDAVGGAPGQL--PVY------V---NLSA-SAPAS---------------

---------------VD----WRASGAVT-------------------PVKNQG------

----RCGSCWAFSTVAVVEGIYQI-RT----------------GKLVSLSEQELVDC---

------DT-------------------------LDDGCDGG---------ISYRALR---

-----------WIASN-----G-GITTEAD----------------Y-PYTGTTDA----

---------CNRAKL--------------SHNAVS----------------IAGLRRVAT

RSEAS--------------LANAVA-GQPVAVSIEAGGDNFQHYKK--------------

-----------------------GVYNG------------------------------PC

G-T-----N-LNH-----------------------------------------------

-----------------------------GVTVVG-----------YGQEAA--------

--------------AGDRY---------------W------IVKNSWGQG----------

WG-----------------------DDGYIR--MKKDVAG--------------------

-----------KPEGLCG-------------------------------IAIR-------

-------------PSYP---------LM--------------------------------

------------------------------------------------------------

------------------------------------------------------------

------------------------------------------------------------

------------------------------------------------------------

------------------------------------------------------------

------------------------------------------------------------

------------------------------------------------------------

------------------------------------------------------------

>MER0158433

------------------------------------------------------------

------------------------------------------------------------

----------------------------------MAT-----------------------

------------------------TSALL-------------------------------

-----------------------------ALVLLASLLAG--------------------

-------------------------------TVFSDD-----------------------

------------------------------------------------------------

------------------------IVPI--------HI----------------------

-------------------------------PLLDRFQAWQAEY----------------

-------NR--------------------------------------------TY-----

-------------------------ATP-------------------------------E

EFQQ--------------------------------------------------------

-----------RFMVYSEN-----------------------------------------

------------------------------------------------------------

------------------------------VKFIETMN---------Q-----PGS----

------------------------------------------------SY----------

------------------------------------------------------------

---------------------------ELG--------------------------ENRF

ADLTEEEFKDTYL----MKLD---NVAS-------------SPEAMALTVDT--------

---------MNRAGTSGG-----------------SNTN-EAPNS---------------

---------------VD----WRTKGAVT-------------------PVKSQQ------

----HCGSCWAFAAVASIEGVHKI-KT----------------GLLVSLSEQEIVDC---

------DRG-----------------------GNNHGCHGG---------HSSSAME---

-----------WVTRN-----G-GLTTESD----------------Y-PYVGRQGQ----

---------CMSDKL--------------GHHAAK----------------IRGRQAVQG

KNEGA--------------LQHAVA-GRPVAVSINAS-RAFQFYKR--------------

-----------------------GIFSG------------------------------PC

N-T-----T-RNH-----------------------------------------------

-----------------------------AVTVVG-----------YGANA---------

--------------SGHKY---------------W------IVKNSWGER----------

WG-----------------------EKGYVR--MQRGVRA--------------------

-----------R-EGVCG-------------------------------IAIA-------

-------------PFYA---------VM--------------------------------

------------------------------------------------------------

------------------------------------------------------------

------------------------------------------------------------

------------------------------------------------------------

------------------------------------------------------------

------------------------------------------------------------

------------------------------------------------------------

------------------------------------------------------------

>MER0364571

------------------------------------------------------------

------------------------------------------------------------

----------------------------------MLG-----------------------

------------------------------------------------------------

------------------------------------------------------------

------------------------------------------------------------

------------------------------------------------------------

------------------------------------------------------------

------------------------------------------------------------

------------------------------------------------------------

-------------------------PSA--------------------------------

------------------------------------------------------------

------------------------------------------------------------

------------------------------------------------------------

----------------------------PF-------------------RG--GGA----

------------------------------------------------PF----------

------------------------------------------------------------

------------------------------------------------------------

-----------------GIDV-----------------------------------PF--

--------------------------G----P---PGPD-MLPTF---------------

---------------VN----WSTYGAVT-------------------PIQDQG------

----DCGSCWAFGVTGLIEAAHFI-RN----------------KELIKLSEQHLIDG---

------NN------------------------LRNFGCKHG---------SCSEALD---

-----------YIMRN-----G-GIINAES----------------Y-PYKEAQEP----

---------VRSITV-------------HAGHLVS----------------PVSYRQLTS

YETRS-------------------------------------------------------

------------------------------------------------------------

------------------------------------------------------------

------------------------------------------------------------

------------------------------------------------------------

------------------------------------------------------------

------------------------------------------------------------

------------------------------------------------------------

------------------------------------------------------------

------------------------------------------------------------

------------------------------------------------------------

------------------------------------------------------------

------------------------------------------------------------

------------------------------------------------------------

------------------------------------------------------------

------------------------------------------------------------

>MER0001405

------------------------------------------------------------

------------------------------------------------------------

----------------------------------MVP-----------------------

------------------------RRL---------------------------------

-----------------------------FVLAVVVLADT--------------------

-------------------------------AAVV-------------------------

-----------------------------------------------NSGFADSNP----

------IRPVTD-----------RAASAL-------ESTVFAAL---------------G

RT-----------------------------RDALRFARFAVRY----------------

-------GK--------------------------------------------SY-----

-------------------------ESA-------------------------------A

EVHK--------------------------------------------------------

-----------RFRIFSES-----------------------------------------

------------------------------------------------------------

------------------------------LQLVRSTN---------R-KG----L----

------------------------------------------------SY----------

------------------------------------------------------------

---------------------------RLG--------------------------INRF

ADMSWEEFRATRL----GAAQ---NCSA-------------TLTGNHRM-----------

-------------------------------R---AAAV-ALPET---------------

---------------KD----WREDGIVS-------------------PVKNQG------

----HCGSCWTFSTTGALEAAYTQ-AT----------------GKPISLSEQQLVDC---

------GFA-----------------------FNNFGCNGG---------LPSQAFE---

-----------YIKYN-----G-GLDTEES----------------Y-PYQGVNGI----

---------CKFKN---------------ENVGVK----------------VLDSVNITL

GAEDE--------------LKDAVGLVRPVSVAFEVI-TGFRLYKS--------------

-----------------------GVYTS-----------------------------DHC

G-T-TPM-D-VNH-----------------------------------------------

-----------------------------AVLAVG-----------YG-VE---------

--------------DGVPY---------------W------LIKNSWGAD----------

WG-----------------------DEGYFK--MEMG-----------------------

-------------KNMCG-------------------------------VATC-------

-------------ASYP---------IVA-------------------------------

------------------------------------------------------------

------------------------------------------------------------

------------------------------------------------------------

------------------------------------------------------------

------------------------------------------------------------

------------------------------------------------------------

------------------------------------------------------------

------------------------------------------------------------

>MER0158387

------------------------------------------------------------

------------------------------------------------------------

----------------------------------MGG-----------------------

------------------------------------------------------------

------------------------------ALLLALLLVS--------------------

------------------------------------------------------------

------------------------------------------------------------

------------------------AAAAP-------QV----------------------

------------------------------------------------------------

------------------------------------------------------------

-------------------------LGV-------------------------------G

NGDN--------------------------------------------------------

-----------HMRIIQED-----------------------------------------

------------------------------------------------------------

--------------------------------IIETVN---------N-HP---SA----

------------------------------------------------GW----------

------------------------------------------------------------

---------------------------TASR-------------------------NPYF

SNYTIAQFKHIL-----GVKP---APQN-------------ALSNVPVKTY---------

-----------------------------------SRSL-ELPKE---------------

---------------FDARSAWSRCSTIG-------------------NILDQG------

----HCGSCWAFGAVECLQDRFCI-HL----------------NMSILLSVNDLLAC---

-----------------CGF------------MCGDGCDGG---------YPIEAWR---

-----------YFVQN-----G-VVTDECDPYFDPVGCKHPGCEPAY-PTPKCEKK----

---------CKEQNQ---VW--------QEKKHFS----------------IDAYR-INS

DPHDI--------------MAEVYK-NGPVEVAFTVY-EDFAHYKS--------------

-----------------------GVYKH------------------------------IT

G-G-----IMGGH-----------------------------------------------

-----------------------------AVKLIG-----------WGTSD---------

--------------AGEDY---------------W------LLANQWNRG----------

WG-----------------------DDGYFK--IIRG-----------------------

-------------KNECG-------------------------------IEEGV-V----

-------------AGMPS--------TKNMV-----------------------------

--------PNFGG-----------------------------------------------

------------------------------------------------------------

------------------------------------AV----------------------

--GRA-----------------------------IV------------------------

------------------------------------------------------------

------------------------------------------------------------

------------------------------------------------------------

------------------------------------------------------------

>CP1A

------------------------------------------------------------

------------------------------------------------------------

----------------------------------MAA-----------------------

----------------------STT----AAA----------------------------

-----------------------------ALLLLLLSLAA--------------------

-------------------------------AAD--------------------------

--------------------------------------------MS-IVSY---------

------------------------GERS--------EE----------------------

-------------------------------EARRMYAEWMAAH----------------

-------GR--------------------------------------------TY-----

-------------------------NAV-------------------------------G

EEER--------------------------------------------------------

-----------RYQVFRDN-----------------------------------------

------------------------------------------------------------

------------------------------LRYIDAHN---------A-AADAGVH----

------------------------------------------------SF----------

------------------------------------------------------------

---------------------------RLG--------------------------LNRF

ADLTNDEYRATYL----GART---RPQR-------------ERK----------------

----------------LG----ARY-H----A---ADNE-DLPES---------------

---------------VD----WRAKGAVA-------------------EVKDQG------

----SCGSCWAFSTIAAVEGINQI-VT----------------GDLISLSEQELVDC---

------DT------------------------SYNQGCNGG---------LMDYAFE---

-----------FIINN-----G-GIDTEKD----------------Y-PYKGTDGR----

---------CDVNRK--------------NAKVVT----------------IDSYEDVPA

NDEKS--------------LQKAVA-NQPVSVAIEAAGTAFQLYSS--------------

-----------------------GIFTG------------------------------SC

G-T-----A-LDH-----------------------------------------------

-----------------------------GVTAVG-----------YG-TE---------

--------------NGKDY---------------W------IVKNSWGSS----------

WG-----------------------ESGYVR--MERNIKA--------------------

-----------S-SGKCG-------------------------------IAVE-------

-------------PSYP---------LKE---------------------GANP------

--------PNPGP-------------------------------SPPSPTP---------

------------------------------------------------------------

---------------------------------APA------------------------

------------------------------------VCDNYY------------------

--------------------------------SCPDSTTCC-------------------

---------------------------------------------CIYEYGKYCFAWG-C

CPLEGATCCD----DHYSCCPHDYPICNVRQGTCLMGKDSPLSLS-----VKATKRTLAK

PHWAFSGNT----------------ADGMKSSA---------------------------

>CP1B

------------------------------------------------------------

------------------------------------------------------------

----------------------------------MGA-----------------------

----------------------STTPLASAAA----------------------------

-----------------------------LLLLLLAPLAA--------------------

-------------------------------AADS-------------------------

--------------------------------------------MS-IVSY---------

------------------------GERS--------EE----------------------

-------------------------------EARRMYAEWMAAH----------------

-------GR--------------------------------------------TY-----

-------------------------NAV-------------------------------G

EEER--------------------------------------------------------

-----------RFEVFRDN-----------------------------------------

------------------------------------------------------------

------------------------------LRYVDAHN---------A-AADAGVH----

------------------------------------------------SF----------

------------------------------------------------------------

---------------------------RLG--------------------------LNRF

ADLTNDEYRATYL----GVRS---RPQR-------------ERR----------------

----------------LG----DRY-L----A---GDNE-DLPES---------------

---------------VD----WRAKGAVA-------------------EIKDQG------

----SCGSCWAFSTIAAVEGINQI-VT----------------GDMISLSEQELVDC---

------DT------------------------SYNQGCNGG---------LMDYAFE---

-----------FIINN-----G-GIDTEED----------------Y-PYKGTDGR----

---------CDVNRK--------------NAKVVT----------------IDSYEDVPA

NSEKS--------------LQKAVA-NQPISVAIEAGGRAFQLYNS--------------

-----------------------GIFTG------------------------------TC

G-T-----A-LDH-----------------------------------------------

-----------------------------GVTAVG-----------YG-TE---------

--------------NGKDY---------------W------IVKNSWGSS----------

WG-----------------------ESGYVR--MERNIKA--------------------

-----------S-SGKCG-------------------------------IAVE-------

-------------PSYP---------LKK---------------------GANP------

--------PNPGP-------------------------------TPPSPTP---------

------------------------------------------------------------

---------------------------------PPT------------------------

------------------------------------VCDNYY------------------

--------------------------------SCPDSTTCC-------------------

---------------------------------------------CIYEYGKYCFAWG-C

CPLEGATCCD----DHYSCCPHDYPVCNVKQGTCLMGKDSPLSLS-----VKATKRTLAK

PHWAFSGNTA---------------ADGMKSSA---------------------------

>CP1C

------------------------------------------------------------

------------------------------------------------------------

----------------------------------MAA-----------------------

-----------------------LGRGLPL------------------------------

-----------------------------LLLLLLLAVSG--------------------

-------------------------------AANAAA-----------------------

---------------------------------------APGG-MS-IITYNEEHG----

---------ARG------------LERT--------EP----------------------

-------------------------------EVRAMYDLWLAEH----------------

-------GR--------------------------------------------AY-----

-------------------------NAL---------GEGE------------------G

ERDR--------------------------------------------------------

-----------RFLVFWDN-----------------------------------------

------------------------------------------------------------

------------------------------LRFVDAHN---------E-RA--GAR----

------------------------------------------------GF----------

------------------------------------------------------------

---------------------------RLG--------------------------MNQF

ADLTNDEFRAAYL----GAMV---PAAR-------------RGA----------------

---------------VVG----ERYRH----D---GAAE-ELPES---------------

---------------VD----WREKGAVA-------------------PVKNQG------

----QCGSCWAFSAVSSVESVNQI-VT----------------GEMVTLSEQELVEC---

------STD-----------------------GGNSGCNGG---------LMDAAFD---

-----------FIIKN-----G-GIDTEDD----------------Y-PYRAVDGK----

---------CDMNRK--------------NARVVS----------------IDGFEDVPE

NDEKS--------------LQKAVA-HQPVSVAIEAGGREFQLYKS--------------

-----------------------GVFSG------------------------------SC

T-T-----N-LDH-----------------------------------------------

-----------------------------GVVAVG-----------YG-AE---------

--------------NGKDY---------------W------IVRNSWGPK----------

WG-----------------------EAGYIR--MERNVNA--------------------

-----------S-TGKCG-------------------------------IAMM-------

-------------ASYP---------TKK---------------------GANP------

--------PRPSP-------------------------------TPPT------------

------------------------------------------------------------

---------------------------------PPAAPDN--------------------

------------------------------------VCDENF------------------

--------------------------------SCSAGSTCC-------------------

---------------------------------------------CAFGFRNVCLVWG-C

CPVEGATCCK----DHASCCPPGYPVCNVRAGTCSVSKNSPLS-------VKALKRTLAK

LSTA--------------------------------------------------------

>CatB

------------------------------------------------------------

------------------------------------------------------------

----------------------------------MGG-----------------------

------------------------------------------------------------

------------------------------ALLLALLLVS--------------------

------------------------------------------------------------

------------------------------------------------------------

------------------------AAAAP-------QV----------------------

------------------------------------------------------------

------------------------------------------------------------

-------------------------LGV-------------------------------G

NGDN--------------------------------------------------------

-----------HMRIIQED-----------------------------------------

------------------------------------------------------------

--------------------------------IIETVN---------N-HP---SA----

------------------------------------------------GW----------

------------------------------------------------------------

---------------------------TASR-------------------------NPYF

SNYTIAQFKHIL-----GVKP---APQN-------------ALSNVPVKTY---------

-----------------------------------SRSL-ELPKE---------------

---------------FDARSAWSRCSTIG-------------------NILDQG------

----HCGSCWAFGAVECLQDRFCI-HL----------------NMSILLSVNDLLAC---

-----------------CGF------------MCGDGCDGG---------YPIEAWR---

-----------YFVQN-----G-VVTDECDPYFDPVGCKHPGCEPAY-PTPKCEKK----

---------CKEQNQ---VW--------QEKKHFS----------------IDAYR-INS

DPHDI--------------MAEVYK-NGPVEVAFTVY-EDFAHYKS--------------

-----------------------GVYKH------------------------------IT

G-G-----IMGGH-----------------------------------------------

-----------------------------AVKLIG-----------WGTSD---------

--------------AGEDY---------------W------LLANQWNRG----------

WG-----------------------DDGYFK--IIRG-----------------------

-------------KNECG-------------------------------IEEGV-V----

-------------AGMPS--------TKNMV-----------------------------

--------PNFGG-----------------------------------------------

------------------------------------------------------------

------------------------------------AV----------------------

--GRA-----------------------------IV------------------------

------------------------------------------------------------

------------------------------------------------------------

------------------------------------------------------------

------------------------------------------------------------

>CP2

------------------------------------------------------------

------------------------------------------------------------

----------------------------------MVP-----------------------

------------------------RRL---------------------------------

-----------------------------LVLAVLALAAT--------------------

-------------------------------AAAA-------------------------

-----------------------------------------------NSGFADSNP----

------IRPVTD-----------RAASAL-------ESTVFAAL---------------G

RT-----------------------------RDALRFARFAVRY----------------

-------GK--------------------------------------------SY-----

-------------------------ESA-------------------------------A

EVHK--------------------------------------------------------

-----------RFRIFSES-----------------------------------------

------------------------------------------------------------

------------------------------LQLVRSTN---------R-KG----L----

------------------------------------------------SY----------

------------------------------------------------------------

---------------------------RLG--------------------------INRF

ADMSWEEFRATRL----GAAQ---NCSA-------------TLTGNHRM-----------

-------------------------------R---AAAV-ALPET---------------

---------------KD----WREDGIVS-------------------PVKNQG------

----HCGSCWTFSTTGALEAAYTQ-AT----------------GKPISLSEQQLVDC---

------GFA-----------------------FNNFGCNGG---------LPSQAFE---

-----------YIKYN-----G-GLDTEES----------------Y-PYQGVNGI----

---------CKFKN---------------ENVGVK----------------VLDSVNITL

GAEDE--------------LKDAVGLVRPVSVAFEVI-TGFRLYKS--------------

-----------------------GVYTS-----------------------------DHC

G-T-TPM-D-VNH-----------------------------------------------

-----------------------------AVLAVG-----------YG-VE---------

--------------DGVPY---------------W------LIKNSWGAD----------

WG-----------------------DEGYFK--MEMG-----------------------

-------------KNMCG-------------------------------VATC-------

-------------ASYP---------IVA-------------------------------

------------------------------------------------------------

------------------------------------------------------------

------------------------------------------------------------

------------------------------------------------------------

------------------------------------------------------------

------------------------------------------------------------

------------------------------------------------------------

------------------------------------------------------------

>XCP2_1

------------------------------------------------------------

------------------------------------------------------------

----------------------------------MAWS----------------------

--------------------CARPMSIALAAV----------------------------

-----------------------------LLLCGGAWLQQ--------------------

-------------------------------AAEARPHH---------------------

------------MDDDSSI----------------DMDRGSDDFFS-IVGYSPE------

------------------------DLTQ--------HD----------------------

-------------------------------RLVRLFEEWVAKY----------------

-------RK--------------------------------------------AY-----

-------------------------GSF-------------------------------E

EKLR--------------------------------------------------------

-----------RFEVFKDN-----------------------------------------

------------------------------------------------------------

------------------------------LHHIDEAN---------R-KE---VT----

------------------------------------------------SY----------

------------------------------------------------------------

---------------------------WLG--------------------------LNAF

ADLTHDEFKATYL----GLLP---KRTS-------------GGR----------------

----------------------FRY-G--GVG---DGGD-EVPAS---------------

---------------VD----WRKKGAVT-------------------EVKNQG------

----QCGSCWAFSTVAAVEGINQI-VT----------------GNLTSLSEQQLVDC---

------ST------------------------DGNNGCSGG---------VMDNAFS---

-----------FIATG-----A-GLRSEEA----------------Y-PYLMEEGD----

---------CDDRAR-------------DGEVLVT----------------ISGYEDVPA

NDEQA--------------LVKALA-HQPVSVAIEASGRHFQFYSG--------------

-----------------------GVFDG------------------------------PC

G-S-----E-LDH-----------------------------------------------

-----------------------------GVAAVG-----------YG-SS---------

--------------KGQDY---------------I------IVKNSWGTH----------

WG-----------------------EKGYIR--MKRGTGK--------------------

-----------P-EGLCG-------------------------------INKM-------

-------------ASYP---------TKDH------------------------------

------------------------------------------------------------

------------------------------------------------------------

------------------------------------------------------------

------------------------------------------------------------

------------------------------------------------------------

------------------------------------------------------------

------------------------------------------------------------

------------------------------------------------------------

>RD21

------------------------------------------------------------

------------------------------------------------------------

----------------------------------MGF-----------------------

--------------------------LKPTMA----------------------------

-----------------------------ILFLAMVAVSS--------------------

-------------------------------AVD--------------------------

--------------------------------------------MS-IISYDEKHG----

---------VST-----------TGGRS--------EA----------------------

-------------------------------EVMSIYEAWLVKH----------------

-------GKAQ------------------------------------------SQ-----

-------------------------NSL-------------------------------V

EKDR--------------------------------------------------------

-----------RFEIFKDN-----------------------------------------

------------------------------------------------------------

------------------------------LRFVDEHN---------E-KN----L----

------------------------------------------------SY----------

------------------------------------------------------------

---------------------------RLG--------------------------LTRF

ADLTNDEYRSKYL----GAKM---EKKG-------------ERR----------------

----------------TS----LRY-E----A---RVGD-ELPES---------------

---------------ID----WRKKGAVA-------------------EVKDQG------

----GCGSCWAFSTIGAVEGINQI-VT----------------GDLITLSEQELVDC---

------DT------------------------SYNEGCNGG---------LMDYAFE---

-----------FIIKN-----G-GIDTDKD----------------Y-PYKGVDGT----

---------CDQIRK--------------NAKVVT----------------IDSYEDVPT

YSEES--------------LKKAVA-HQPISIAIEAGGRAFQLYDS--------------

-----------------------GIFDG------------------------------SC

G-T-----Q-LDH-----------------------------------------------

-----------------------------GVVAVG-----------YG-TE---------

--------------NGKDY---------------W------IVRNSWGKS----------

WG-----------------------ESGYLR--MARNIAS--------------------

-----------S-SGKCG-------------------------------IAIE-------

-------------PSYP---------IKN---------------------GENP------

--------PNPGP-------------------------------SPPSPIK---------

------------------------------------------------------------

---------------------------------PPT------------------------

------------------------------------QCDSYY------------------

--------------------------------TCPESNTCC-------------------

---------------------------------------------CLFEYGKYCFAWG-C

CPLEAATCCD----DNYSCCPHEYPVCDLDQGTCLLSKNSPFS-------VKALKRKPAT

PFW----------------------SQGRKNIA---------------------------

>CEP1

------------------------------------------------------------

------------------------------------------------------------

----------------------------------MKRF----------------------

------------------------------------------------------------

-----------------------------IVLALCMLMVL--------------------

-----------------------------ET-----------------------------

--------------------------------------------TK-GLDFHNK------

------------------------DVES--------EN----------------------

-------------------------------SLWELYERWRSHH----------------

----------------------------------------------------TVA-----

-------------------------RSL-------------------------------E

EKAK--------------------------------------------------------

-----------RFNVFKHN-----------------------------------------

------------------------------------------------------------

------------------------------VKHIHETN---------K-KD----K----

------------------------------------------------SY----------

------------------------------------------------------------

---------------------------KLK--------------------------LNKF

GDMTSEEFRRTYA----GSNI---KHHR-------------MFQGEKKA-----------

---------------TKS----FMY----------ANVN-TLPTS---------------

---------------VD----WRKNGAVT-------------------PVKNQG------

----QCGSCWAFSTVVAVEGINQI-RT----------------KKLTSLSEQELVDC---

------DT------------------------NQNQGCNGG---------LMDLAFE---

-----------FIKEK-----G-GLTSELV----------------Y-PYKASDET----

---------CDTNKE--------------NAPVVS----------------IDGHEDVPK

NSEDD--------------LMKAVA-NQPVSVAIDAGGSDFQFYSE--------------

-----------------------GVFTG------------------------------RC

G-T-----E-LNH-----------------------------------------------

-----------------------------GVAVVG-----------YGTTI---------

--------------DGTKY---------------W------IVKNSWGEE----------

WG-----------------------EKGYIR--MQRGIRH--------------------

-----------K-EGLCG-------------------------------IAME-------

-------------ASYP---------LKN---------------------SNTN------

--------P--------------------------------------SRL----------

------------------------------------------------------------

------------------------------------------------------------

------------------------------------SLD---------------------

--------------------------------SLKDEL----------------------

------------------------------------------------------------

------------------------------------------------------------

------------------------------------------------------------

>XCP2_2

------------------------------------------------------------

------------------------------------------------------------

----------------------------------MAL-----------------------

---------------------SSPSRILCFAL----------------------------

-----------------------------ALSAASLSLSF--------------------

-------------------------------ASSH-------------------------

------------------------------------------D-YS-IVGYSPE------

------------------------DLES--------HD----------------------

-------------------------------KLIELFENWISNF----------------

-------EK--------------------------------------------AY-----

-------------------------ETV-------------------------------E

EKFL--------------------------------------------------------

-----------RFEVFKDN-----------------------------------------

------------------------------------------------------------

------------------------------LKHIDETN---------K-KG----K----

------------------------------------------------SY----------

------------------------------------------------------------

---------------------------WLG--------------------------LNEF

ADLSHEEFKKMYL----GLKT---DIVR-------------RDEERSYAE----------

----------------------FAY----------RDVE-AVPKS---------------

---------------VD----WRKKGAVA-------------------EVKNQG------

----SCGSCWAFSTVAAVEGINKI-VT----------------GNLTTLSEQELIDC---

------DT------------------------TYNNGCNGG---------LMDYAFE---

-----------YIVKN-----G-GLRKEED----------------Y-PYSMEEGT----

---------CEMQKD--------------ESETVT----------------INGHQDVPT

NDEKS--------------LLKALA-HQPLSVAIDASGREFQFYSG--------------

-----------------------GVFDG------------------------------RC

G-V-----D-LDH-----------------------------------------------

-----------------------------GVAAVG-----------YG-SS---------

--------------KGSDY---------------I------IVKNSWGPK----------

WG-----------------------EKGYIR--LKRNTGK--------------------

-----------P-EGLCG-------------------------------INKM-------

-------------ASFP---------TKTK------------------------------

------------------------------------------------------------

------------------------------------------------------------

------------------------------------------------------------

------------------------------------------------------------

------------------------------------------------------------

------------------------------------------------------------

------------------------------------------------------------

------------------------------------------------------------

>XBCP3

------------------------------------------------------------

------------------------------------------------------------

----------------------------------MSM-----------------------

-----------------------SSSSFI-------------------------------

-----------------------------SLTFFFLLLVS--------------------

------------------------------------------------------------

------------------------------------------------------------

------------------------SSSS--------SD----------------------

-------------------------------DISELFDDWCQKH----------------

-------GK--------------------------------------------TY-----

-------------------------GSE-------------------------------E

ERQQ--------------------------------------------------------

-----------RIQIFKDN-----------------------------------------

------------------------------------------------------------

------------------------------HDFVTQHN---------L-IT---NA----

------------------------------------------------TY----------

------------------------------------------------------------

---------------------------SLS--------------------------LNAF

ADLTHHEFKASRL----GLSV---SAPS-------------VIMASKGQ-----------

--------------------------S----L---GGSV-KVPDS---------------

---------------VD----WRKKGAVT-------------------NVKDQG------

----SCGACWSFSATGAMEGINQI-VT----------------GDLISLSEQELIDC---

------DK------------------------SYNAGCNGG---------LMDYAFE---

-----------FVIKN-----H-GIDTEKD----------------Y-PYQERDGT----

---------CKKDKL--------------KQKVVT----------------IDSYAGVKS

NDEKA--------------LMEAVA-AQPVSVGICGSERAFQLYSS--------------

-----------------------GIFSG------------------------------PC

S-T-----S-LDH-----------------------------------------------

-----------------------------AVLIVG-----------YG-SQ---------

--------------NGVDY---------------W------IVKNSWGKS----------

WG-----------------------MDGFMH--MQRNTEN--------------------

-----------S-DGVCG-------------------------------INML-------

-------------ASYP---------IKT---------------------HPNP------

--------PPPSP---------------------------------PGPT----------

------------------------------------------------------------

------------------------------------------------------------

------------------------------------KCNLFT------------------

--------------------------------YCSSGETCC-------------------

---------------------------------------------CARELFGLCFSWK-C

CEIESAVCCK----DGRHCCPHDYPVCDTTRSLCLKKTGNFTA-------IKPFWKKNS-

-------------------------SKQLGRFEEWVM-----------------------

>THI1

------------------------------------------------------------

------------------------------------------------------------

----------------------------------MLNV----------------------

---------------------LRNSNLTL-------------------------------

-----------------------------AVLICFVLIASK-------------------

------------------------------------------------------------

-----------------------------------------------LCSVDSS------

------------------------VYDP--------HK----------------------

-------------------------------TLKQRFEKWLKTH----------------

-------SK--------------------------------------------LY-----

-------------------------GGR-------------------------------D

EWML--------------------------------------------------------

-----------RFGIYQSN-----------------------------------------

------------------------------------------------------------

------------------------------VQLIDYIN---------S-----LHL----

------------------------------------------------PF----------

------------------------------------------------------------

---------------------------KLT--------------------------DNRF

ADMTNSEFKAHFL----GLNT---SSLR-------------LHKKQRPV-----------

-------------------------------C---DPAG-NVPDA---------------

---------------VD----WRTQGAVT-------------------PIRNQG------

----KCGGCWAFSAVAAIEGINKI-KT----------------GNLVSLSEQQLIDC---

------DVG-----------------------TYNKGCSGG---------LMETAFE---

-----------FIKTN-----G-GLATETD----------------Y-PYTGIEGT----

---------CDQEKS--------------KNKVVT----------------IQGYQKV-A

QNEAS--------------LQIAAA-QQPVSVGIDAGGFIFQLYSS--------------

-----------------------GVFTN------------------------------YC

G-T-----N-LNH-----------------------------------------------

-----------------------------GVTVVG-----------YGVEG---------

---------------DQKY---------------W------IVKNSWGTG----------

WG-----------------------EEGYIR--MERGVSE--------------------

-----------D-TGKCG-------------------------------IAMM-------

-------------ASYP---------LQ--------------------------------

------------------------------------------------------------

------------------------------------------------------------

------------------------------------------------------------

------------------------------------------------------------

------------------------------------------------------------

------------------------------------------------------------

------------------------------------------------------------

------------------------------------------------------------

>SAG12

------------------------------------------------------------

------------------------------------------------------------

----------------------------------MALKHMQIF-----------------

------------------------------------------------------------

-----------------------------LFVAIFSSFCF--------------------

-------------------------------SITLSR-----------------------

------------------------------------------------------------

------------------------PLDN--------EL----------------------

-------------------------------IMQKRHIEWMTKH----------------

-------GR--------------------------------------------VY-----

-------------------------ADV-------------------------------K

EENN--------------------------------------------------------

-----------RYVVFKNN-----------------------------------------

------------------------------------------------------------

------------------------------VERIEHLN---------SIPA---GR----

------------------------------------------------TF----------

------------------------------------------------------------

---------------------------KLA--------------------------VNQF

ADLTNDEFRSMYT----GFKG---VSAL-------------SSQSQTK------------

---------------MSP----FRY-Q----N---VSSG-ALPVS---------------

---------------VD----WRKKGAVT-------------------PIKNQG------

----SCGCCWAFSAVAAIEGATQI-KK----------------GKLISLSEQQLVDC---

------DTN-------------------------DFGCEGG---------LMDTAFE---

-----------HIKAT-----G-GLTTESN----------------Y-PYKGEDAT----

---------CNSKKT--------------NPKATS----------------ITGYEDVPV

NDEQA--------------LMKAVA-HQPVSVGIEGGGFDFQFYSS--------------

-----------------------GVFTG------------------------------EC

T-T-----Y-LDH-----------------------------------------------

-----------------------------AVTAIG-----------YGEST---------

--------------NGSKY---------------W------IIKNSWGTK----------

WG-----------------------ESGYMR--IQKDVKD--------------------

-----------K-QGLCG-------------------------------LAMK-------

-------------ASYP---------TI--------------------------------

------------------------------------------------------------

------------------------------------------------------------

------------------------------------------------------------

------------------------------------------------------------

------------------------------------------------------------

------------------------------------------------------------

------------------------------------------------------------

------------------------------------------------------------

>RD19A

------------------------------------------------------------

------------------------------------------------------------

----------------------------------MDRLKLYF------------------

------------------------------------------------------------

-----------------------------SVFVLSFFIVS--------------------

-------------------------------VSSSDVNDG--------------------

------------------------------------------------------------

--DDLVIRQVVG------------GAEP--------QV----------------------

------------------------------LTSEDHFSLFKRKF----------------

-------GK--------------------------------------------VY-----

-------------------------ASN-------------------------------E

EHDY--------------------------------------------------------

-----------RFSVFKAN-----------------------------------------

------------------------------------------------------------

------------------------------LRRARRHQ---------K-LD----P----

------------------------------------------------SA----------

------------------------------------------------------------

---------------------------THG--------------------------VTQF

SDLTRSEFRKKHL----GVRS---GFK--------------LPKDANKAPI---------

-----------------------------------LPTE-NLPED---------------

---------------FD----WRDHGAVT-------------------PVKNQG------

----SCGSCWSFSATGALEGANFL-AT----------------GKLVSLSEQQLVDC---

------DHECDPEEAD----------------SCDSGCNGG---------LMNSAFE---

-----------YTLKT-----G-GLMKEED----------------Y-PYTGKDGK----

--------TCKLDK---------------SKIVAS----------------VSNFSVISI

DEEQI--------------AANLVK-NGPLAVAINAG--YMQTYIG--------------

-----------------------GVSCP-----------------------------YIC

T-R-----R-LNH-----------------------------------------------

-----------------------------GVLLVG-----------YGAAGYAP------

-----------ARFKEKPY---------------W------IIKNSWGET----------

WG-----------------------ENGFYK--ICKG-----------------------

-------------RNICG-------------------------------VDSM-------

-------------VSTV---------AATV--------------------STTAH-----

------------------------------------------------------------

------------------------------------------------------------

------------------------------------------------------------

------------------------------------------------------------

------------------------------------------------------------

------------------------------------------------------------

------------------------------------------------------------

------------------------------------------------------------

>AALP

------------------------------------------------------------

------------------------------------------------------------

----------------------------------MSA-----------------------

------------------------KTIL--------------------------------

-----------------------------SSVVLVVLVAA--------------------

-------------------------------SAAA-------------------------

-----------------------------------------------NIGFDESNP----

------IRMVSD------------GLREV-------EESVSQIL---------------G

QS-----------------------------RHVLSFARFTHRY----------------

-------GK--------------------------------------------KY-----

-------------------------QNV-------------------------------E

EMKL--------------------------------------------------------

-----------RFSIFKEN-----------------------------------------

------------------------------------------------------------

------------------------------LDLIRSTN---------K-KG----L----

------------------------------------------------SY----------

------------------------------------------------------------

---------------------------KLG--------------------------VNQF

ADLTWQEFQRTKL----GAAQ---NCSA-------------TLKGSHK------------

-----------------------------------VTEA-ALPET---------------

---------------KD----WREDGIVS-------------------PVKDQG------

----GCGSCWTFSTTGALEAAYHQ-AF----------------GKGISLSEQQLVDC---

------AGA-----------------------FNNYGCNGG---------LPSQAFE---

-----------YIKSN-----G-GLDTEKA----------------Y-PYTGKDET----

---------CKFSA---------------ENVGVQ----------------VLNSVNITL

GAEDE--------------LKHAVGLVRPVSIAFEVI-HSFRLYKS--------------

-----------------------GVYTD-----------------------------SHC

G-S-TPM-D-VNH-----------------------------------------------

-----------------------------AVLAVG-----------YG-VE---------

--------------DGVPY---------------W------LIKNSWGAD----------

WG-----------------------DKGYFK--MEMG-----------------------

-------------KNMCG-------KYCY--------------------MCII-------

----------------P-------------------------------------------

------------------------------------------------------------

------------------------------------------------------------

------------------------------------------------------------

------------------------------------------------------------

------------------------------------------------------------

--------------------------------------------RC--------------

------------------------------------------------------------

---------------------------GLR------------------------------

>CTB3

------------------------------------------------------------

------------------------------------------------------------

----------------------------------MAV-----------------------

---------------------YNTKLCLAS------------------------------

-----------------------------VFLLLGLLLAF--------------------

------------------------------------------------------------

------------------------------------------------------------

------------------------DLKGI-------EA----------------------

------------------------------------------------------------

------------------------------------------------------------

-------------------------ESL-------------------------------T

KQKL--------------------------------------------------------

-----------DSKILQDE-----------------------------------------

------------------------------------------------------------

--------------------------------IVKKVN---------E-NP---NA----

------------------------------------------------GW----------

------------------------------------------------------------

---------------------------KAAI-------------------------NDRF

SNATVAEFKRLL-----GVKP---TPKK-------------HFLGVPIVSH---------

-----------------------------------DPSL-KLPKA---------------

---------------FDARTAWPQCTSIG-------------------NILGLG------

----HCGSCWAFGAVESLSDRFCI-QF----------------GMNISLSVNDLLAC---

-----------------CGF------------RCGDGCDGG---------YPIAAWQ---

-----------YFSYS-----G-VVTEECDPYFDNTGCSHPGCEPAY-PTPKCSRK----

---------CVSDNK---LW--------SESKHYS----------------VSTYT-VKS

NPQDI--------------MAEVYK-NGPVEVSFTVY-EDFAHYKS--------------

-----------------------GVYKH------------------------------IT

G-S-----NIGGH-----------------------------------------------

-----------------------------AVKLIG-----------WGTSS---------

--------------EGEDY---------------W------LMANQWNRG----------

WG-----------------------DDGYFM--IRRG-----------------------

-------------TNECG-------------------------------IEDEP-V----

-------------AGLPS--------SKNVFRVDT---------------GSNDL-----

--------PVASV-----------------------------------------------

------------------------------------------------------------

------------------------------------------------------------

------------------------------------------------------------

------------------------------------------------------------

------------------------------------------------------------

------------------------------------------------------------

------------------------------------------------------------

>DEGP2

------------------------------------------------------------

------------------------------------------------------------

----------------------------------MAA-----------------------

----------------------SVANCCFSVLNA--------------------------

-----------------------------SVKIQSSSISS--------------------

-P-----------------WCFVSASSLTPRASSNIKRKSSRSD----------------

---------------SPSPI------------------------LNPEKNYPGRV-----

-----------------------RDESS--------NP----------------------

-------------------------------PQKMAFKAFGSPK----------------

-------KE--------------------------------------------KK-----

-------------------------ESL-------------------------------S

DFSR---------------------------DQQTDPAKIHDASF---------------

--------LNAVVKVYCTH--------------------TAPDYS---------------

------------------------------------------------------------

---------------------------LP-WQKQRQFT---------S-TG---------

-----------------------------------------------SAF----------

------------------------------------------------------------

---------------------------MIGD----GKLLTNAHC---------------V

EHDTQVKVKRR------GDDR---------------------------------------

-----------------------KYVA----KVLVRGVDCDIALL---------------

--------------SVESEDFWKGAEPLRLGH--------------LPRLQD--------

----------SVTVVGYPLGGDTISVT----------------KGVVSRIEVTSYAH---

-----GSSDLLGIQID---------------AAINPGNSGG-PAFNDQGECIGVAFQ---

VYRSEETENIGYVIPT-TVVSHFLTDYERN--------------GKYTGYPCLGVLLQKL

ENPALREC-LKVPTN-EGVLVRRVEPTSDASKVLKEGDV------------IVSFDDLHV

GCEGTVPFRSSERIAFRYLISQKFA-GDIAEIGIIRAGEHKKVQVVLRPRVHLVPYHIDG

GQP---------SYIIVAG----LVFTP-----------LSEPLIE-----------EEC

EDT-IGL-KLLTK-----------------A--------------RYSVARF---RGE--

-----------------------------QIVILSQVLANEVNIG-YEDMNNQQ------

----------VLKFNGIPIRNIHHLAH--------------LIDMCKDKY----------

----LVFEF----------------EDNYVAV-LEREASN--------------------

-----------S-ASLCILK-----DYG---------------------IPSERSAD---

-------------LLEPYV-----DPIDDTQALDQ---------------GIGDS-----

--------PVSNL-----------------------------------------------

------------------------------------------------------------

------------------------------------------------------------

------------------------------------------------------------

------------------------------------------------------------

------------------------------------------------------------

------------------------------------------------------------

--------------------------------------------------EIGFDGLVWA

>DGP11

------------------------------------------------------------

------------------------------------------------------------

------------------------------------M-----------------------

----------------------FFRPCVHTVGRY--------------------------

-----------------------------SRARVPGLLSS--------------------

--------------------LFFY------RSCNNVLTNSL-------------------

----------------------------------------------PTVTTAGRVSRY-G

YI--------------------CRRSST--------SA----------------------

-------------------------------AERGVFLPFALTC----------------

-------RR--------------------------------------------NI-----

-------------------------HSI-------------------------------H

EDEK----------------------KLERW-KKIEESHPLDELV---------------

--------LDSVVKVFSNS--------------------TEYSKS---------------

------------------------------------------------------------

---------------------------KP-WKTLDQKS---------S-RG---------

-----------------------------------------------TGF----------

------------------------------------------------------------

---------------------------AIAG----RKILTNAHV------------VMAM

NDHTFVDVKRH------GSQI---------------------------------------

-----------------------KYKA----KVQKISHECDLAIL---------------

--------------EIDSDEFWKGMNPLELGD--------------IPPLQE--------

----------VVSVV----GGENICIT----------------KGLVLRVETRIYDY---

-----SDSDLLSIQID---------------ATINDENSGG-PVIMGN-KVVGVVYE---

---------IGFVIPT-PIIKHFITSVQES--------------RQYSCFGSLDLSYQSL

ENVQIRNH-FKMSHEMTGILINKINSSSGAYKILRKDDI------------ILAIDGVPI

GNDEKVPFQNKRRIDFSYLVSMKKP-GEKALVKVLRNGKEYEYNISLKPVKPNFTVQQFY

NVP---------SYYIFGG----FVFVP-----------LTKTYLD-----------SE-

-----------HH-----------------------------------------------

------------------------------QVKISERLADDINEG-YQSLYGAQ------

----------VEKVNGVEVKNLKHLCE--------------LIEECSTED----------

----LRLEF----------------KNHKVLV-LNYESAK--------------------

-----------K-ATLQILE-----RHK---------------------IKSVISKD---

-------------ICLPMLL---DDPFKDNK--------------------INLL-----

--------PWSVL-----------------------------------------------

------------------------------------------------------------

------------------------------------------------------------

------------------------------------------------------------

------------------------------------------------------------

------------------------------------------------------------

------------------------------------------------------------

--------------------------------------------------PLMFD---FS
