# Supplementary material for: Proteases Underground: Analysis of the Maize Root Apoplast Identifies Organ Specific Papain-Like Cysteine Protease Activity
Source: Front Plant Sci. 2019 Apr 30;10:473. doi: 10.3389/fpls.2019.00473 (PMC6503450; doi:10.3389/fpls.2019.00473)
Supplement: Supplementary file 6 [file Data_Sheet_1.PDF]

Supplementary information

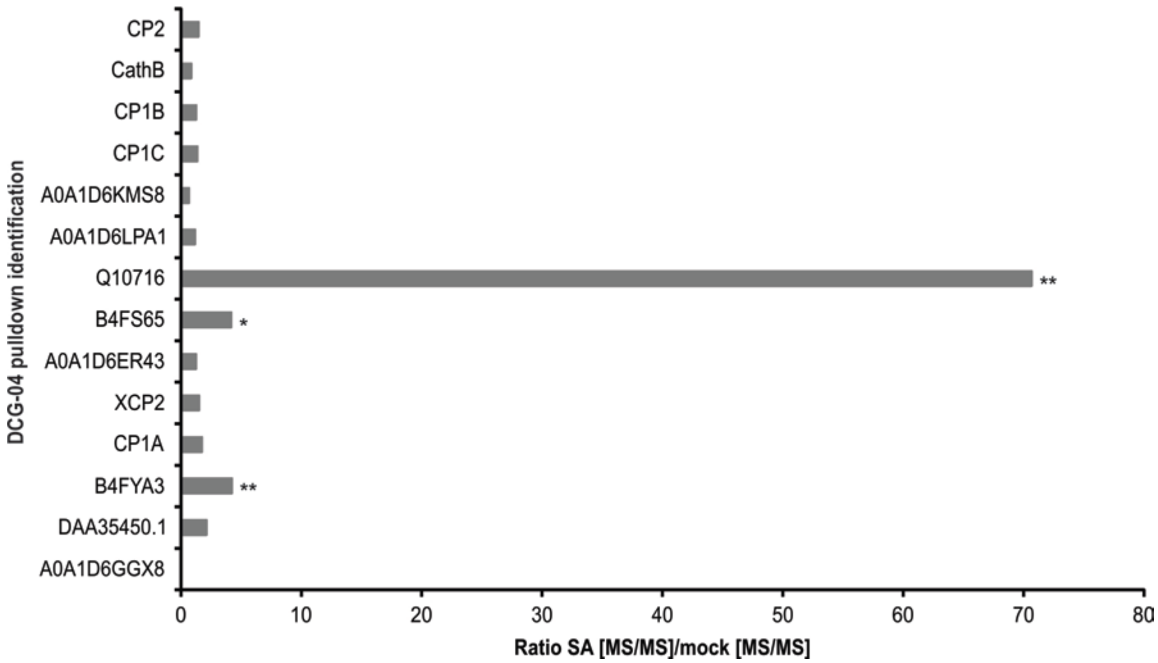

Supplementary figure 1. **Relative MS/MS-counts of DCG-04 labelled PLCPs.** Roots of maize plants were treated with 2 mM SA or mock. Apoplastic fluid was isolated 12 h after treatment. Active PLCPs were labelled with DCG-04, pulled down on streptavidin beads and subsequently on bead digested (OBD; trypsin). The tryptic peptides were then analyzed by LC-MS/MS. The ratio of the number of measured MS/MS spectra (MS/MS-counts) after SA treatment compared against the number of MS/MS counts in a mock treated sample was calculated and plotted. This experiment was performed in four independent biological replicates.

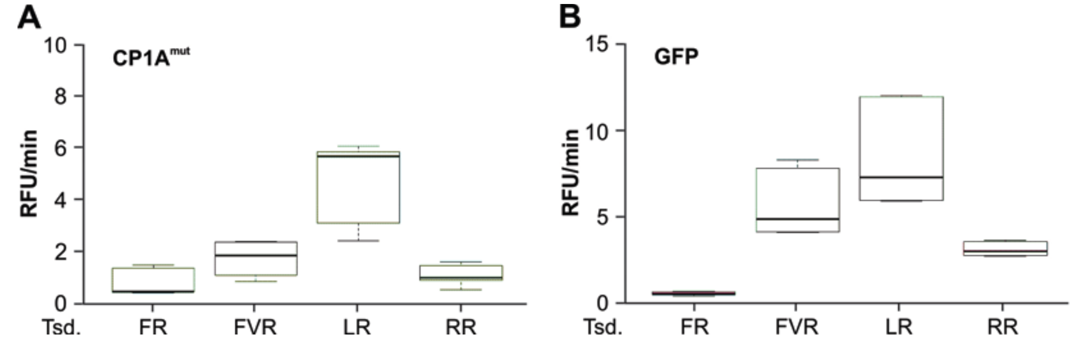

Supplementary figure 2. **Substrate cleavage assay of the catalytic inactive PLCP CP1A^{mut} and GFP.** Apoplastic fluids of *N. benthamiana* overexpressed CP1A^{mut} (A) and GFP (B) were tested for their activity using 10 µM of the following substrates: Z-FR-AMC (FR), BZ-FVR-AMC (FVR), Z-LR-AMC (LR) and Z-RR-AMC (RR). The release of AMC (relative fluorescent unit = RFU) per minute was measured and plotted for each substrate. The box signifies the upper (Q3) and lower (Q1) quartiles, and the median is represented by a short black line within the box for each substrate. Lower and upper whiskers represent Q1-1.5 x IQR and Q3+1.5 x IQR, respectively. This experiment was performed using three independent biological replicates each with technical duplicates.

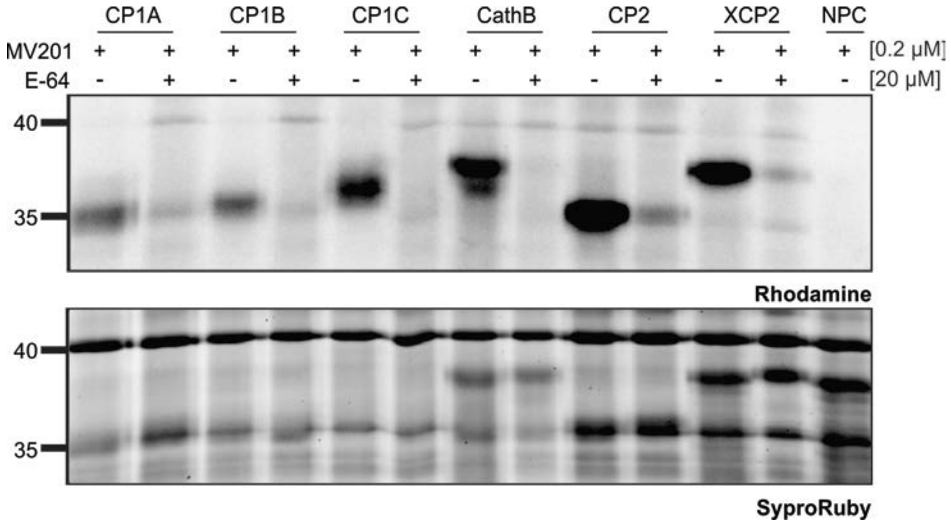

Supplementary figure 3. MV201 labeling of recombinant PLCPs. Samples were pre-treated for 30 min either with 20 µM E-64 (+) or DMSO (-) followed by labeling with 0.2 µM MV201 for 2 h at room temperature. Samples were separated by SDS-PAGE and labeled PLCPs were visualized by fluorescence scanning (Ex. 532 nm, Em. 580 nm). A No-Probe-Control (NPC) was included containing an equal mix of apoplstic fluid, neither treated with E-64, nor with MV201. SyproRuby-staining (Ex. 450 nm, Em. 610 nm) was performed as loading control. Fluorescence quantification was performed to calculate signal intensities of PLCPs. Based on this, samples were normalized to equal amounts of active PLCPs for subsequent assays.

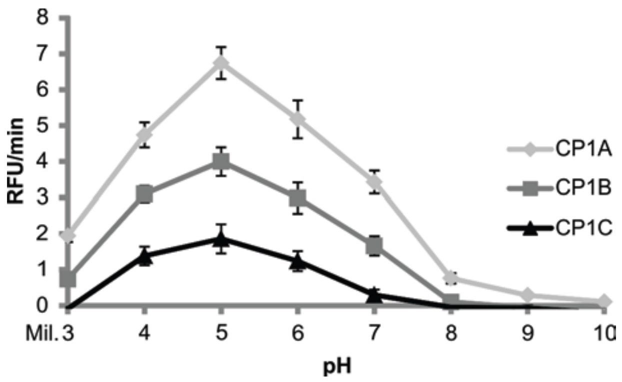

Supplementary figure 4. **pH dependency of CP1-like proteases.** Apoplastic fluids of *N. benthamiana* overexpressing PLCPs, CP1A (grey), CP1B (dark grey) and CP1C (black) were tested for their activity at different pH (3 to 10) using 10 µM of the substrate Z-LR-AMC. The release of AMC (relative fluorescent unit = RFU) per minute was measured and plotted against pH. Error bars represent the SEM. The experiment was performed in three independent biological replicates using technical duplicates.

Supplementary table 1: Identified peptides of shotgun analysis.

Supplementary table 2: Identified peptides after DCG-04 labelling.

Supplementary table 3: Primers used in this study.

Supplementary table 4: Strains used in this study.
